# Elevated ERβ expression driven by low ASB8-mediated ubiquitination in lung adenocarcinoma promotes lymph node metastasis via tumor-associated neutrophils

**DOI:** 10.1038/s41419-025-07870-z

**Published:** 2025-07-30

**Authors:** Yangwei Wang, Shiwen He, Mingxin Diao, Rong Zhao, Jinghua Ren, Peiyuan Mei, Shihao Wu, Sheng Fan, Yongde Liao

**Affiliations:** 1 Department of Thoracic Surgery, Union Hospital, Tongji Medical College, Huazhong University of Science and Technology, Wuhan, China; 2 Cancer Center, Union Hospital, Tongji Medical College, Huazhong University of Science and Technology, Wuhan, China; 3 Department of Thoracic Surgery, The First Affiliated Hospital of Xiamen University, Xiamen University, Xiamen, China

**Keywords:** Cancer microenvironment, Non-small-cell lung cancer

## Abstract

This study investigated the role of estrogen receptor beta (ERβ) in the lymph node metastasis of lung adenocarcinoma (LUAD), focusing on its interaction with tumor-associated neutrophils (TANs) and its regulation of lymphangiogenesis. Clinical analysis of LUAD patient samples revealed that high ERβ expression was correlated with positive lymph node metastasis and increased lymphatic vessel density. In vitro experiments showed that ERβ promotes neutrophil chemotaxis by regulating CCL15 transcription, whereas TANs secrete VEGF-C, enhancing lymphangiogenesis. Using an orthotopic lung cancer model, we confirmed that ERβ facilitates LUAD lymph node metastasis through TAN recruitment, and inhibiting neutrophils with anti-Ly6G antibodies or CCR1 antagonists reduced this effect. Additionally, the study found that ASB8, an E3 ubiquitin ligase, degrades ERβ through K48-linked polyubiquitination. Low ASB8 expression results in increased ERβ stability and promotes LUAD metastasis. These findings suggest that ERβ, by recruiting TANs through the CCL15-CCR1 axis, plays a key role in LUAD lymph node metastasis, with ASB8 acting as a crucial regulator of ERβ stability. Targeting ERβ and ASB8 could offer new therapeutic strategies for LUAD metastasis, warranting further investigation of their clinical applications.

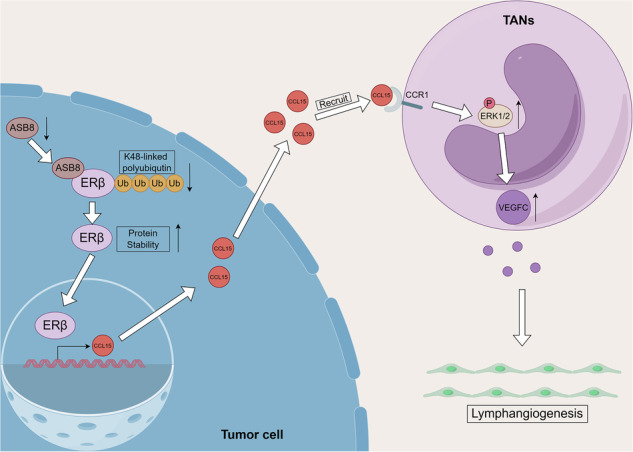

## Introduction

According to global cancer statistics, lung cancer is the leading cause of cancer incidence and mortality. By 2022, ~2.5 million new cases had been diagnosed worldwide, with over 1.8 million resulting deaths. The incidence of lung cancer in women is continually increasing, with adenocarcinoma being the most prevalent subtype [1, 2]. Despite advancements in various treatment modalities, including surgery, targeted therapy, and immunotherapy, recurrence and metastasis remain major challenges. Lymph node (LN) metastasis is the principal pathway for the spread of lung adenocarcinoma (LUAD), and clinical outcomes and prognosis largely depend on the extent of local LN involvement [3]. Tumor lymphangiogenesis facilitates lymphatic metastasis by enabling tumor cells to invade the LNs. Research indicates a positive correlation between the density of lymphatic vessels at primary LUAD sites and LN metastasis [4].

Estrogen receptor β (ERβ), a key component of the estrogen signaling pathway, plays a critical role in lung cancer progression, metastasis, and resistance to EGFR-TKI therapy [5–7]. ERβ can bind to genomic estrogen response elements and interact with transcription factors to regulate gene expression, additionally mediating cytoplasmic signaling via interactions with kinases in the PI3K/Akt and MAPK/ERK pathways [8]. Our prior research showed that ERβ protein expression is elevated in LN-metastatic LUAD compared to that in LN-negative cases. However, public database analyses, including the Cancer Genome Atlas (TCGA), showed no significant difference in ERβ mRNA levels between these groups, suggesting that the mechanisms driving the upregulation of ERβ protein and its role in promoting LN metastasis in LUAD require further investigation.

Within the tumor microenvironment, cancer cells, immune cells, and stromal cells secrete signaling molecules that stimulate lymphatic vessel formation and expansion. Studies have revealed that tumor cells and tumor-associated fibroblasts secrete VEGF-C and VEGF-D in response to various stimuli [9–11]. Similarly, tumor-associated macrophages, upon IL-1β stimulation, secrete substantial amounts of VEGF-C and VEGF-D [12]. In our previous analysis of TCGA-LUAD data, we observed that the activation of the estrogen signaling pathway increased the proportion of tumor-associated neutrophils (TANs) [13]. Recent studies suggest that TANs can secrete MMP9 and VEGFA, contributing to lymphatic vessel formation and LN metastasis in cancers such as bladder cancer [14]. However, whether neutrophils mediate ERβ-driven promotion of LN metastasis in LUAD remains to be elucidated.

In this study, we found that ERβ transcriptionally upregulates CCL15 expression in LUAD, which facilitates neutrophil infiltration via the CCR1 receptor. This infiltration subsequently enhances VEGFC secretion by neutrophils, thus promoting tumor lymphangiogenesis and LN metastasis in LUAD cells. Furthermore, we observed that reduced expression of the E3 ubiquitin ligase ASB8 in LN-metastatic LUAD lesions plays a critical role in maintaining the stability of the ERβ protein. These findings reveal a novel mechanism by which ERβ drives TAN-mediated lymphangiogenesis and LN metastasis.

## Method

### Antibodies and reagents

A comprehensive list of all the antibodies and reagents used in this study is provided in Supplementary Table 1.

### Clinical samples

All cancer tissue samples in this study were pathologically confirmed as LUAD between January 2013 and August 2024, with specimens having incomplete or ambiguous pathological or clinical data excluded. A total of 44 LUAD samples from non-small cell lung cancer tissue microarrays provided by Tongji Hospital were designated as Cohort 1 (Supplementary Table 2), while 63 formalin-fixed paraffin-embedded LUAD specimens from surgical patients at Union Hospital were classified as Cohort 2 (Supplementary Table 3). Each sample was independently verified by two pathologists. The study was approved by the Ethics Committee of Union Hospital, Tongji Medical College, Huazhong University of Science and Technology (Approval No. 2024-0457).

### Chemotaxis assay

Chemotaxis assays were conducted as previously described [15]. Inserts with 3 µm porous membranes (Corning) were placed in 24-well culture plates pre-coated with poly-HEMA to inhibit cell adhesion. A total of 200 µL of conditioned medium and 400 µL and RPMI-1640 medium were added to the lower chamber. Neutrophils (2 × 10^5^) were added to the upper chamber and incubated at 37 °C with 5% CO₂ for 30 min. After incubation, the number of migrated cells was quantified using a cell-counting board.

### HLEC tube formation assay

For the tube formation assay, a 48-well plate was precoated with Matrigel (356230, Corning). HLECs were seeded into coated wells and the appropriate conditioned medium was added. The plate was incubated at 37 °C in a 5% CO₂ atmosphere for 2 h, after which the lymphatic tube formation was observed using an inverted microscope. Tube lengths were quantified using ImageJ software.

### Cell apoptosis analysis

Cell apoptosis was evaluated using an apoptosis detection kit (KGA103, KeyGEN BioTECH) following the manufacturer’s protocol. After treatment, neutrophils were labeled with Annexin V-FITC and propidium iodide and analyzed by flow cytometry to determine the proportion of apoptotic cells.

### Neutrophil isolation

Neutrophils were isolated from heparinized peripheral blood using a human peripheral blood neutrophil isolation kit (LZS11131, TBD) according to the manufacturer’s instructions. The purity of the isolated neutrophils exceeded 98%, according to flow cytometry analysis (Supplementary Fig. 1D). Additionally, cell morphology was validated as neutrophils by Wright–Giemsa staining (Supplementary Fig. 1E). Isolated neutrophils were suspended in RPMI-1640 medium for subsequent experiments.

### Co-immunoprecipitation (Co-IP) and ubiquitination assays

For co-IP, cells were lysed in ice-cold IP buffer (G2038, Servicebio) supplemented with protease and phosphatase inhibitors. After centrifugation at 12,000 × *g* for 15 min, the lysates were incubated overnight at 4 °C with the designated antibody and protein A/G magnetic beads (HY-K0202, MCE). After washing with cold IP buffer, immune complexes were collected and analyzed by immunoblotting with the appropriate primary and secondary antibodies.

Ubiquitination analysis was conducted as previously described [16]. After transfection, the cells were lysed and the target proteins were immunoprecipitated using the same Co-IP protocol. Protein ubiquitination levels were assessed by immunoblotting using specific antibodies.

### Luciferase assay

Firefly luciferase activity was measured using the Dual-Luciferase Reporter Assay System (RG027, Beyotime) 48 h after transfection. Following the removal of the culture medium, 100 µL of lysis buffer was added to dissolve the cells for 15 min. Subsequently, Renilla luciferase detection buffer was added and mixed, and the relative light units (RLU) were recorded using a microplate reader (Varioskan LUX, Thermo Scientific). After the initial measurement, 100 µL of firefly luciferase assay reagent was added, and RLU was measured again. Renilla luciferase served as an internal control, and firefly luciferase RLU values were normalized to Renilla luciferase RLU to evaluate the reporter gene activity.

### Animal experiments

Female BALB/c nude mice (7 weeks old) were obtained from Beijing Vital River Laboratory Animal Technology Co., Ltd. H1975 cells (2 × 10^6^), infected with either the control oeRNA luciferase virus or the oeESR2 luciferase virus, were injected into the left lungs of the mice in a 50 µL mixture of PBS and Matrigel (1:1, BD Biosciences). Mice were treated with the corresponding drugs, and after 21 days, in vivo imaging was performed using a bioluminescence imaging system (PerkinElmer IVIS Spectrum Imaging System). Primary tumors and mediastinal LNs were harvested and single-cell suspensions were prepared for flow cytometric analysis to quantify specific neutrophil populations. All animal experiments were approved by the Animal Management and Use Committee of Huazhong University of Science and Technology (IACUC Number: 4123).

### Statistical analysis

All statistical analyses and graphs were generated using GraphPad Prism 10 software. Statistical differences between groups were determined using t-tests or one-way analysis of variance (ANOVA). Correlation analysis was performed using the Spearman’s method. Additional statistical analyses were conducted using R software (version 4.4.1). Statistical significance was set at *p* < 0.05. Prior to parametric tests, we verified variance homogeneity using Levene’s test (*α* = 0.05). Non-normal or heteroscedastic data were analyzed with the Kruskal–Wallis test. All experiments were performed in triplicate (*n* = 3) unless otherwise stated. Group variability was assessed by calculating SD for normally distributed data (confirmed by Shapiro-Wilk test, *P* > 0.05) or IQR for skewed distributions.

For other experimental methods, refer to the Supplementary Materials. All original Western blot data (full, uncropped membrane images) have been submitted as original Western blots.

## Results

### ERβ is associated with lymphatic vessel formation and lymph node metastasis in LUAD

**Fig. 1:**
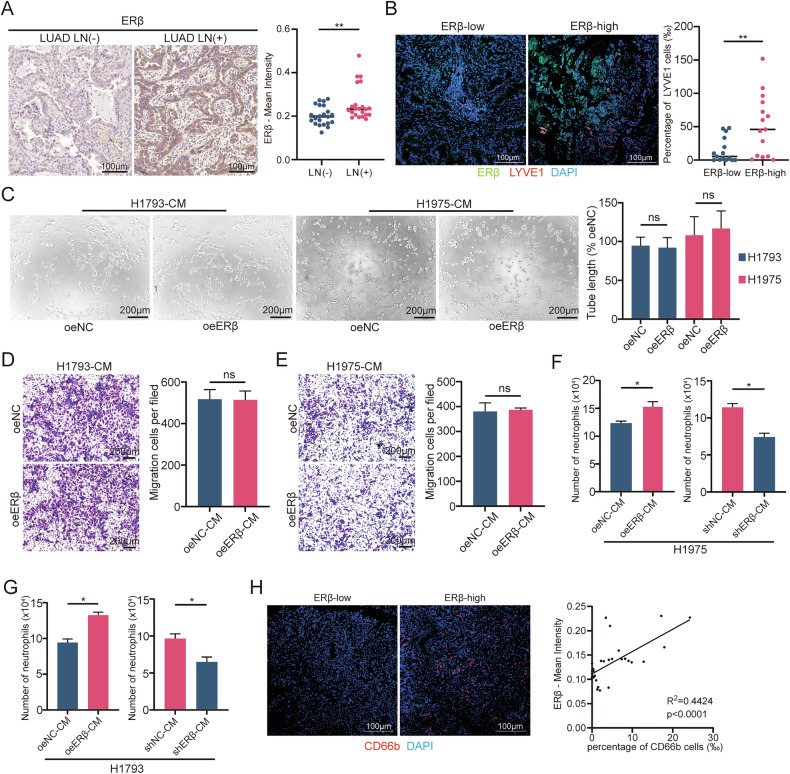
Association between ERβ and lymph node metastasis in LUAD and its positive correlation with TANs infiltration. **A** Representative IHC images of ERβ expression in LUAD (LN−) (*n* = 22) and (LN+) (*n* = 22) (left). Quantification of ERβ expression in the two groups (right). Scale bar: 100 μm. **B** Representative immunofluorescence images of ERβ and LYVE1 in LUAD tissues (*n* = 30) (left) and quantification of LYVE1^+^ cells (right). Scale bar: 100 μm. **C** Representative images of tube formation assays in HLECs incubated with (CM) from H1793 and H1975 cells for 2 h (left), with histograms showing the percentage of tube length in the experimental versus the control groups (right). Scale bar: 200 μm. **D**, **E** Effect of conditioned media from H1793 and H1975 cells (oeNC, oeERβ) on HLEC migration. Scale bar: 200 μm. **F**, **G** Effect of conditioned medium from H1793 and H1975 cells (oeNC, oeERβ, shNC, and shERβ) on neutrophil chemotaxis. **H** Representative immunofluorescence images of CD66b in LUAD tissues (*n* = 30) (left) and correlation analysis of ERβ expression with the proportion of CD66^+^ cell infiltration (*r*² (Spearman) = 0.4424; *p* < 0.0001) (right). Statistical significance was evaluated using a two-tailed t-test. * *p* < 0.05, ** *p* < 0.01, *** *p* < 0.001, **** *p* < 0.0001.

To explore ERβ‘s role in LN metastasis in LUAD, we analyzed 44 ERβ-stained LUAD cases, including 22 LN-negative and 22 LN-metastatic patients. ERβ expression was higher in LN-metastatic tissues (Fig. 1A). In 30 additional LUAD samples, high ERβ expression correlated with increased microlymphatic density (MLD) based on LYVE1 staining (Fig. 1B). To assess ERβ‘s direct impact on lymphangiogenesis, we performed an in vitro tube formation assay using HLECs and established H1975 and H1793 cell lines with ERβ overexpression and silencing, respectively (Supplementary Fig. 1C). No differences in tube formation or Transwell assay results were observed between oeNC and oeERβ groups in tumor cell-conditioned medium (CM) (Fig. 1C–E).

### ERβ positively regulates neutrophil chemotaxis

The tumor microenvironment’s complexity involves various cell types, with ERβ potentially promoting LN metastasis through cell interactions. Using TCGA-LUAD transcriptomic data, we employed the CIBERSORT algorithm to compare immune cell infiltration between LN-negative and LN-metastatic samples, focusing on memory B cells, gamma-delta T cells, M0 macrophages, and neutrophils (Supplementary Fig. 1A). Immunohistochemical staining for CD27 (memory B cells), CD3 delta (gamma delta T cells), and CD68 (M0 macrophages) in 20 LUAD samples showed no significant differences in these cell types between high and low ERβ expression groups (Supplementary Fig. 1B). However, immunofluorescence staining for CD66b in 30 paraffin-embedded samples indicated a positive correlation between CD66b+ neutrophil infiltration and ERβ expression (Fig. 1H).

To explore ERβ's role in neutrophils in LUAD, neutrophils were isolated from healthy individuals' blood. Chemotaxis assays showed that tumor cell CM overexpressing ERβ attracted more neutrophils, while ERβ knockdown reduced this effect (Fig. 1F and G), indicating ERβ may regulate neutrophil-targeting chemotactic factors. Additionally, ERβ inhibited neutrophil apoptosis after 12 h in oeERβ tumor cell CM (Supplementary Fig. 2). In situ lung cancer mouse model using oeERβ-H1975 cells, immunohistochemical staining for Ly6G revealed increased neutrophil infiltration in the oeERβ group compared to controls (Supplementary Fig. 1F).

### ERβ promotes lymphangiogenesis and lymph node metastasis in LUAD through TANs

**Fig. 2:**
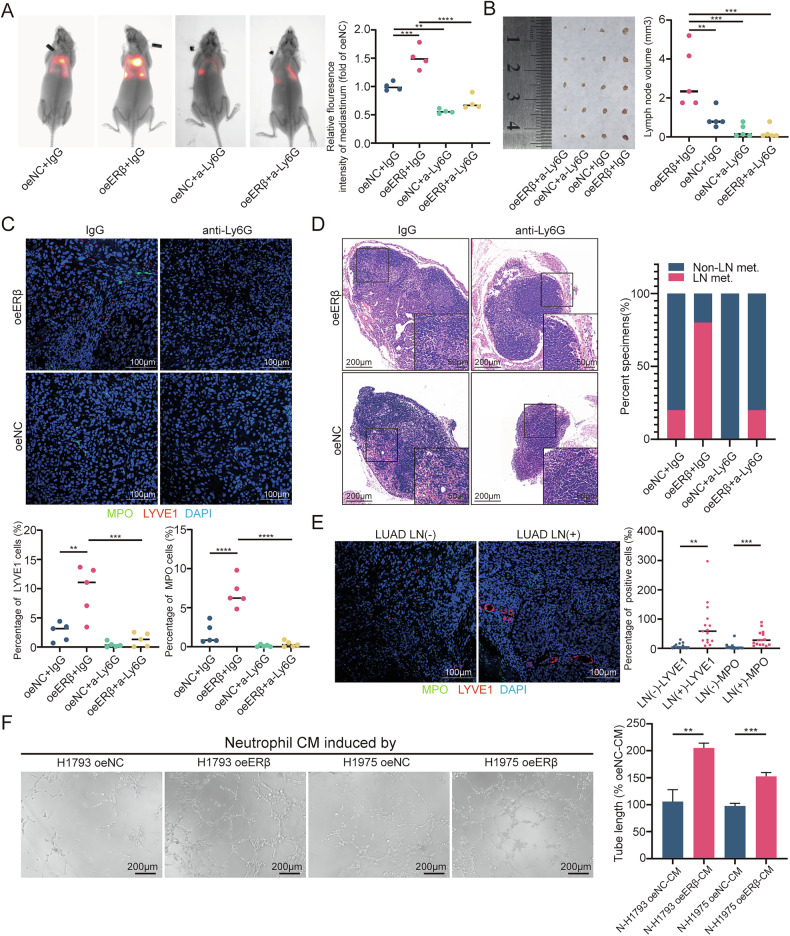
ERβ promotes lymphangiogenesis and LN metastasis in lung adenocarcinoma through TANs. **A** Representative bioluminescence images (left) and histogram analysis (right) of mediastinal metastasis in the indicated nude mice. Relative luminescence is normalized to 1 in the empty vector (oeNC) group. **B** Images of mediastinal LNs from nude mice injected with oeNC or oeERβ H1975 cells for 3 weeks, with or without anti-Ly6G treatment (left). Analysis of LN volume (right). **C** Representative immunofluorescence images (top) showing MPO and LYVE1 staining, with quantification of MPO^+^ and LYVE1^+^ cells (bottom) in left lung primary tumors from the indicated nude mice. Scale bar: 100 μm. **D** Representative H&E images (left) and quantification of LN metastasis status (right) in the indicated lymph nodes. Scale bars: 200 μm and 50 μm. **E** Representative immunofluorescence images of MPO and LYVE1 in LUAD (LN−) (*n* = 15) and LUAD (LN+) (*n* = 15) tissues (left), with quantification of MPO^+^ and LYVE1^+^ cells (right). Scale bar: 100 μm. **F** Representative images of tube formation assays in HLECs incubated for 2 h with CM from neutrophils stimulated by CM from the specified H1793 and H1975 cells (left). Histograms show the percentage of tube length in experimental versus control groups (right). Scale bar: 200 μm. Statistical significance was assessed using two-tailed t-tests or one-way ANOVA. *p < 0.05, **p < 0.01, ***p < 0.001.

To assess whether ERβ promotes lymphangiogenesis and LN metastasis in LUAD via neutrophils, we depleted neutrophils in mice using an anti-Ly6G antibody before tumor cell inoculation and continued treatment until significant mediastinal metastasis was detected (Supplementary Fig. 3A and B). Starting from day 1 post-inoculation, anti-Ly6G antibody was administered via intraperitoneal injection at an initial dose of 400 μg per mouse, followed by a maintenance dose of 100 μg per mouse every 4 days. Flow cytometry confirmed effective neutrophil depletion in peripheral blood and tumors (Supplementary Fig. 3C, gating strategy in Supplementary Fig. 3D). In vivo imaging showed that neutrophil depletion reversed ERβ-induced metastasis in nude mice (Fig. 2A). The oeERβ group exhibited larger mediastinal LNs and higher metastasis rates, both reduced by neutrophil depletion (Fig. 2B and D). Immunofluorescence staining for MPO and LYVE1 in paraffin-embedded lung tumor sections further confirmed increased MLD in the oeERβ group, which was reversed by neutrophil depletion (Fig. 2C).

To validate these findings, 15 LN-negative and 15 LN-metastatic LUAD specimens were analyzed. Immunofluorescence staining showed higher MPO+ neutrophil infiltration and MLD in LN-metastatic tissues (Fig. 2E). Tube formation assays using HLECs treated with tumor CM revealed that neutrophil CM, stimulated by oeERβ H1793 or H1975 cell CM, enhanced tube formation (Fig. 2F). These results suggest that ERβ promotes lymphangiogenesis and LN metastasis in LUAD via neutrophils.

### ERβ recruits neutrophils via the CCL15-CCR1 axis

**Fig. 3:**
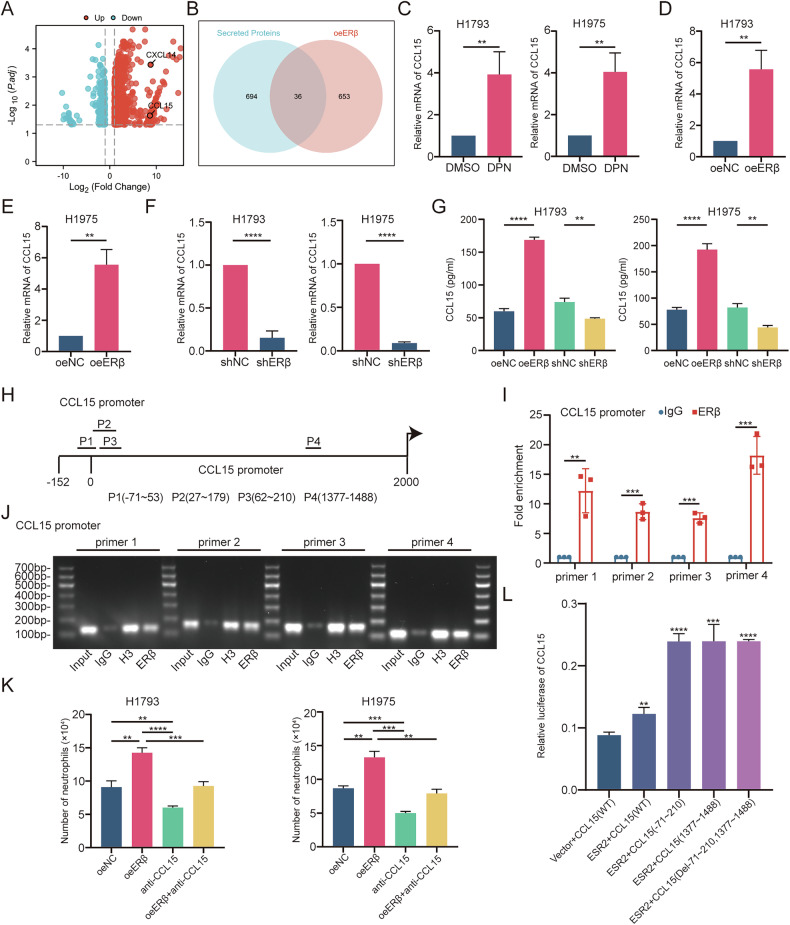
ERβ transcriptionally upregulates CCL15 expression in LUAD. **A** Volcano plot of gene expression changes in oeERβ-H1975 cells, with upregulated genes shown in red and downregulated genes in blue. **B** Venn diagram showing the overlap between ERβ-upregulated genes and secreted proteins. **C** RT-qPCR analysis of *CCL15* mRNA in LUAD cells treated with DPN. **D**–**F** RT-qPCR analysis of *CCL15* mRNA in LUAD cells with shERβ and oeERβ manipulation. **G** ELISA analysis of CCL15 in the supernatants of shERβ and oeERβ LUAD cells. **H** Schematic representation of the ChIP primer design from −2000 to 0 bp around the transcription start site (TSS). **I** ESR2 levels at the ASB8 promoter normalized to input. **J** Representative images from gel electrophoresis experiments. **K** Luciferase activity in DPN-treated HEK293T cells transfected with ERβ, CCL15 promoter, and Renilla luciferase reporter (pRL-TK) plasmids, as measured in a dual-luciferase reporter assay. **L** Neutrophil chemotaxis induced by conditioned media (CM) from oeERβ LUAD cells after CCL15 neutralizing antibody treatment. Statistical significance was assessed using two-tailed t-tests or one-way ANOVA. *p < 0.05, **p < 0.01, ***p < 0.001.

To investigate how ERβ recruits neutrophils, transcriptome sequencing in oeERβ-H1975 cells identified CXCL14 and CCL15 as potential candidates by intersecting upregulated genes with known secreted proteins [17] (Fig. 3A, B). Given CCL15’s role in neutrophil recruitment [18], it was selected for further study. RT-qPCR and ELISA confirmed ERβ-induced CCL15 upregulation (Fig. 3C–G). ChIP-qPCR demonstrated ERβ binding to the CCL15 promoter (Fig. 3H–J, primer sequences in Supplementary Table 6). Luciferase assays using truncated CCL15 promoter plasmids revealed ERβ activation, with higher activity in specific truncations, suggesting inhibitory regions (Fig. 3L). A chemotaxis assay showed that a CCL15-neutralizing antibody blocked ERβ-mediated neutrophil recruitment (Fig. 3K).

**Fig. 4:**
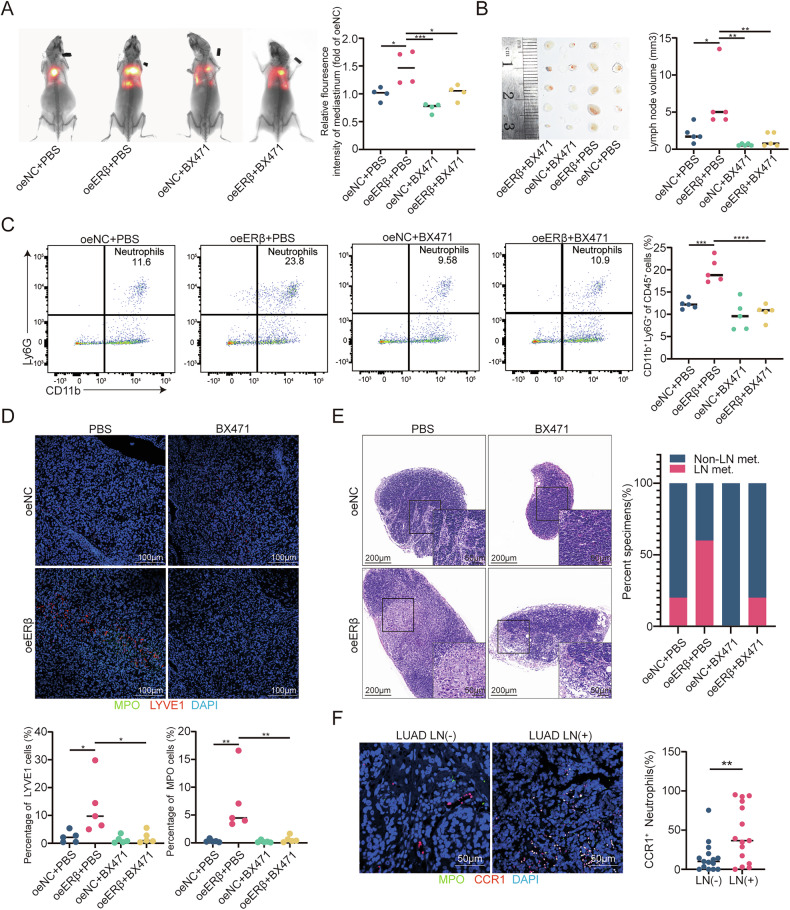
ERβ recruits neutrophils via the CCL15-CCR1 axis. **A** Representative bioluminescence images (left) and histogram analysis (right) of mediastinal metastasis in the indicated nude mice. Relative luminescence is normalized to 1 in the empty vector (oeNC) group. **B** Images of mediastinal LNs (left) from nude mice injected with oeNC or oeERβ H1975 cells for 3 weeks, with or without BX471 treatment. Analysis of LN volume (right). **C** Flow cytometry analysis of CD45^+^CD11b^+^Ly6G^+^ neutrophils from freshly excised primary tumor tissues in the left lung. **D** Representative immunofluorescence images (top) showing MPO and LYVE1 staining, with quantification of MPO^+^ and LYVE1^+^ cells (bottom) infiltrated in primary tumor tissues of the left lung in the indicated nude mice. Scale bar: 100 μm. **E** Representative H&E images (left) and quantification of LN metastasis status (right) in the indicated LNs. Scale bars: 200 μm and 50 μm. **F** Representative immunofluorescence images of MPO and LYVE1 in LUAD (LN−) (*n* = 15) and LUAD (LN+) (*n* = 15) tissues (left), with quantification of MPO^+^ and LYVE1^+^ cells (right). Scale bar: 100 μm. Statistical significance was assessed using two-tailed t-tests or one-way ANOVA. *p < 0.05, **p < 0.01, ***p < 0.001.

An in situ lung cancer model was established by injecting oeNC and oeERβ H1975 cells into nude mice. Mice received BX471 (20 mg/kg, once daily, subcutaneously) for 21 days. In vivo imaging showed that BX471 suppressed ERβ-induced mediastinal metastasis (Fig. 4A). The oeERβ + BX471 group had smaller mediastinal LNs and a lower metastasis rate than the oeERβ group (Fig. 4B, E). Flow cytometry revealed fewer Ly6G + CD11b+ neutrophils in lung tumors from the oeERβ + BX471 group (Fig. 4C). Immunofluorescence staining confirmed increased MLD in the oeERβ group, which was reversed by BX471 (Fig. 4D). Additionally, staining for CCR1 and MPO in 30 LUAD specimens showed more MPO + CCR1+ neutrophils in LN-metastatic tissues than in LN-negative tissues (Fig. 4F).

### VEGFC derived from neutrophils is critical for lymphangiogenesis

**Fig. 5:**
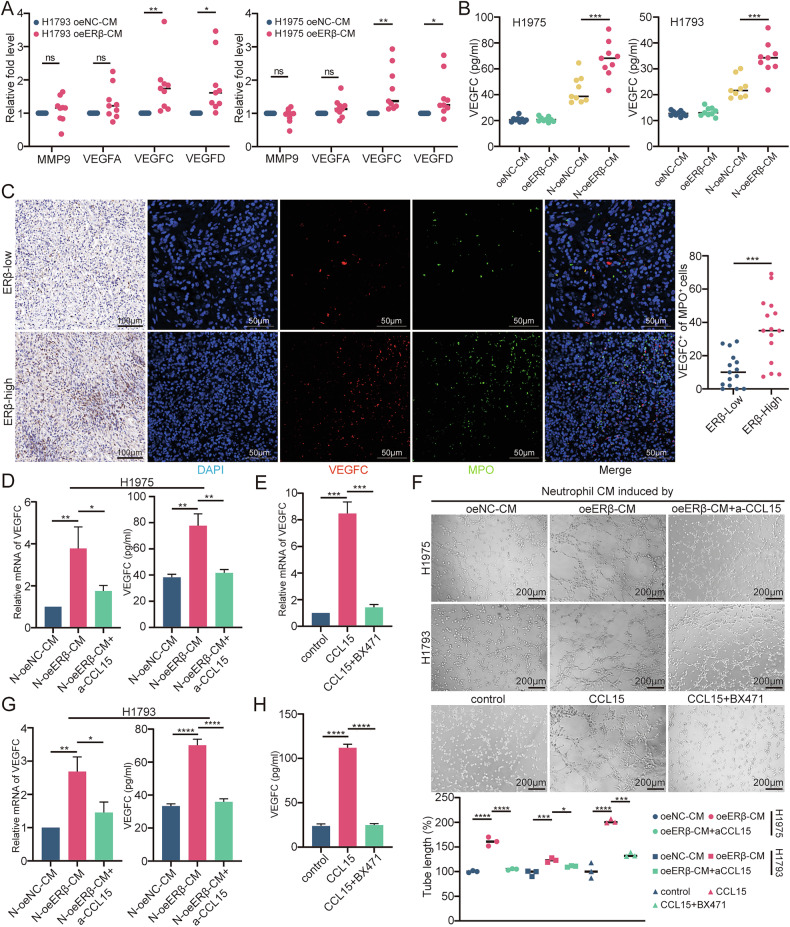
LUAD cell-derived CXCL15 promotes TANs-modulated lymphangiogenesis. **A** RT-qPCR analysis of *VEGFA*, *VEGFC*, *VEGFD*, and *MMP9* mRNA in neutrophils stimulated for 3 h with CM from the specified H1793 and H1975 cells. **B** ELISA analysis of VEGFC in neutrophils stimulated for 12 h with CM from the specified H1793 and H1975 cells. **C** Representative immunofluorescence images showing VEGFC and MPO staining in ERβ-Low (*n* = 15) and ERβ-High (*n* = 15) human LUAD tissues (left), with quantification of MPO^+^ and VEGFC^+^ cells (right). Scale bars: 100 μm and 50 μm. **D** RT-qPCR analysis of VEGFC mRNA in neutrophils incubated with CM from the specified H1975 cells and treated with a CCL15 neutralizing antibody for 3 h, along with ELISA analysis of VEGFC in neutrophils incubated with the same CM and treated with the CCL15 neutralizing antibody for 12 h. **E** RT-qPCR analysis of VEGFC mRNA in neutrophils stimulated with CCL15 recombinant protein and the CCR1 inhibitor BX471 for 3 h. **F** Representative images of tube formation assays in HLECs incubated for 2 h with neutrophil CM, following stimulation by CM from the specified H1793 and H1975 cells with added CCL15 neutralizing antibody or by neutrophil CM with CCL15 recombinant protein and BX471 (top). Histograms show the percentage of tube length in experimental versus control groups (bottom). Scale bar: 200 μm. **G** RT-qPCR analysis of VEGFC mRNA in neutrophils incubated with CM from the specified H1793 cells and treated with a CCL15 neutralizing antibody for 3 h, along with ELISA analysis of VEGFC in neutrophils incubated with the same CM and treated with the CCL15 neutralizing antibody for 12 h. **H** ELISA analysis of VEGFC in neutrophil CM after 12 h of stimulation with CCL15 recombinant protein and CCR1 inhibitor BX471. Statistical significance was assessed using two-tailed t-tests or one-way ANOVA. *p < 0.05, **p < 0.01, ***p < 0.001.

Previous studies have shown that MMP9, VEGFA, VEGFC, and VEGFD promote lymphatic vessel formation [14]. To investigate how TANs contribute to lymphangiogenesis, we analyzed gene expression in neutrophils stimulated with CM from H1793 and H1975 cells. RT-qPCR showed that VEGFC and VEGFD were upregulated in neutrophils treated with oeERβ-CM (Fig. 5A). Since VEGFC is key for lymphatic tube formation [4], we focused on VEGFC. ELISA confirmed that VEGFC production was higher in neutrophils stimulated by oeERβ-CM than in those treated with oeNC-CM, and exceeded levels produced by tumor cells alone (Fig. 5B). Immunofluorescence staining for MPO and VEGFC in LUAD tissues further revealed a higher proportion of VEGFC + MPO+ cells in tissues with elevated ERβ expression (Fig. 5C). These findings suggest that neutrophil-derived VEGFC is crucial for HLEC tube formation and lymphangiogenesis in LUAD.

### The CCL15-CCR1 axis is crucial for neutrophil-mediated lymphangiogenesis

CCR1 activation has been reported to upregulate ERK phosphorylation, promoting VEGFC expression via the MEK1/2-ERK pathway [19–21]. We hypothesized that LUAD-derived CCL15 interacts with CCR1 on neutrophils to enhance ERK phosphorylation, thereby increasing VEGFC production. To test this, we measured VEGFC levels in neutrophils stimulated with oeERβ-CM, with or without a CCL15-neutralizing antibody, which significantly reduced VEGFC expression (Fig. 5D, G). Further, recombinant CCL15 increased VEGFC expression, an effect inhibited by the CCR1 inhibitor BX471 (Fig. 5E, H). Western blot confirmed that oeERβ-CM and recombinant CCL15 induced ERK1/2 phosphorylation in neutrophils, which was reversed by the CCL15-neutralizing antibody and BX471 (Supplementary Fig. 4A).

To explore this pathway, neutrophils were treated with the ERK1/2 agonist Ro 67-7476 and the inhibitor PD98059. Ro 67-7476 increased ERK1/2 phosphorylation and VEGFC production, while PD98059 reversed these effects (Supplementary Fig. 4B, C). CCL15 neutralization and CCR1 inhibition impaired HLEC tube formation induced by oeERβ-CM or CCL15-stimulated neutrophils (Fig. 5F). PD98059 also reduced HLEC tube formation induced by Ro 67-7476-stimulated neutrophils (Supplementary Fig. 4D). These findings support the critical role of the CCL15-CCR1-ERK-VEGFC axis in neutrophil-mediated lymphangiogenesis in LUAD.

### Identification of ERβ regulators

To investigate the relationship between ERβ and LN metastasis in LUAD, we analyzed four transcriptomic datasets using the “BEST” web tool (https://rookieutopia.com/app_direct/BEST/) [22]. ESR2 levels were not elevated in LN metastatic tissues compared to LN-negative LUAD tissues (Supplementary Fig. 5A), suggesting that other proteins may influence ERβ stability, leading to its higher expression in metastatic tissues.

LC-MS/MS analysis of HEK293T cells transfected with Flag-ESR2 plasmids identified two ERβ-binding E3 ubiquitin ligases: ASB8 and HERC2. Given ubiquitination’s role in cancer progression [23], we explored E3 ligases regulating ERβ during LUAD metastasis.

**Fig. 6:**
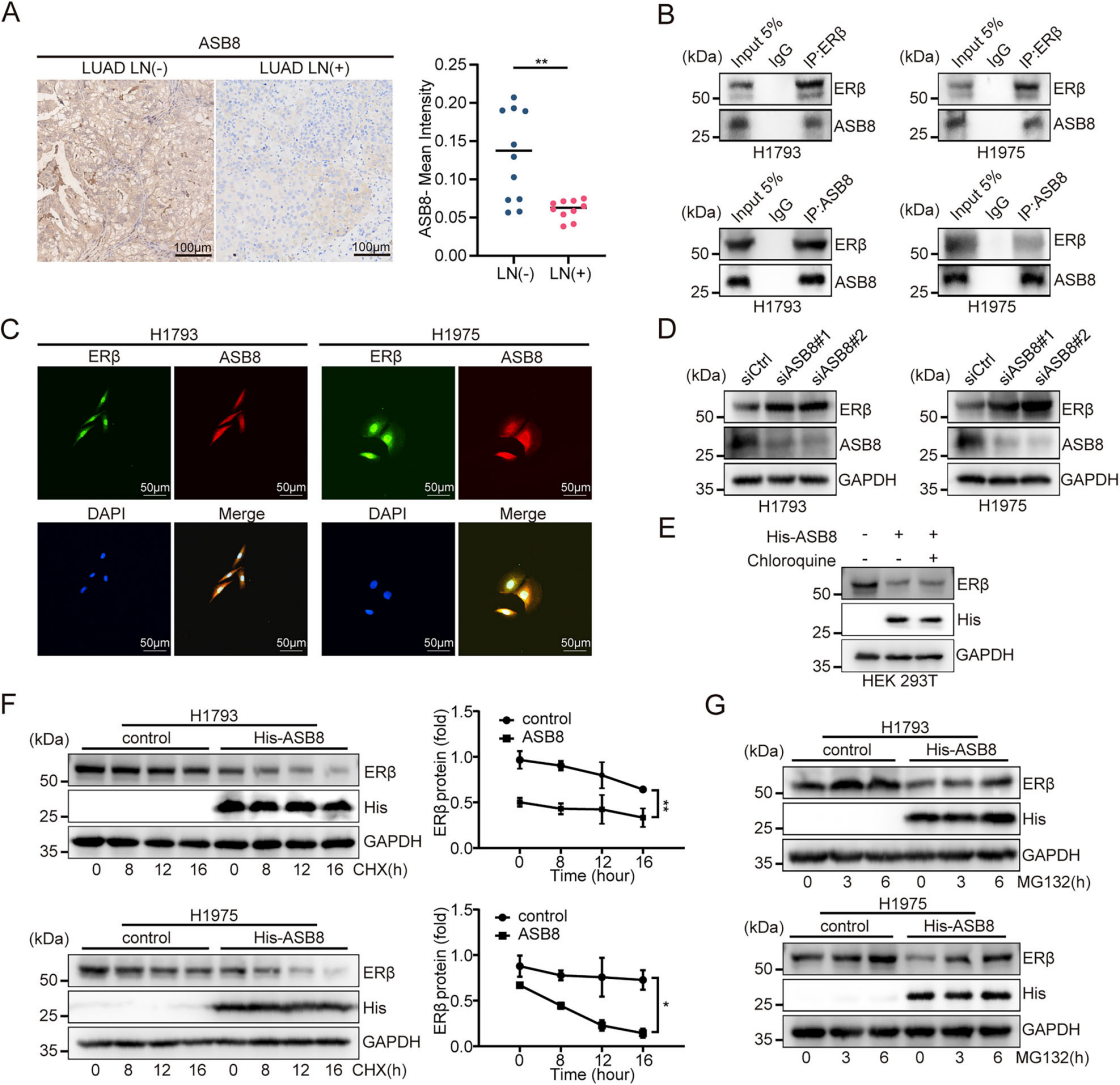
Identification of effective inhibitors of ERβ. **A** Representative IHC images of ASB8 in LUAD (LN−) (*n* = 10) and LUAD (LN+) (*n* = 10) tissues (left), with quantification of ASB8 expression between the two groups (right). Scale bar: 100 μm. **B** Endogenous interaction between ERβ and ASB8. H1793 and H1975 cells were immunoprecipitated (IP) with antibodies against ERβ or ASB8, followed by immunoblotting (IB) analysis. The input is equivalent to 5% of the cell lysates used for immunoprecipitation. **C** Immunofluorescence staining of ERβ (green), ASB8 (red), and DAPI (blue) in H1793 and H1975 cells. Scale bar: 50 μm. **D** Western blot analysis of ERβ and ASB8 in LUAD cells transfected with ASB8 siRNA. **E** Western blot analysis of ERβ protein levels in 293 T cells transfected with ASB8 expression vector and treated with 50 μM chloroquine for 24 h. **F** ERβ protein levels in empty vector and ASB8-overexpressing H1793 or H1975 cells after incubation with CHX (20 μg/ml) at indicated time points. **G** Western blot showing the effect of MG132 (10 μM) on ERβ expression levels in LUAD cells with or without ASB8 overexpression. Statistical significance was assessed using two-tailed t-tests or one-way ANOVA. *p < 0.05, **p < 0.01, ***p < 0.001.

Immunohistochemistry showed lower ASB8 expression in LN metastatic LUAD tissues (Fig. 6A, Supplementary Fig. 5B). ASB8-ERβ interactions were confirmed endogenously and exogenously (Fig. 6B, Supplementary Fig. 5C and D), with colocalization observed in H1975 and H1793 cells (Fig. 6C). ASB8 knockdown increased ERβ protein levels without affecting ESR2 gene expression (Fig. 6D, Supplementary Fig. 5E), suggesting ASB8 regulates ERβ stability. To investigate the potential involvement of the lysosomal pathway in ASB8-mediated ERβ degradation, we performed chloroquine inhibition assays in 293 T cells overexpressing ASB8. Notably, chloroquine treatment failed to restore ERβ protein levels (Fig. 6E), demonstrating that ASB8-induced ERβ degradation is lysosome-independent. Further mechanistic studies revealed that ASB8 overexpression significantly shortened ERβ protein half-life in cycloheximide chase experiments (Fig. 6F), and this effect was partially reversed by proteasome inhibition with MG132 (Fig. 6G). Together, these results provide compelling evidence that ASB8 specifically targets ERβ for proteasomal degradation rather than lysosomal degradation, establishing a clear post-translational regulatory mechanism for their observed inverse correlation in LUAD.

### ASB8 induces K48-linked polyubiquitination of ERβ

**Fig. 7:**
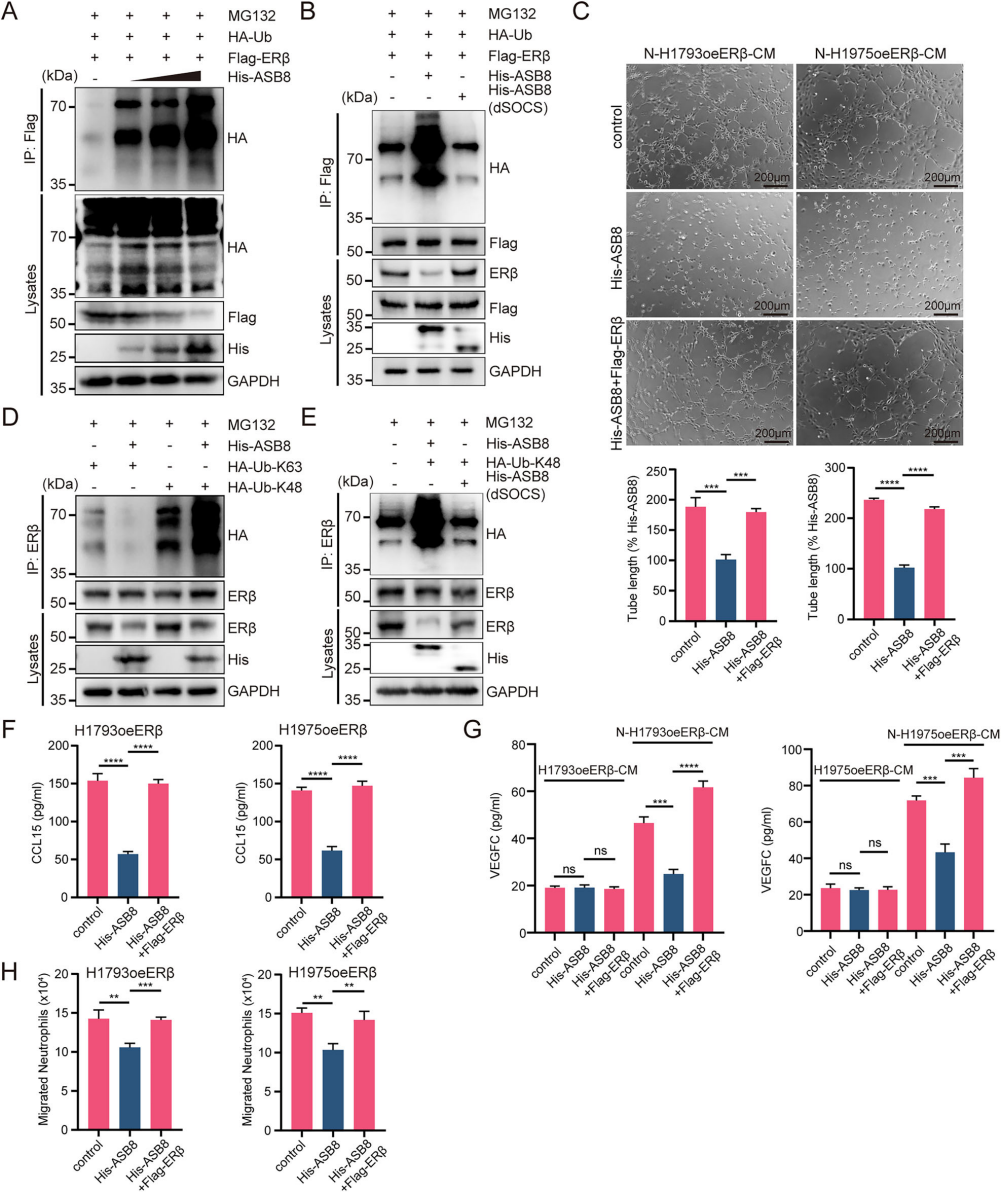
ASB8 Induces K48-Linked Polyubiquitination of ERβ, Inhibiting CCL15 and VEGFC Secretion and Lymphangiogenesis. **A** Immunoprecipitation with anti-Flag beads using lysates from HEK293T cells transfected with plasmids expressing Flag-ERβ, HA-Ub, and increasing amounts of His-ASB8, followed by immunoblotting with anti-HA antibody. **B** Immunoblot showing ubiquitination of ERβ in HEK293T cells transfected with specified plasmids. dSOCS indicates deletion of the SOCS domain. **C** Representative images from tube formation assays in HLECs incubated for 2 h with neutrophil-CM stimulated by CM from H1793 and H1975 cells transfected with Flag-ERβ and His-ASB8 (top), with histograms showing the percentage of tube length in experimental versus control groups (bottom). Scale bar: 200 μm. **D** Western blot analysis showing K48-linked ubiquitination of ERβ in HEK293T cells co-transfected with His-ASB8 and HA-Ub-K48 or HA-Ub-K63. **E** Western blot analysis showing K48-linked ubiquitination of ERβ in HEK293T cells co-transfected with His-ASB8, His-ASB8(dSOCS), and HA-Ub-K48. **F** ELISA analysis of CCL15 in CM from H1793 and H1975 cells transfected with Flag-ERβ and His-ASB8. **G** ELISA analysis of VEGFC in the supernatant of neutrophils stimulated for 12 h with CM from H1793 and H1975 cells transfected with Flag-ERβ and His-ASB8. **H** Neutrophil chemotaxis induced by CM from H1793 and H1975 cells transfected with Flag-ERβ and His-ASB8. Statistical significance was assessed using two-tailed t-tests or one-way ANOVA. *p < 0.05, **p < 0.01, ***p < 0.001.

To confirm ASB8’s role in ERβ ubiquitination, we performed ubiquitination assays in HEK293T cells. ASB8 enhanced ERβ polyubiquitination in a dose-dependent manner (Fig. 7A), but deletion of its SOCS domain (dSOCS) abolished this effect (Fig. 7B), highlighting the SOCS domain’s critical role in ASB8-mediated ubiquitination.

Further analysis showed that ASB8 primarily induced K48-linked polyubiquitination of ERβ, as evidenced by increased K48-linked ubiquitin chains in ASB8-overexpressing cells (Fig. 7D). This effect was significantly reduced upon SOCS domain deletion (Fig. 7E), confirming that ASB8 promotes ERβ degradation via K48-linked polyubiquitination.

### ASB8 overexpression disrupts ERβ stability in LUAD cells, inhibiting CCL15 and VEGFC secretion and lymphangiogenesis

To examine the effects of ASB8 on the ERβ-CCL15-VEGFC axis and lymphangiogenesis, we overexpressed ASB8 in LUAD cells with stable ERβ overexpression. ASB8 overexpression reduced lymphangiogenesis promoted by oeERβ cells through neutrophils; this effect was reversed upon reintroduction of exogenous ERβ (Fig. 7C). ELISA results showed that CCL15 levels in oeERβ-CM and VEGFC levels in neutrophil supernatants were significantly lower after ASB8 overexpression but were restored with reintroduced ERβ (Fig. 7F, G). Furthermore, neutrophil chemotactic ability was notably reduced in ASB8-overexpressing oeERβ-CM but was restored by reintroducing exogenous ERβ (Fig. 7I). These findings indicated that ASB8 disrupts the ERβ-CCL15-VEGFC pathway and inhibits neutrophil-mediated lymphangiogenesis.

### Pharmacological inhibition of ERβ attenuates neutrophil recruitment and lymphatic metastasis in vivo

**Fig. 8:**
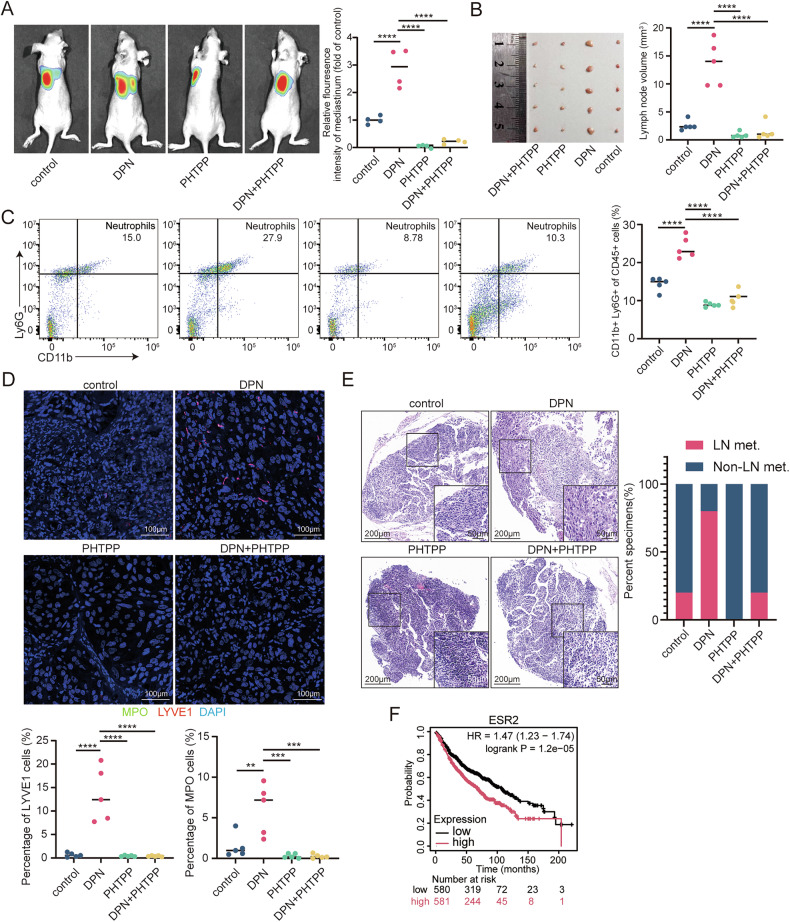
Pharmacological inhibition of ERβ attenuates neutrophil recruitment and lymphatic metastasis in vivo. **A** Representative bioluminescence images (left) and histogram analysis (right) of mediastinal metastasis in the indicated nude mice. Relative luminescence is normalized to 1 in the empty vector (control) group. **B** Images of mediastinal LNs (left) from nude mice injected with H1975 cells for 3 weeks, with or without DPN (5 nmol per mouse) and PHTPP (33 nmol per mouse) treatment. Analysis of LN volume (right). **C** Flow cytometry analysis of CD45^+^CD11b^+^Ly6G^+^ neutrophils from freshly excised primary tumor tissues in the left lung. **D** Representative immunofluorescence images (top) showing MPO and LYVE1 staining, with quantification of MPO^+^ and LYVE1^+^ cells (bottom) infiltrated in primary tumor tissues of the left lung in the indicated nude mice. Scale bar: 100 μm. **E** Representative H&E images (left) and quantification of LN metastasis status (right) in the indicated LNs. Scale bars: 200 μm and 50 μm. **F** High ESR2 expression correlates with poorer LUAD survival (KMPlot; HR = 1.47, *p* = 1.2e-5). Statistical significance was assessed using two-tailed t-tests or one-way ANOVA. *p < 0.05, **p < 0.01, ***p < 0.001.

Pharmacological modulation of ERβ signaling revealed its pivotal role in neutrophil-mediated metastasis. In our orthotopic lung cancer model, ERβ agonist diarylpropionitrile (DPN) significantly promoted mediastinal metastasis as evidenced by in vivo imaging (Fig. 8A) and increased mediastinal lymph node size (Fig. 8B, E), whereas ERβ antagonist 4-[2-Phenyl-5,7-bis (trifluoromethyl) pyrazolo [1,5-a]-pyrimidin-3-yl] phenol (PHTPP) effectively counteracted these effects. Mechanistically, PHTPP treatment reduced tumor-associated Ly6G + CD11b+ neutrophils compared to DPN-treated mice (Fig. 4C), concurrently normalizing MLD (Fig. 4D). Notably, the DPN + PHTPP combination group exhibited intermediate phenotypes, confirming dose-dependent ERβ regulation of the neutrophil-lymphatic axis. These findings not only corroborate our CCR1 inhibition data but crucially establish that direct ERβ blockade achieves comparable anti-metastatic effects through neutrophil suppression, highlighting ERβ as a druggable target for intercepting metastatic cascades in LUAD.

Clinically, bioinformatics analysis of multicenter cohorts (Kaplan-Meier Plotter database) established ESR2 as a significant prognostic biomarker in LUAD, with elevated expression predicting poorer overall survival (HR = 1.47, *p* = 1.2e-5; Fig. 8F). This clinical correlation reinforces our experimental findings that ERβ promotes metastatic progression.

## Discussion

In this study, we demonstrated that ERβ promotes LN metastasis of LUAD cells by regulating lymphangiogenesis through TANs, and identified the E3 ubiquitin ligase ASB8 as a key molecule in maintaining ERβ stability. Specifically, ERβ directly enhances CCL15 transcription, thereby recruiting neutrophils via CCR1. It also increases ERK1/2 phosphorylation in neutrophils, boosting VEGFC expression, which promotes tumor lymphangiogenesis and facilitates LN metastasis. ASB8, an E3 ubiquitin ligase for ERβ, is expressed at low levels in LN-metastatic LUAD, leading to increased ERβ protein stability.

Previous investigations have identified ERβ as a key factor in the progression and metastasis of non-small cell lung cancer. However, these studies predominantly concentrated on tumor cells, often neglecting changes occurring in other components of the tumor microenvironment [24–26]. Notably, our previous research revealed that ERβ expression is elevated in metastatic LNs of LUAD compared with the corresponding primary lesions, indicating a potential association between high ERβ levels and LN metastasis [5]. However, our experimental findings suggest that LUAD cells with elevated ERβ expression do not directly drive lymphangiogenesis, suggesting that ERβ may indirectly influence lymphangiogenesis via other microenvironmental cells.

Emerging evidence highlights the critical involvement of neutrophils in tumor metastasis [27–30]. For example, bladder tumor cells secrete CXCL1 and CXCL8, which recruit neutrophils and activate the ERK and JNK signaling pathways. This, in turn, triggers VEGFA and MMP9 expression, facilitating tumor-induced lymphangiogenesis and LN metastasis [14]. Similarly, in breast tissue, estrogen promotes neutrophil recruitment through the CXCR2 pathway [31] and enhances N2 polarization by upregulating LFA-1 expression, ultimately driving breast cancer metastasis [32]. In this study, using clinical samples, cell models, and animal experiments, we demonstrated that ERβ facilitates LN metastasis in LUAD via the CCL15-CCR1 axis by recruiting neutrophils. This finding further expands our understanding of how estrogen receptor-mediated mechanisms contribute to LUAD metastasis.

It is important to note that most existing animal models for investigating LN metastasis rely on the mouse popliteal LN metastasis model [14, 33, 34]. However, this model has limitations in accurately mimicking the metastatic behavior of tumor cells originating in lung tissue. In this study, we used an orthotopic lung cancer model in nude mice with mediastinal LN metastases. This model more closely replicates the clinical characteristics of lung cancer and offers a superior platform for exploring the mechanisms underlying lung cancer metastasis [35, 36]. Additionally, metastasis to organs such as the brain and liver was not observed, possibly because of the cell line used, indicating the need for further investigation into the applicability of this model for lung cancer metastasis research.

Previous studies have primarily assessed ERβ expression in lung cancer using immunohistochemical staining of tissue specimens without focusing on the mRNA expression levels of ERβ in publicly available datasets. Through public LUAD database analysis and experimental validation, we discovered that ERβ protein expression differs between LN-negative and LN-metastatic cases and is regulated by E3 ubiquitin ligase ASB8. ASB8, a poorly characterized substrate recognition unit in a subset of E3 ubiquitin ligase complexes, has few known physiological targets [37]. Research on ASB8 in tumors is rare, with only one study indicating its potential role in promoting lung cancer growth and proliferation [38]. Our study demonstrated that ASB8 degrades the ERβ protein through K48-linked ubiquitination; however, its low expression in LN-metastatic LUAD leads to increased ERβ stability, promoting LN metastasis. Further research is required to clarify whether ASB8 directly or indirectly interacts with ERβ, and to identify potential binding sites. The specific role of ASB8 in LUAD warrants further investigation.

While our study highlights ASB8-mediated ubiquitination as a central regulator of ERβ stability, we recognize that ERβ homeostasis likely involves more complex, multilayered mechanisms. Our group recently identified USP7 as another regulator of ERβ stability in osimertinib-resistant NSCLC cells [6], suggesting coordinated control by both E3 ligases (e.g., SCF complex components or VHL) and deubiquitinases (e.g., USP family members) in LUAD. Beyond ubiquitination, other post-translational modifications may fine-tune ERβ stability - phosphorylation (potentially via GSK-3) and SUMOylation could either compete with ubiquitination sites or recruit specialized degradation machinery (e.g., SUMO-targeted ubiquitin ligase RNF4) [39]. These potential regulatory networks warrant systematic investigation to fully understand ERβ dynamics in metastatic progression.

The interplay between ERβ and molecular subtypes of LUAD presents complex clinical implications. While common LUAD-associated mutations (TP53, EGFR, KRAS, STK11, BRAF, ALK, ROS1, KEAP1) have been well-characterized [40], their relationship with ERβ remains incompletely understood. Notably, ERβ‘s clinical impact appears mutation-context dependent: although ERα correlates with EGFR mutation status [41], cytoplasmic ERβ expression in EGFR-mutant patients associates with poorer objective response rates, disease control rates, and median progression-free survival compared to EGFR-mutant/ERβ-negative cases [42]. Limited evidence suggests possible co-expression of ERβ with ALK-EML4 fusions (single case report) [43], while KRAS-mutant adenocarcinomas frequently exhibit ERβ expression [44] - though these observations require larger clinical validation. The paucity of data regarding ERβ‘s role in other molecular subtypes (e.g., BRAF-, ROS1-, or KEAP1-altered LUAD) highlights an important knowledge gap in precision oncology approaches.

Accumulating evidence suggests that the ERβ/CCL15/CCR1/VEGFC signaling axis we discovered likely constitutes a trans-tumoral mechanism promoting metastasis. Intriguingly, ERβ exhibits tissue-specific modulation of neutrophil function: while it recruits pro-lymphangiogenic neutrophils in LUAD (this study), it conversely induces IL-1β-dependent N1 neutrophils that suppress lung metastasis in triple-negative breast cancer and melanoma [45]. The pro-metastatic role of TANs via lymphangiogenesis is nevertheless observed in multiple cancers—gastric cancer (CD15^+^ TANs correlate with nodal metastasis) [46], bladder cancer (circDHTKD1/CXCL5/VEGFC axis; ETV4-CXCL1/8-MMP9/VEGFA axis) [14, 47], and now LUAD (CCL15-CCR1-VEGFC). Notably, the recruitment mechanisms (CCL15 vs CXCL5/CXCL1/8) and effector molecules (VEGFC-dominant vs MMP9/VEGFA-cooperative) display cancer-type specificity, suggesting evolutionary selection for distinct molecular implementations of a conserved TANs-lymphatic metastasis principle. These comparisons highlight the need for context-specific therapeutic targeting of this axis. Moreover, ASB8 plays a critical role in maintaining ERβ protein stability, which is strongly linked to the high prevalence of LN metastasis and poor prognosis in LUAD. Although TANs contribute to metastasis in multiple cancer types, the direct targeting of neutrophils in patients may cause severe immunodeficiency. Thus, ERβ is a viable therapeutic target for inhibiting TAN-driven lymphangiogenesis and LN metastasis in LUAD.

In conclusion, our study revealed the role of the ERβ-CCL15-CCR1-VEGFC pathway in TAN-associated lymphangiogenesis and LN metastasis, thereby offering novel insights into this metastatic process.

## Supplementary information

Supplementary Materials

Data set table 1

Data set table 2

## Data Availability

Data supporting the findings of this study are available from the corresponding author upon reasonable request.
