# Supplementary material for: Elevated ERβ expression driven by low ASB8-mediated ubiquitination in lung adenocarcinoma promotes lymph node metastasis via tumor-associated neutrophils
Source: Cell Death Dis. 2025 Jul 30;16(1):576. doi: 10.1038/s41419-025-07870-z (PMC12311189; doi:10.1038/s41419-025-07870-z)
Supplement: Supplementary file 1 — Supplementary Materials [file 41419_2025_7870_MOESM1_ESM.pdf]

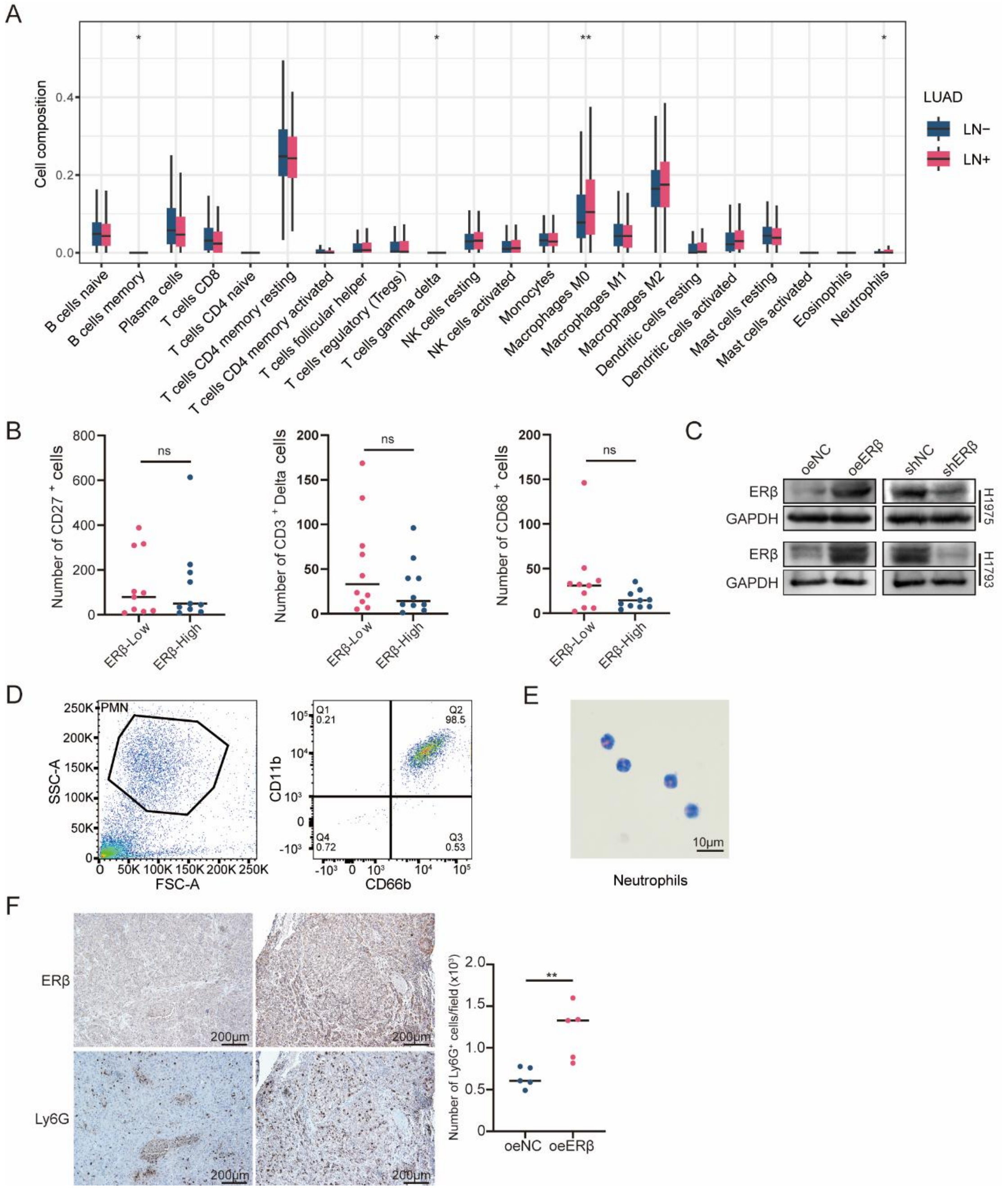

Supplementary Figure 1

Screening of Cell Subpopulations Associated with Lymph Node Metastasis and Identification of Isolated Neutrophils. A) Comparison of levels of 22 types of immune cells between LUAD (LN−) and LUAD (LN+) samples in the TCGA database. B) Quantification of $CD27^+$, CD3 $Delta^+$, and $CD68^+$ cells in ERβ-Low (n = 10) and ERβ-High (n = 10) human LUAD tissues. C) Western blot validation of ERβ expression in H1793 and H1975 cells after lentiviral transfection. D) Flow cytometry identification of $CD11b^+$ and $CD66b^+$ cells isolated from human peripheral blood. E) Wright-Giemsa staining to identify neutrophils isolated from human peripheral blood. Scale bar: 10 μm. F) Representative IHC images of

Ly6G in left lung primary tumor tissues from nude mice injected with oeNC and oeERβ H1975 constructs (left), with quantification of Ly6G$^+$ cells. Scale bar: 200 μm. Statistical significance was assessed using two-tailed t-tests. *p < 0.05, **p < 0.01, ***p < 0.001.

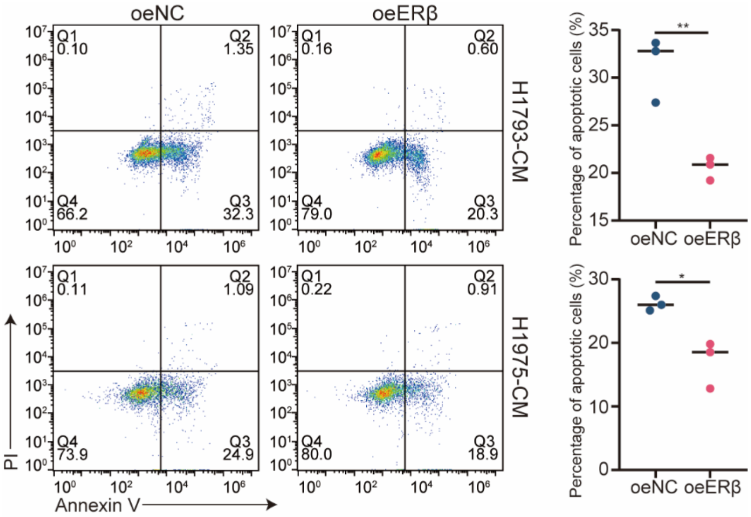

Supplementary Figure 2

Flow cytometry analysis of Annexin V and PI staining neutrophils treated as indicated.

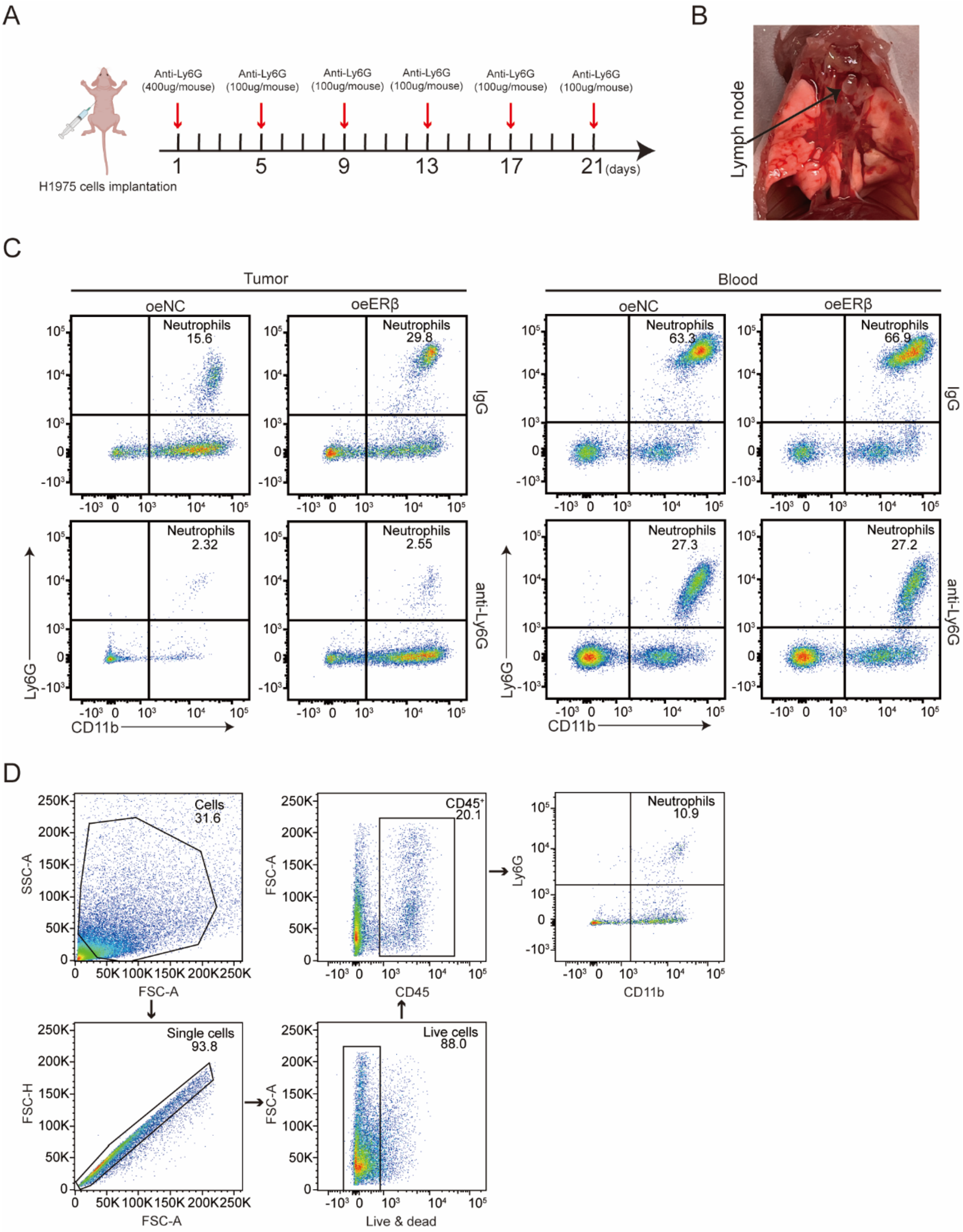

Supplementary Figure 3

Construction of an Orthotopic Lung Cancer Model with Neutrophil Depletion in Nude Mice. A) Strategy for neutrophil depletion and establishment of an orthotopic lung cancer model with mediastinal lymph node metastasis in nude mice. B) Gross images of mediastinal lymph nodes. C) Flow cytometry analysis of CD11b^{+}Ly6G^{+} neutrophils in tumor tissue and peripheral blood. D) Gating strategy for flow cytometry analysis.

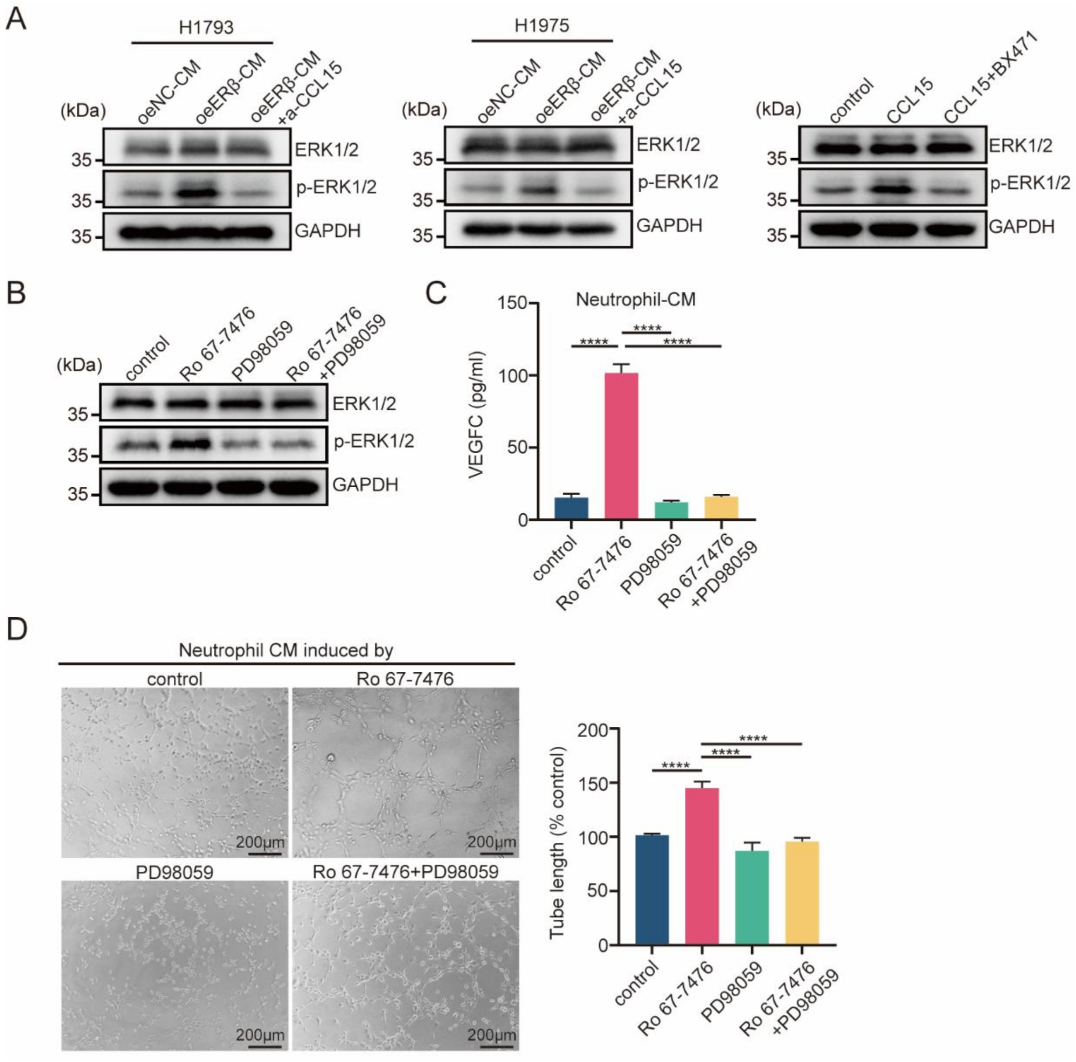

Supplementary Figure 4

Activation of Neutrophil ERK1/2 Phosphorylation via the CCL15-CCR1 Axis. A) Western blot analysis of ERK1/2 and p-ERK1/2 in neutrophils treated with tumor cell CM, CCL15 recombinant protein, CCL15 neutralizing antibody, and BX471. B) Western blot analysis of ERK1/2 and p-ERK1/2 in neutrophils treated with Ro 67-7476 and PD98059 for 12 hours. C) ELISA analysis of VEGFC levels in neutrophil CM after 12 hours of treatment with Ro 67-7476 and PD98059. D) Representative images from tube formation assays in HLECs incubated for 2 hours with neutrophil CM treated with Ro 67-7476 and PD98059 for 12 hours (top), with histograms showing the percentage of tube length in experimental versus control groups (bottom). Scale bar: 200 μm. Statistical significance was assessed using one-way ANOVA. *$p < 0.05$, **$p < 0.01$, ***$p < 0.001$.

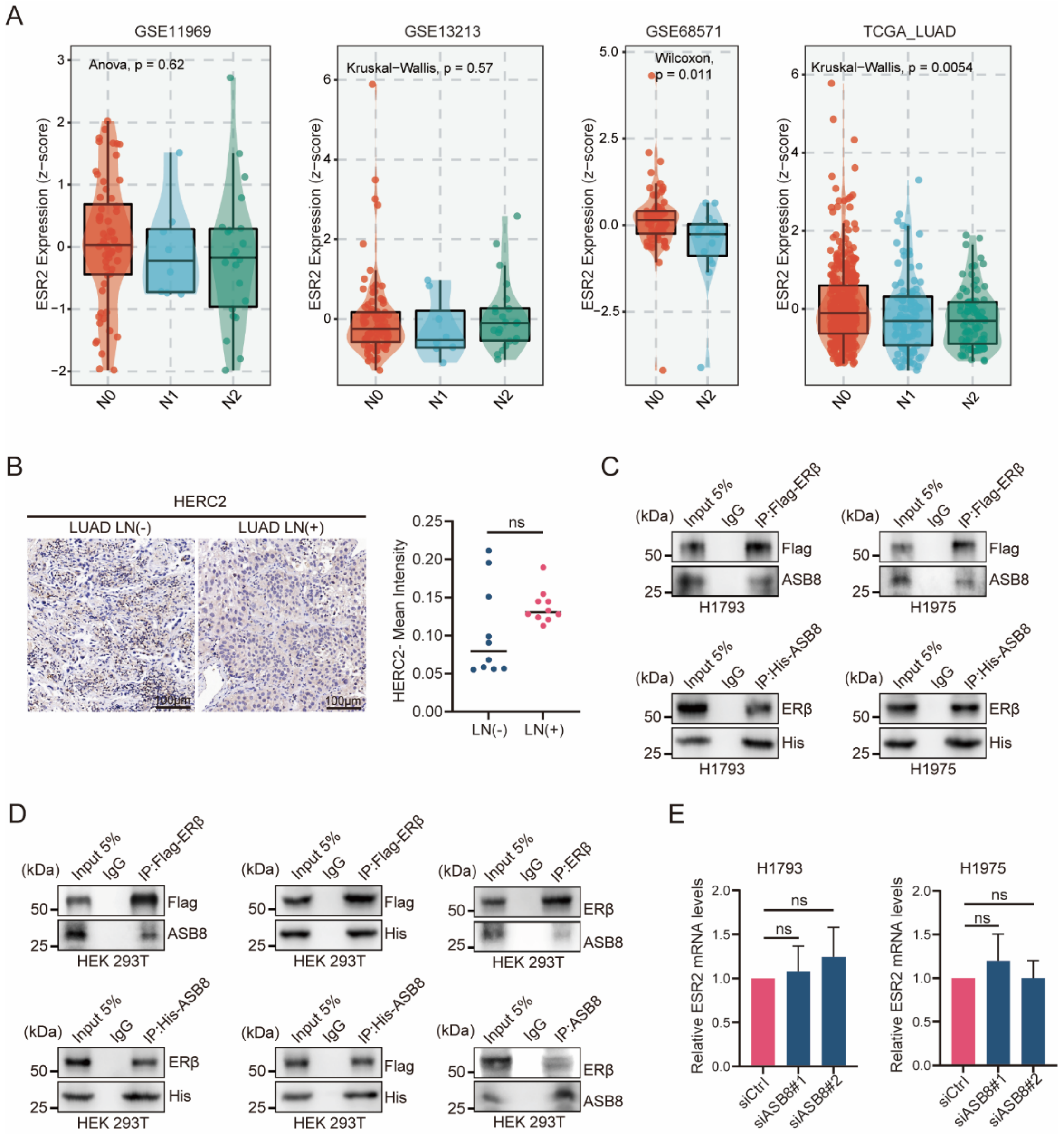

Supplementary Figure 5

Screening of ASB8-Mediated Post-Transcriptional Regulation of ERβ Protein Stability through Protein Interaction. A) Analysis of ESR2 expression differences between LUAD (LN−) and LUAD (LN+) samples in datasets GSE11969, GSE13213, GSE68571, and TCGA_LUAD using the "BEST" web tool. B) Representative IHC images of HERC2 in LUAD (LN−) (n = 10) and LUAD (LN+) (n =10) tissues (left), with quantification of HERC2 expression between the two groups (right). Scale bar: 100 μm. C) Endogenous and exogenous interaction between ERβ and ASB8 in H1793 and H1975 cells. IP was performed using antibodies against His or Flag, followed by IB. Input represents 5% of cell lysates used for immunoprecipitation. D) Endogenous and exogenous interaction between ERβ and ASB8 in HEK293T cells. Immunoprecipitation (IP) was performed using antibodies against His, Flag, ERβ, or ASB8, followed by immunoblotting (IB). Input represents 5% of cell

lysates used for immunoprecipitation. E) RT-qPCR analysis of *ESR2* mRNA in LUAD cells after ASB8 knockdown. Statistical significance was evaluated using two-tailed t-tests. *p < 0.05, **p < 0.01, ***p < 0.001, ****p < 0.0001.

Supplemental Table 1
Antibodies and Reagents.

	SOUCE	IDENTIFIER
ERβ	Proteintech	14007-1-AP
ERβ	GeneTex	GTX70174
LYVE1	Abmart	PHJ1574
CD66b	Abmart	TD10151
GAPDH	Proteintech	60004-1-Ig
Ly6G	Servicebio	GB12229
CCR1	Abmart	MU112771
MPO	Proteintech	66177-1-Ig
MPO	Proteintech	22225-1-AP
VEGFC	Proteintech	22601-1-AP
CD27	Proteintech	66308-1-Ig
CD68	Proteintech	66231-2-Ig
CD3 Delta	Proteintech	16669-1-AP
ERK1/2	Abmart	T40071
phospho-ERK1/2 (Thr202 + Tyr204)	Abmart	PC3016
Anti-Flag	Proteintech	66008
Anti-His	Proteintech	66005
Anti-HA	Proteintech	66006
ASB8	Proteintech	11735-1-AP
HERC2	Proteintech	27459-1-AP
Anti-IgG	CST	2729
HRP-conjugated Goat Anti-Rabbit IgG	Proteintech	SA00001-2
HRP-conjugated Goat Anti-Mouse IgG	Proteintech	SA00001-1
Cy3–conjugated Goat Anti-Rabbit IgG	Proteintech	SA00009-2
CoraLite488-conjugated Goat Anti-Mouse IgG	Proteintech	SA00013-1
Zombie Aqua™ Fixable Viability Kit	BioLegend	423101
FITC Anti-Mouse CD45	Elabscience	E-AB-F1136C
PerCP Anti-Mouse Ly6G	Elabscience	E-AB-F1108F
PE Anti-Mouse/Human CD11b	Elabscience	E-AB-F1081D
APC Anti-Human CD66b	Elabscience	E-AB-F1267E

	SOUCE	IDENTIFIER
Human CCL15/MIP-1 delta Antibody	R&D Systems	MAB363-SP
Recombinant Human CCL15/MIP-1 delta/LKN-1 (68 aa) Protein	R&D Systems	628-LK
BX471	MedChemExpress	HY-12080
DPN	MedChemExpress	HY-12452
Ro 67-7476	MedChemExpress	HY-100403
PD98059	MedChemExpress	HY-12028
CHX	MedChemExpress	HY-12320
MG-132	MedChemExpress	HY-13259
Chloroquine	MedChemExpress	HY-17589
PHTPP	MedChemExpress	HY-103456

Supplemental Table 2
Correlation between ERβ expression levels and clinicopathological characteristics in 44 LUAD cases from cohort 1.

		ERβ expression		
Characteristics	Cases	Low	High	*P*
Total	44			
Age (years)				0.5366
<60	23	12	11	
≥60	21	9	12	
Gender				0.2758
Female	14	5	9	
Male	30	16	14	
Tumor size (cm)				0.1548
<3	8	2	6	
>3	36	19	17	
Histological grade				0.074
Poor/moderate	25	9	16	
Well	19	12	7	
Clinical stage				0.7168
Ⅰ-Ⅱ	26	13	13	
Ⅲ-Ⅳ	18	8	10	
Lymph node metastasis				
No	22	14	8	**0.0346**
Yes	22	7	15	

Supplemental Table 3

Clinicopathological characteristics in 63 LUAD cases from Cohort 2.

Characteristics	Cases	Percentage
Total	63	
Age (years)		
<60	27	42.90%
≥60	36	57.10%
Gender		
Female	29	46%
Male	34	54%
Tumor size (cm)		
<3	25	39.70%
>3	38	60.30%
Histological grade		
Poor/moderate	42	66.70%
Well	21	33.30%
Clinical stage		
Ⅰ-Ⅱ	37	58.70%
Ⅲ-Ⅳ	26	41.30%
Lymph node metastasis		
No	36	57.10%
Yes	27	42.90%

Supplemental Table 4

List of plasmids information.

Construct	Partial Sequence
pCCL15(human)	TTTTTTTTTAATGTCTCACACAC...GTCTCCGTGGCTG
pCCL15(human)(1377-1488)	AGTCACCTGCTTGGCACTTTTCC…ATTTCTTCTGAGG
pCCL15(human)(-71-210)	TAATGTCTCACACACACACACAC…AATTTACCCATTAG
pCCL15(human)-Del(1377-1488)(-71-210)	TTTTTTTTGCATTTAGTATTGG…GTCTCCGTGGCTG
HA-UB	ATGCAGATCTTCGTGAAGACCC…TCTCAGAGGTGGG
HA-UB-K48	ATGCAGATCTTCGTGAGGACCC…TCTCAGAGGTGGG
HA-UB-K63	ATGCAGATCTTCGTGAGGACCC…TCTCAGAGGTGGG
FLAG-ESR2	ATGGATATAAAAAACTCACCAT…ACCCACAGTCTCAG
His-ASB8	ATGAGTTCCAGTATGTGGTATA…TGTTACTTTTAGAA
His-ASB8-del(235-288)	ATGAGTTCCAGTATGTGGTATA…TGGCCAGAGACCCG

The full sequence is available from the corresponding author upon request.

Supplemental Table 5

List of primers used in this study.

Primer	Sequence
CCL15 F	AGGAAGCAGTGAGCCCAGGAG
CCL15 R	CAACAAGCATGAGGCAGGAGAGG
MMP9 F	CCTGGTCCTGGTGCTCCTG
MMP9 R	GCTGCCTGTCGGTGAGATTG
VEGFD F	GCTGCCTGATGTCAACTGCTTAG
VEGFD R	GATCGCTTCACTGGTCCATGTTC
VEGFC F	AGTTACGGTCTGTGTCCAGTGTAG
VEGFC R	TGCCAGCCTCCTTTCCTTAGC
VEGFA F	CGGCGAAGAGAAGAGACACATTG
VEGFA R	GGAAGAGGATGAGGGCGAGTC
ASB8 F	TTGCTGCCATCCGTTCCTTCC
ASB8 R	ATAGTGGAGGGCTGTTCGGTTATAC
ESR2 F	GCTGAACGCCGTGACCGATG
ESR2 R	ACAGGAGCATCAGGAGGTTAGCC
GAPDH F	CATCAAGAAGGTGGTGAAGC
GAPDH R	ACCACCCTGTTGCTGTAG

Supplemental Table 6

ChIP-PCR primer sequences of CCL15.

CCL15 ChIP	Forward	Reverse
Primer pair 1	AGAACAAGGCTGAGAGTGCAA	GATTAATCTCCTGGAGGGAAGACT
Primer pair 2	GGAGACCTTCATCCTCCTGGT	GACTTCCTGGATCCTCCTCTTCTTA
Primer pair 3	AGGGCAGAGGTCAGAATGCTC	TCTGCCTGTAGCTATTGGGATAAT
Primer pair 4	TATAAGAAGAGGAGGATCCAGGAAG	CCACAGGGACAGTTCAAGTCAT
Primer pair 7	GAGGTCAGAATGCTCTTCTTTAGC	TGCCTGTAGCTATTGGGATAATTCG
Primer pair 10	CAGAGGTCAGAATGCTCTTCTTTA	TGTAGCTATTGGGATAATTCGG
Primer pair 11	TGGGTAAATTGGCCCTGTGC	ACTGAAAAGGGACACCTGGC
Primer pair 12	CAAGCTGGCCACGAAAATGG	GAAGGTCCAAGATGGGGTGG

Supplementary Methods

Immunohistochemistry

Immunohistochemistry (IHC) was performed following established protocols. Tissue sections were deparaffinized, rehydrated, and subjected to antigen retrieval with EDTA, followed by blocking with 3% hydrogen peroxide and 5% BSA. After overnight incubation at 4°C with the primary antibody, sections were treated with a secondary antibody, DAB-stained, and counterstained with hematoxylin. CD27+, CD3 Delta+, CD68+, or Ly6G+ cells were quantified in five random fields using ImageJ, while ERβ expression was assessed by measuring the average optical density of positive signals. Automated measurements were verified by two independent pathologists.

Immunofluorescence Staining

Immunofluorescence staining of FFPE tissues was performed as previously described. After deparaffinization, rehydration, and antigen retrieval in a pressure cooker (pH 9.0, 15 min), samples were blocked with 5% BSA for 30 min. Cells were fixed with 4% paraformaldehyde, washed with PBS, and permeabilized with or without 0.2% Triton X-100 before a second blocking step. Primary antibodies against MPO, ERβ, LYVE1, CD66b, VEGFC, and ASB8 were incubated overnight at 4°C, followed by fluorescently labeled secondary antibodies for 1 h and DAPI nuclear staining. Imaging was performed using a Nikon A1 confocal microscope (Nikon, Tokyo, Japan), and CD66b+, VEGFC+, MPO+, and LYVE1+ cells were quantified using ImageJ, with automated measurements cross-verified by two pathologists.

Hematoxylin and Eosin Staining （H&E Staining）

Paraffin-embedded tissue sections were deparaffinized in an oven at 65°C for 30 min, rehydrated using a series of graded ethanol solutions, and rinsed with distilled water. The sections were stained with hematoxylin for 3 min and eosin for 30~60 s, followed by rapid rinsing in distilled water. After dehydration using a graded ethanol series, sections were sealed with neutral gum, dried, and observed under a light microscope.

Lentivirus Transduction

Lentiviral vectors for ESR2 knockdown (shERβ), overexpression (oeERβ), and luciferase reporter constructs were procured from GeneChem Co., Ltd. (Shanghai, China). Transfections were performed according to the manufacturer's instructions and stable transfectants were selected using

puromycin. Luciferase activity was quantified using the Dual-Luciferase Reporter (DLR) assay system (Promega).

Western Blot

Cellular proteins were extracted using RIPA lysis buffer (G2002, Servicebio) supplemented with protease and phosphatase inhibitors. Protein concentrations were determined using the BCA Protein Assay Kit (G2026, Servicebio). Proteins were resolved on a 10% SDS-PAGE gel and transferred onto a 0.45 μm polyvinylidene fluoride membrane. The membranes were blocked with 5% BSA in Tris-buffered saline containing 0.1% Tween-20 (TBST) for 1 h and incubated overnight at 4°C with primary antibodies. Following TBST washes, the membranes were treated with HRP-conjugated secondary antibodies (1:5000) and protein detection was conducted using enhanced chemiluminescence.

RNA Isolation and Reverse Transcription quantitative Polymerase Chain Reaction (RT-qPCR)

Total RNA was extracted from the cells using TRIzol reagent (Invitrogen) according to the manufacturer's guidelines. Reverse transcription was performed using HiScript II Q RT SuperMix for qPCR (R223-01, Vazyme). RT-qPCR was performed using ChamQ Blue Universal SYBR qPCR Master Mix (#Q312-02, Vazyme) following the manufacturer's protocol. Glyceraldehyde-3-phosphate dehydrogenase (GAPDH) was used as the internal control. The gene expression levels were calculated using the 2−ΔΔCt method. The primer sequences used in this study are listed in Supplementary Table 5.

Transwell Assay

HLECs (2 × 10^5) were plated in the upper chamber of a transwell insert with an 8 μm porous membrane, while the lower chamber was filled with tumor-conditioned medium. After 24 h of incubation, cells were fixed with paraformaldehyde for 15 min and stained with crystal violet for 10 min. The stained cells on the membrane were visualized under a microscope and quantified using ImageJ software.

Flow Cytometry

Cells were resuspended in PBS and stained with fluorochrome-conjugated antibodies targeting specific markers. Flow cytometric analysis was performed using the BD FACSymphony A1 flow cytometer (BD Biosciences). The

fluorochrome-conjugated antibodies used for each marker are listed in Supplementary Table 1.

Cell Culture

The cell lines NCI-H1975, NCI-H1793, and HEK293T were obtained from the American Type Culture Collection (ATCC), while immortalized human lymphatic endothelial cells (HLEC) were sourced from Wuhan Saios. H1975 cells were cultured in RPMI 1640 medium (PM150110, Procell Life Science) with 10% fetal bovine serum (FBS; 164210, Procell Life Science) and 1% penicillin/streptomycin (PB180120, Procell Life Science). H1793 cells were maintained in DMEM/F-12 (PM150312, Procell Life Science) with the same supplements, while HEK293T cells were cultured in DMEM (PM150210, Procell Life Science). HLECs were maintained in endothelial cell medium (PM-002; SAIOS). All cell lines were cultured at 37°C in a humidified 5% CO_2 atmosphere, and experiments were conducted using mycoplasma-free cells. NCI-H1975, NCI-H1793, and HEK293T cell lines have been authenticated using Short Tandem Repeats (STR) profiling within the last 2 years. HLECs were characterized by immunofluorescence staining with specific lymphatic endothelial markers.

RNA Interference and Plasmid Constructs

Small interfering RNAs (siRNAs) targeting ASB8 and a control siRNA (siCtrl) were synthesized by Sang Biotech. The siRNA sequences used were siCtrl (5’-UUCUCCGAACGUGUCACGUTT-3’), siASB8#1 (5’-CCACUAUGCAGCAGAGAAA-3’), and siASB8#2 (5’-GGAUUACAACAAUGAUACA-3’). All plasmids were acquired from MIAOLING BIOLOGY. Supplementary Table 4 details the full-length and truncated promoter sequences of CCL15, FLAG-tagged full-length ERβ, HA-tagged full-length and truncated ubiquitin sequences, and His-tagged full-length and truncated ASB8. Transfections were performed according to the manufacturer's guidelines and previously established protocol.

Enzyme-Linked Immunosorbent Assay (ELISA)

The concentrations of CCL15 and VEGFC proteins in the culture medium were determined using ELISA kits (JM-03307H1 & JM-0622H1, JINGMEI BIOTECHNOLOGY) according to the manufacturer’s instructions. Culture medium samples were collected and centrifuged at 1000 g for 5 min to remove cellular debris. The supernatant was added to the coated wells and incubated

with an HRP-conjugated detection antibody at 37°C for 1 h. Following a series of washes, the substrate solution was added, and the plates were incubated at 37°C for 15 min. The reaction was terminated with a stop solution, and the absorbance at 450 nm was measured using a microplate reader.

Chromatin Immunoprecipitation (ChIP)-qPCR

ChIP was conducted using the SimpleChIP Enzymatic Chromatin IP Kit (9003, Cell Signaling Technology) according to the manufacturer's instructions. The cells were pretreated with 10 nM DPN for two days before collection. Cross-linking was achieved using 1% formaldehyde for 10 min followed by glycine quenching for 5 min. After washing with PBS, cells were harvested in PBS containing PMSF and subjected to micrococcal nuclease digestion at 37°C for 20 min, followed by sonication of the nuclear lysate. After centrifugation, the appropriate antibody was added to the supernatant and incubated with Protein G magnetic beads at 4°C for 2 h. Washing and chromatin elution were performed in accordance with the manufacturer's instructions. Enrichment was analyzed by qPCR using the primer sequences detailed in Supplementary Table 6.

RNA Sequencing and Analysis

Total RNA was extracted using TRIzol reagent (Invitrogen) following standard protocols and submitted to Majorbio (Shanghai, China) for sequencing. Differentially expressed genes were identified using R software (version 4.4.1) with the criteria $|log2Ratio| \geq 1$ and $p < 0.05$. Detailed Table of Differences in ERbeta Expression Levels (Data set Table 1).

Liquid Chromatography Tandem-Mass Spectrometry (LC-MS/MS) Analysis

HEK293T cells transfected with Flag-ERβ-expressing plasmids were used to identify ERβ-binding proteins. FLAG-ERβ was immunoprecipitated using Flag Magnetic Beads (MCE) and incubated overnight at 4°C. Immunocomplexes were subjected to western blotting and specific protein bands were excised for LC-MS/MS analysis performed by BGI Genomics Co., Ltd. Co-IP coupled with mass spectrometry (LC-MS/MS) data have been deposited to the ProteomeXchange Consortium via the PRIDE repository with the dataset identifier IPX0011938000. The proteins binding to ERβ were in Data set Table 2.

Group Size Determination and Statistical Power

For in vivo experiments, group sizes of n=5 mice were selected based on preliminary data showing consistent effect sizes (≥30% difference) with ~15% SD, providing 80% power to detect significant differences (α=0.05, two-tailed t-test). In vitro experiments employed n=3 biological replicates, sufficient to detect 2-fold changes (α=0.05, power>80%) based on historical experimental variability. Sample sizes were chosen to balance statistical power with ethical considerations and resource limitations.

Predefined Animal Exclusion Criteria

The exclusion of animals was strictly based on the following pre-established criteria: (1) anesthesia-related death; (2) severe postoperative complications; (3) technical errors in drug administration. All exclusion decisions were documented prior to unblinding and verified by two independent researchers. No outliers were excluded based on statistical results. Ultimately, all 5 animals per group completed the full experimental protocol, with only 1 animal per group dying from anesthesia during in vivo imaging procedures.

Randomization and Blinding Procedures

Mice were randomly assigned to treatment/control groups (n=5/group) using a computer-generated randomization sequence, stratified by baseline tumor volume to ensure balanced distribution. Cage numbers were matched to group assignments by a technician not involved in experiments, and the allocation list remained sealed until data analysis. Investigators performing tumor measurements and histology were blinded to group identities throughout the study. The surgeon performing tumor implantation and all investigators involved in endpoint measurements (caliper sizing, histopathology) were blinded to group assignments. Treatment codes were assigned by an independent technician and not revealed until statistical analysis was completed.
