# Supplementary material for: Elevated ERβ expression driven by low ASB8-mediated ubiquitination in lung adenocarcinoma promotes lymph node metastasis via tumor-associated neutrophils
Source: Cell Death Dis. 2025 Jul 30;16(1):576. doi: 10.1038/s41419-025-07870-z (PMC12311189; doi:10.1038/s41419-025-07870-z)
Supplement: Supplementary file 2 — Data set table 1 [file 41419_2025_7870_MOESM2_ESM.pdf]

Data set table 1

Detailed Table of Differences in ERβ Expression Levels

Transcript	Gene id	Gene nam	Gene desc	FC(oeESR2	Log2FC(oe	Pvalue	Padjust	Significant
ENST0000	ENSG0000	NIPAL3	NIPA like c	2.242391	1.165038	2.18E-06	0.001442	yes
ENST0000	ENSG0000	NIPAL3	NIPA like c	2.542076	1.346007	0.000322	0.030237	yes
ENST0000	ENSG0000	CD99	CD99 mole	2.056314	1.04006	9.14E-06	0.003458	yes
ENST0000	ENSG0000	DBNDD1	dysbindin	3.451385	1.787175	0.000738	0.049471	yes
ENST0000	ENSG0000	DBNDD1	dysbindin	2.761286	1.46534	1.87E-06	0.001339	yes
ENST0000	ENSG0000	PDK4	pyruvate c	8.356103	3.06283	4.35E-07	0.000553	yes
ENST0000	ENSG0000	FAM214B	family with	4.809857	2.265994	1.82E-05	0.005225	yes
ENST0000	ENSG0000	MAP3K9	mitogen-a	2.052189	1.037163	0.000664	0.047001	yes
ENST0000	ENSG0000	TTC22	tetratricop	4.175342	2.061894	1.01E-06	0.000925	yes
ENST0000	ENSG0000	TBXA2R	thromboxa	2.663796	1.413483	0.000298	0.028713	yes
ENST0000	ENSG0000	IFRD1	interferon	5.24207	2.390137	0.000564	0.042814	yes
ENST0000	ENSG0000	TEAD3	TEA doma	2.100546	1.070764	1.45E-05	0.004503	yes
ENST0000	ENSG0000	PLEKHG6	pleckstrin	3.03831	1.603269	0.000579	0.043464	yes
ENST0000	ENSG0000	NFIX	nuclear fac	0.127684	-2.96935	3.81E-05	0.008348	yes
ENST0000	ENSG0000	NFIX	nuclear fac	0.276094	-1.85677	3.83E-06	0.001943	yes
ENST0000	ENSG0000	ST3GAL1	ST3 beta-g	2.005545	1.003994	0.000139	0.018152	yes
ENST0000	ENSG0000	CD9	CD9 molec	2.640444	1.400781	5.68E-05	0.010767	yes
ENST0000	ENSG0000	CD9	CD9 molec	2.767838	1.468759	0.000181	0.021271	yes
ENST0000	ENSG0000	CD9	CD9 molec	2.936406	1.554052	6.1E-05	0.011273	yes
ENST0000	ENSG0000	FYN	FYN proto	2.547123	1.348868	0.000608	0.044646	yes
ENST0000	ENSG0000	SYT7	synaptotag	2.142171	1.099074	3.43E-06	0.001813	yes
ENST0000	ENSG0000	SEMA3B	semaphori	30.04789	4.909192	1.67E-10	2.07E-05	yes
ENST0000	ENSG0000	SEMA3B	semaphori	19.77545	4.305638	4.3E-07	0.000552	yes
ENST0000	ENSG0000	SEMA3B	semaphori	3131.511	11.61264	2.72E-06	0.001599	yes
ENST0000	ENSG0000	SEMA3B	semaphori	7.049798	2.817582	0.000474	0.038504	yes
ENST0000	ENSG0000	CALCOCO	calcium bi	2.283689	1.191366	0.000341	0.031341	yes
ENST0000	ENSG0000	MAN2B2	mannosida	2.018628	1.013375	0.000117	0.016623	yes
ENST0000	ENSG0000	CAPN1	calpain 1 [	3.607787	1.851114	9.08E-05	0.014222	yes
ENST0000	ENSG0000	ACPP	acid phosp	2.60647	1.382097	0.000222	0.024042	yes
ENST0000	ENSG0000	TTC27	tetratricop	0.496721	-1.00949	1.99E-05	0.005523	yes
ENST0000	ENSG0000	CYP24A1	cytochrom	2.62933	1.394695	5.98E-07	0.000647	yes
ENST0000	ENSG0000	CYP24A1	cytochrom	3.216693	1.685578	8.15E-05	0.013446	yes
ENST0000	ENSG0000	SNAI2	snail family	2.892271	1.532203	4.69E-06	0.002224	yes
ENST0000	ENSG0000	CD74	CD74 mole	0.241159	-2.05194	3.02E-06	0.001682	yes
ENST0000	ENSG0000	CD74	CD74 mole	0.010707	-6.5453	0.000401	0.034658	yes
ENST0000	ENSG0000	OSBPL5	oxysterol b	2.898947	1.535529	0.00037	0.032827	yes
ENST0000	ENSG0000	CD44	CD44 mole	2.008437	1.006073	0.000715	0.048706	yes
ENST0000	ENSG0000	CD44	CD44 mole	0.377769	-1.40442	5.27E-05	0.010241	yes
ENST0000	ENSG0000	TNFRSF1B	TNF recep	4.531149	2.179877	9.78E-05	0.014836	yes
ENST0000	ENSG0000	BCLAF1	BCL2 asso	3.151914	1.656228	0.000556	0.042622	yes
ENST0000	ENSG0000	GRN	granulin p	2.233792	1.159495	4.12E-05	0.008789	yes
ENST0000	ENSG0000	ZCCHC8	zinc finger	0.002536	-8.62315	0.000469	0.038333	yes
ENST0000	ENSG0000	FUT8	fucosyltrar	2.018668	1.013404	0.00065	0.046449	yes
ENST0000	ENSG0000	CYP46A1	cytochrom	300.0238	8.228933	0.000315	0.029756	yes
ENST0000	ENSG0000	DNAH5	dynein axo	0.270354	-1.88708	7.51E-06	0.003032	yes
ENST0000	ENSG0000	ADAMTS6	ADAM me	0.4026	-1.31258	0.000126	0.017315	yes
ENST0000	ENSG0000	LTBP1	latent tran	4.148858	2.052714	2.47E-06	0.001541	yes
ENST0000	ENSG0000	LTBP1	latent tran	6.347893	2.666278	0.000341	0.031341	yes
ENST0000	ENSG0000	NEDD4L	NEDD4 like	2.216134	1.148045	0.000594	0.044084	yes
ENST0000	ENSG0000	NEDD4L	NEDD4 like	2.190811	1.131465	1.65E-06	0.001217	yes
ENST0000	ENSG0000	LIMA1	LIM doma	3.107379	1.635698	1.58E-06	0.001213	yes
ENST0000	ENSG0000	PRSS8	serine prot	7.088905	2.825563	1.89E-07	0.000368	yes
ENST0000	ENSG0000	FOXN3	forkhead b	2.268509	1.181745	3.75E-05	0.008264	yes
ENST0000	ENSG0000	PLEKHH1	pleckstrin	5.555622	2.473948	0.000332	0.030872	yes
ENST0000	ENSG0000	KCNH2	potassium	3.658772	1.87136	0.000629	0.045583	yes

ENST0000	ENSG0000	PRDM1	PR/SET do	5.479617	2.454075	0.000286	0.028085	yes
ENST0000	ENSG0000	TMCC3	transmeml	4.935439	2.303179	3.07E-05	0.007164	yes
ENST0000	ENSG0000	LAMC2	laminin su	2.118365	1.082952	1.29E-06	0.001061	yes
ENST0000	ENSG0000	CDK14	cyclin dep	0.41702	-1.26181	0.000216	0.023684	yes
ENST0000	ENSG0000	RASGRF1	Ras protei	49.68366	5.6347	3.09E-06	0.001707	yes
ENST0000	ENSG0000	RASGRF1	Ras protei	11.42691	3.514363	0.000138	0.018088	yes
ENST0000	ENSG0000	ATP2B4	ATPase pla	2.005278	1.003803	6.97E-05	0.012208	yes
ENST0000	ENSG0000	ATP2B4	ATPase pla	2.441782	1.287934	3.12E-06	0.001713	yes
ENST0000	ENSG0000	MXD1	MAX dime	6.593334	2.721008	4.84E-08	0.000197	yes
ENST0000	ENSG0000	SPAG4	sperm ass	2.727478	1.447568	0.00022	0.023917	yes
ENST0000	ENSG0000	CDH3	cadherin 3	0.457527	-1.12807	0.000703	0.048316	yes
ENST0000	ENSG0000	RPL18	ribosomal	2.153982	1.107006	0.0001	0.015104	yes
ENST0000	ENSG0000	CA11	carbonic a	2.315471	1.211306	0.000219	0.023882	yes
ENST0000	ENSG0000	GPC1	glypican 1	2.133104	1.092954	2.33E-05	0.006042	yes
ENST0000	ENSG0000	LIMCH1	LIM and ca	303.4555	8.245341	0.00036	0.032267	yes
ENST0000	ENSG0000	CCN5	cellular co	7.01395	2.810227	2.56E-06	0.001556	yes
ENST0000	ENSG0000	SNX24	sorting ne	2.361313	1.239589	1.5E-05	0.004597	yes
ENST0000	ENSG0000	IPO5	importin 5	0.454685	-1.13706	0.000715	0.048665	yes
ENST0000	ENSG0000	IPO5	importin 5	0.002188	-8.83619	0.00015	0.018925	yes
ENST0000	ENSG0000	COL17A1	collagen ty	2.685304	1.425085	2.57E-05	0.006414	yes
ENST0000	ENSG0000	RASSF1	Ras associ	2.406604	1.266999	4.82E-05	0.009589	yes
ENST0000	ENSG0000	ATP11A	ATPase ph	2.176864	1.122251	0.000736	0.049464	yes
ENST0000	ENSG0000	POLR1A	RNA polyr	0.373677	-1.42014	0.00012	0.016821	yes
ENST0000	ENSG0000	TTC7A	tetratricop	3.948995	1.981486	3.52E-08	0.000175	yes
ENST0000	ENSG0000	TTC7A	tetratricop	726.7301	9.505276	0.000414	0.035451	yes
ENST0000	ENSG0000	PPP2R5B	protein ph	2.591606	1.373846	0.00016	0.019721	yes
ENST0000	ENSG0000	HES2	hes family	5.670053	2.503362	1.67E-07	0.000346	yes
ENST0000	ENSG0000	FSTL3	follistatin l	0.445456	-1.16664	0.000281	0.027853	yes
ENST0000	ENSG0000	ASNS	asparagine	2.127146	1.088919	0.000441	0.036907	yes
ENST0000	ENSG0000	ST6GALNA	ST6 N-ace	2.033619	1.02405	6.67E-05	0.011823	yes
ENST0000	ENSG0000	TESK2	testis asso	5.183424	2.373905	2.28E-05	0.005955	yes
ENST0000	ENSG0000	OSBPL3	oxysterol b	2.041727	1.02979	0.000191	0.02202	yes
ENST0000	ENSG0000	ATP2B1	ATPase pla	0.469705	-1.09017	0.000137	0.018068	yes
ENST0000	ENSG0000	RPS6KA2	ribosomal	2.617414	1.388142	1.61E-06	0.001213	yes
ENST0000	ENSG0000	LMCD1	LIM and cy	2.003405	1.002454	8.31E-05	0.013564	yes
ENST0000	ENSG0000	LMCD1	LIM and cy	2.70983	1.438202	0.000114	0.016393	yes
ENST0000	ENSG0000	SPEG	striated mu	2.636712	1.39874	0.000611	0.044725	yes
ENST0000	ENSG0000	SCARB1	scavenger	2.355266	1.23589	0.000333	0.030895	yes
ENST0000	ENSG0000	TP63	tumor pro	1293.423	10.33698	4.36E-05	0.00904	yes
ENST0000	ENSG0000	CLCN4	chloride vo	2.726534	1.447068	2.15E-06	0.001442	yes
ENST0000	ENSG0000	SMARCE1	SWI/SNF r	0.388657	-1.36343	0.000517	0.040715	yes
ENST0000	ENSG0000	ADAM11	ADAM me	0.279843	-1.83731	0.000101	0.015104	yes
ENST0000	ENSG0000	CA12	carbonic a	4.1396	2.049491	1.24E-07	0.000296	yes
ENST0000	ENSG0000	CA12	carbonic a	4.061126	2.02188	1.23E-06	0.001034	yes
ENST0000	ENSG0000	BCS1L	BCS1 hom	0.001453	-9.42683	0.000311	0.029511	yes
ENST0000	ENSG0000	NUAK1	NUAK fam	3.588243	1.843278	3.69E-07	0.000509	yes
ENST0000	ENSG0000	ZNF532	zinc finger	0.414951	-1.26899	7.54E-06	0.003032	yes
ENST0000	ENSG0000	VPS9D1	VPS9 dom	3.729002	1.89879	0.000113	0.016279	yes
ENST0000	ENSG0000	CACNG4	calcium vo	2.230667	1.157475	1.57E-06	0.001213	yes
ENST0000	ENSG0000	DLG1	discs large	0.405016	-1.30395	0.00017	0.020352	yes
ENST0000	ENSG0000	TUBA3D	tubulin alp	21033.51	14.3604	8.1E-08	0.000243	yes
ENST0000	ENSG0000	PAG1	phosphop	2.692693	1.42905	3.05E-07	0.00047	yes
ENST0000	ENSG0000	MCAM	melanoma	3.126749	1.644663	7.72E-08	0.000243	yes
ENST0000	ENSG0000	JADE1	jade family	614.7106	9.263764	2.13E-06	0.001442	yes
ENST0000	ENSG0000	LAMP3	lysosomal	10.62583	3.409504	4.87E-08	0.000197	yes
ENST0000	ENSG0000	NEBL	nebulette	0.340583	-1.55392	0.000621	0.045189	yes
ENST0000	ENSG0000	SYNJ2	synaptojar	2.124972	1.087444	8.63E-05	0.013835	yes
ENST0000	ENSG0000	SYNJ2	synaptojar	4.136077	2.048263	0.000436	0.036697	yes

ENST0000	ENSG0000	TP53INP2	tumor pro	2.286119	1.192901	2.28E-05	0.005942	yes
ENST0000	ENSG0000	TP73	tumor pro	82.86077	6.372617	0.000546	0.042207	yes
ENST0000	ENSG0000	SP140	SP140 nuc	2.806871	1.488963	0.000347	0.031677	yes
ENST0000	ENSG0000	OPHN1	oligophrer	2.043668	1.031161	3.97E-06	0.002001	yes
ENST0000	ENSG0000	DNM2	dynamin 2	2.269177	1.182169	0.000596	0.044146	yes
ENST0000	ENSG0000	EPB41L2	erythrocyt	2.366267	1.242613	1.52E-05	0.004624	yes
ENST0000	ENSG0000	RABL2B	RAB, mem	0.003024	-8.36936	0.000662	0.046882	yes
ENST0000	ENSG0000	PTPRH	protein tyr	7.869933	2.976351	1.04E-08	8.33E-05	yes
ENST0000	ENSG0000	SLC4A4	solute carr	0.122643	-3.02746	6.28E-06	0.002684	yes
ENST0000	ENSG0000	PUM3	pumilio RN	0.49441	-1.01622	0.000196	0.022417	yes
ENST0000	ENSG0000	MEF2C	myocyte e	0.234527	-2.09217	0.000665	0.047001	yes
ENST0000	ENSG0000	ZNF506	zinc finger	0.003351	-8.22126	0.000486	0.039131	yes
ENST0000	ENSG0000	SEMA5B	semaphori	7.910417	2.983754	2.71E-05	0.006627	yes
ENST0000	ENSG0000	XPO1	exportin 1	0.48582	-1.04151	0.00018	0.021247	yes
ENST0000	ENSG0000	KIF3C	kinesin fan	2.053363	1.037988	2.06E-06	0.001415	yes
ENST0000	ENSG0000	MECOM	MDS1 and	0.358638	-1.4794	2.5E-06	0.001541	yes
ENST0000	ENSG0000	TTC39A	tetratricop	2.835784	1.503748	0.000154	0.019334	yes
ENST0000	ENSG0000	TTC39A	tetratricop	3.278902	1.713213	0.000214	0.023604	yes
ENST0000	ENSG0000	FAT2	FAT atypic	7.50565	2.907977	7.04E-06	0.002924	yes
ENST0000	ENSG0000	GNAO1	G protein s	2.290484	1.195652	0.000284	0.028006	yes
ENST0000	ENSG0000	TFAP2C	transcriptio	2.076854	1.0544	2.24E-05	0.005899	yes
ENST0000	ENSG0000	SULT2B1	sulfotransf	5.965443	2.576629	1.86E-05	0.005313	yes
ENST0000	ENSG0000	EPB41L1	erythrocyt	2.49973	1.321772	0.000151	0.019088	yes
ENST0000	ENSG0000	SMOX	spermine c	2.229239	1.156551	0.000548	0.042259	yes
ENST0000	ENSG0000	SLC8B1	solute carr	4.485653	2.165318	8.41E-06	0.003278	yes
ENST0000	ENSG0000	SLC8B1	solute carr	3.041948	1.604996	0.00068	0.04749	yes
ENST0000	ENSG0000	OAS1	2'-5'-oligc	3.509437	1.81124	0.000239	0.025355	yes
ENST0000	ENSG0000	OAS1	2'-5'-oligc	3.488723	1.802699	1.06E-05	0.003723	yes
ENST0000	ENSG0000	OAS1	2'-5'-oligc	2.901103	1.536601	1.25E-05	0.004103	yes
ENST0000	ENSG0000	FXYD3	FXYD dom	9.266675	3.212052	0.000126	0.017315	yes
ENST0000	ENSG0000	GMIP	GEM intera	2.040671	1.029044	1.13E-05	0.003861	yes
ENST0000	ENSG0000	OTUB2	OTU deub	8.380977	3.067118	2.57E-09	5.59E-05	yes
ENST0000	ENSG0000	SLC9A1	solute carr	2.189404	1.130539	8.2E-06	0.003222	yes
ENST0000	ENSG0000	ICAM1	intercellula	2.343639	1.22875	4.17E-06	0.002077	yes
ENST0000	ENSG0000	CERS4	ceramide s	3.298821	1.721951	0.000117	0.016581	yes
ENST0000	ENSG0000	CERS4	ceramide s	2.533546	1.341158	0.000327	0.030504	yes
ENST0000	ENSG0000	EFNB1	ephrin B1	2.274631	1.185633	0.000226	0.024335	yes
ENST0000	ENSG0000	PHGDH	phosphog	3.993128	1.997519	0.000289	0.028193	yes
ENST0000	ENSG0000	LRRFIP2	LRR bindir	0.334231	-1.58108	0.000675	0.047341	yes
ENST0000	ENSG0000	TBC1D2	TBC1 dom	4.344139	2.11907	0.000116	0.016541	yes
ENST0000	ENSG0000	TBC1D2	TBC1 dom	5.214968	2.382658	6E-06	0.002616	yes
ENST0000	ENSG0000	WHRN	whirlin [So	3.081246	1.623514	7.02E-05	0.012229	yes
ENST0000	ENSG0000	IL11	interleukin	0.314566	-1.66856	4.2E-06	0.002077	yes
ENST0000	ENSG0000	TSPAN15	tetraspanir	2.103839	1.073024	4.3E-06	0.00211	yes
ENST0000	ENSG0000	CDC34	cell divisio	3.077107	1.621575	0.000447	0.037186	yes
ENST0000	ENSG0000	CDC34	cell divisio	2.087565	1.061821	1.57E-05	0.004728	yes
ENST0000	ENSG0000	MKNK2	MAPK inte	2.487113	1.314472	4.37E-06	0.002128	yes
ENST0000	ENSG0000	CRKL	CRK like pı	2.243498	1.16575	3.64E-07	0.000509	yes
ENST0000	ENSG0000	TBC1D10A	TBC1 dom	2.350064	1.2327	1.35E-05	0.004323	yes
ENST0000	ENSG0000	SEC14L2	SEC14 like	3.116176	1.639777	8.68E-07	0.000828	yes
ENST0000	ENSG0000	SEC14L2	SEC14 like	3.619488	1.855786	5.6E-07	0.000636	yes
ENST0000	ENSG0000	CYTH4	cytohesin	14.50321	3.858301	2.44E-07	0.000407	yes
ENST0000	ENSG0000	CARD10	caspase re	3.464215	1.792528	0.00043	0.036405	yes
ENST0000	ENSG0000	MICALL1	MICAL like	2.015314	1.011005	3.22E-06	0.001742	yes
ENST0000	ENSG0000	TTC28	tetratricop	3.409273	1.769464	0.000115	0.016439	yes
ENST0000	ENSG0000	TOM1	target of n	2.008982	1.006464	3.09E-05	0.007186	yes
ENST0000	ENSG0000	PDGFB	platelet de	4.320406	2.111167	1.12E-05	0.003849	yes
ENST0000	ENSG0000	SYNGR1	synaptogy	3.574698	1.837821	0.000182	0.021365	yes

ENST0000	ENSG0000	APOL1	apolipopro	2.232265	1.158509	8.9E-05	0.014022	yes
ENST0000	ENSG0000	CACNA1I	calcium vo	15.14565	3.920832	1.01E-07	0.00026	yes
ENST0000	ENSG0000	KIAA0930	KIAA0930	2.015654	1.011248	3E-05	0.007123	yes
ENST0000	ENSG0000	KIAA0930	KIAA0930	2.169706	1.1175	2.49E-05	0.00628	yes
ENST0000	ENSG0000	TRIM9	tripartite m	4.253336	2.088595	2.79E-06	0.001614	yes
ENST0000	ENSG0000	DDHD1	DDHD dor	0.346077	-1.53084	0.000668	0.047165	yes
ENST0000	ENSG0000	TIMM9	translocase	0.445005	-1.16811	0.000577	0.043408	yes
ENST0000	ENSG0000	KIAA0586	KIAA0586	0.269851	-1.88977	0.000315	0.029762	yes
ENST0000	ENSG0000	RIN3	Ras and Ra	2.543552	1.346845	0.000137	0.018049	yes
ENST0000	ENSG0000	ITPK1	inositol-te	6.053921	2.59787	2.14E-06	0.001442	yes
ENST0000	ENSG0000	ITPK1	inositol-te	4.044173	2.015845	2.23E-08	0.00013	yes
ENST0000	ENSG0000	PAPLN	papilin, pro	442.5554	8.789714	0.00031	0.02946	yes
ENST0000	ENSG0000	PAPLN	papilin, pro	2.068744	1.048755	0.000432	0.036524	yes
ENST0000	ENSG0000	CHD8	chromodo	386.9462	8.595989	0.000736	0.049464	yes
ENST0000	ENSG0000	NFKBIA	NFKB inhib	6.046262	2.596044	5.17E-05	0.010104	yes
ENST0000	ENSG0000	NFATC2	nuclear fac	4.980174	2.316196	0.000634	0.045702	yes
ENST0000	ENSG0000	EEF1A2	eukaryotic	0.378544	-1.40147	0.000347	0.031677	yes
ENST0000	ENSG0000	ISM1	isthmin 1 [	25.60662	4.678445	3.14E-06	0.001717	yes
ENST0000	ENSG0000	CST3	cystatin C	5.92905	2.567801	4.36E-05	0.00904	yes
ENST0000	ENSG0000	CST3	cystatin C	3.372365	1.753761	8.38E-08	0.000243	yes
ENST0000	ENSG0000	CST4	cystatin S [	12541.56	13.61443	4.9E-08	0.000197	yes
ENST0000	ENSG0000	SMAD7	SMAD fam	2.856745	1.514372	2.99E-06	0.001682	yes
ENST0000	ENSG0000	STS	steroid sul	2.447319	1.291202	2.01E-05	0.005556	yes
ENST0000	ENSG0000	PRPS2	phosphori	5.352683	2.420262	0.000309	0.029457	yes
ENST0000	ENSG0000	PRPS2	phosphori	2.429155	1.280455	1.67E-06	0.00123	yes
ENST0000	ENSG0000	ABCD1	ATP bindir	2.120615	1.084483	8.91E-05	0.014022	yes
ENST0000	ENSG0000	FMR1	fragile X m	500.0619	8.965963	0.000734	0.049416	yes
ENST0000	ENSG0000	MAGT1	magnesiur	1093.499	10.09474	5.51E-05	0.010574	yes
ENST0000	ENSG0000	CD99L2	CD99 mole	2.020989	1.015061	2.87E-05	0.006881	yes
ENST0000	ENSG0000	MAGED2	MAGE fam	2.200732	1.137984	0.000547	0.042207	yes
ENST0000	ENSG0000	MAGED2	MAGE fam	2.109055	1.076596	0.000438	0.036763	yes
ENST0000	ENSG0000	SYTL4	synaptotag	2.608399	1.383164	6.3E-05	0.011415	yes
ENST0000	ENSG0000	SYTL4	synaptotag	2.32225	1.215523	6.4E-05	0.011533	yes
ENST0000	ENSG0000	NALCN	sodium lea	0.255924	-1.96621	0.000159	0.019702	yes
ENST0000	ENSG0000	NALCN	sodium lea	0.320171	-1.64309	3.34E-05	0.007571	yes
ENST0000	ENSG0000	ACP5	acid phosp	2.232452	1.158629	0.000135	0.017921	yes
ENST0000	ENSG0000	UGGT2	UDP-gluco	0.453215	-1.14173	0.000732	0.049336	yes
ENST0000	ENSG0000	SLC7A5	solute carr	3.340941	1.740255	6.46E-08	0.000234	yes
ENST0000	ENSG0000	SLC7A5	solute carr	13.74108	3.780423	0.000151	0.019017	yes
ENST0000	ENSG0000	CLUAP1	clusterin a	0.370458	-1.43262	0.000606	0.044598	yes
ENST0000	ENSG0000	TMC5	transmeml	0.349469	-1.51676	3.69E-07	0.000509	yes
ENST0000	ENSG0000	BMF	Bcl2 modif	10.29882	3.364407	1.63E-06	0.001213	yes
ENST0000	ENSG0000	BMF	Bcl2 modif	3.794163	1.923782	0.000183	0.021396	yes
ENST0000	ENSG0000	ZFAND1	zinc finger	5.034699	2.331906	9.31E-05	0.014477	yes
ENST0000	ENSG0000	RP1	RP1 axone	0.211311	-2.24256	0.000102	0.015195	yes
ENST0000	ENSG0000	RP1	RP1 axone	0.180917	-2.4666	0.000557	0.042656	yes
ENST0000	ENSG0000	DMPK	DM1 prote	2.345472	1.229879	0.000167	0.020117	yes
ENST0000	ENSG0000	DENND3	DENN dor	2.801099	1.485993	0.00015	0.018925	yes
ENST0000	ENSG0000	TNPO2	transportir	2.501872	1.323008	0.000648	0.046441	yes
ENST0000	ENSG0000	RASA4	RAS p21 p	2.355949	1.236308	0.000118	0.016733	yes
ENST0000	ENSG0000	CDK6	cyclin depe	0.428288	-1.22335	1.06E-05	0.003723	yes
ENST0000	ENSG0000	ITGB8	integrin su	3.783716	1.919804	0.000423	0.036019	yes
ENST0000	ENSG0000	GSDME	gasdermin	2.82262	1.497035	0.000524	0.04109	yes
ENST0000	ENSG0000	CAV1	caveolin 1	0.441349	-1.18001	0.000445	0.037105	yes
ENST0000	ENSG0000	MET	MET proto	0.483985	-1.04697	2.35E-06	0.001492	yes
ENST0000	ENSG0000	LFNG	LFNG O-fu	4.635784	2.212813	0.000427	0.03628	yes
ENST0000	ENSG0000	STX1A	syntaxin 1/	2.422809	1.276681	4.26E-06	0.002101	yes
ENST0000	ENSG0000	NPTX2	neuronal p	2.358145	1.237652	2.96E-05	0.007023	yes

ENST0000	ENSG0000	SERPINE1	serpin fam	0.186149	-2.42547	5.12E-09	7.38E-05	yes
ENST0000	ENSG0000	CORO2A	coronin 2A	3.743482	1.904381	2.01E-06	0.001414	yes
ENST0000	ENSG0000	RLN2	relaxin 2 [S	5.141756	2.362261	0.000419	0.035828	yes
ENST0000	ENSG0000	NCS1	neuronal c	2.070655	1.050087	0.000307	0.029418	yes
ENST0000	ENSG0000	SHB	SH2 doma	2.159555	1.110734	1.59E-06	0.001213	yes
ENST0000	ENSG0000	HPS1	HPS1 biog	2.026688	1.019124	0.000237	0.025179	yes
ENST0000	ENSG0000	MAPK8	mitogen-a	2.906907	1.539485	0.000133	0.01778	yes
ENST0000	ENSG0000	CDH23	cadherin r	255.8967	7.999418	0.00052	0.040846	yes
ENST0000	ENSG0000	VSIR	V-set imm	2.676765	1.42049	1.01E-05	0.003659	yes
ENST0000	ENSG0000	PITX3	paired like	404.1604	8.658784	0.00031	0.02946	yes
ENST0000	ENSG0000	TSPAN14	tetraspanir	2.787881	1.479169	1.57E-06	0.001213	yes
ENST0000	ENSG0000	RUNDC3A	RUN doma	4.383394	2.132048	0.000166	0.020073	yes
ENST0000	ENSG0000	RASD1	ras related	10.08565	3.334232	2.48E-05	0.006265	yes
ENST0000	ENSG0000	CYTH1	cytohesin	2.074545	1.052795	3.34E-05	0.007571	yes
ENST0000	ENSG0000	COL1A1	collagen ty	0.235094	-2.08869	2.43E-07	0.000407	yes
ENST0000	ENSG0000	ABCC3	ATP bindir	4.007219	2.002601	2.01E-07	0.000368	yes
ENST0000	ENSG0000	SLC9A3R1	SLC9A3 re	4.12713	2.045139	2.3E-06	0.001473	yes
ENST0000	ENSG0000	SLC9A3R1	SLC9A3 re	4.117133	2.04164	2.64E-07	0.000425	yes
ENST0000	ENSG0000	CDR2L	cerebellar	3.435356	1.780459	1.02E-07	0.00026	yes
ENST0000	ENSG0000	PMP22	peripheral	0.255047	-1.97117	5.7E-05	0.010792	yes
ENST0000	ENSG0000	INPP4B	inositol po	4.296496	2.103161	0.000281	0.027853	yes
ENST0000	ENSG0000	INPP4B	inositol po	4.367135	2.126687	9.99E-06	0.003659	yes
ENST0000	ENSG0000	INPP4B	inositol po	2.936882	1.554285	4.03E-05	0.008657	yes
ENST0000	ENSG0000	UGDH	UDP-gluco	2.096029	1.067658	2.69E-05	0.006587	yes
ENST0000	ENSG0000	UGDH	UDP-gluco	3.094595	1.629751	0.000673	0.04734	yes
ENST0000	ENSG0000	LPXN	leupaxin [S	3.940541	1.978394	0.000144	0.018442	yes
ENST0000	ENSG0000	UNC93B1	unc-93 ho	2.758808	1.464045	0.00013	0.017568	yes
ENST0000	ENSG0000	CPT1A	carnitine p	0.477003	-1.06793	2.06E-06	0.001415	yes
ENST0000	ENSG0000	CCND1	cyclin D1 [	2.429985	1.280947	1.48E-07	0.000326	yes
ENST0000	ENSG0000	CHORDC1	cysteine ar	0.34866	-1.52011	2.82E-05	0.006802	yes
ENST0000	ENSG0000	CHORDC1	cysteine ar	0.433676	-1.20531	0.000451	0.037387	yes
ENST0000	ENSG0000	MDK	midkine [S	11.95277	3.579273	4.11E-09	6.62E-05	yes
ENST0000	ENSG0000	TCIRG1	T cell imm	4.158159	2.055945	2.02E-06	0.001414	yes
ENST0000	ENSG0000	TCIRG1	T cell imm	3.31284	1.728069	0.00011	0.015959	yes
ENST0000	ENSG0000	EXPH5	exophilin 5	0.464549	-1.1061	5.12E-06	0.00237	yes
ENST0000	ENSG0000	VWF	von Willeb	38.25741	5.257667	0.000106	0.015572	yes
ENST0000	ENSG0000	ASIC1	acid sensir	3.896166	1.962055	1.75E-06	0.001262	yes
ENST0000	ENSG0000	SLC11A2	solute carr	28.90802	4.853398	1.18E-05	0.003983	yes
ENST0000	ENSG0000	CYP27B1	cytochrom	62.39278	5.963307	7.44E-08	0.00024	yes
ENST0000	ENSG0000	MANSC1	MANSC do	599.0711	9.226583	0.000629	0.045583	yes
ENST0000	ENSG0000	ALDH2	aldehyde c	1039.445	10.0216	2.05E-05	0.005606	yes
ENST0000	ENSG0000	SCNN1A	sodium ch	6.984449	2.804146	1.21E-05	0.00406	yes
ENST0000	ENSG0000	SCNN1A	sodium ch	3.923232	1.972043	0.000196	0.022417	yes
ENST0000	ENSG0000	OAS2	2'-5'-oligo	3.894797	1.961548	2.39E-07	0.000407	yes
ENST0000	ENSG0000	ADGRD1	adhesion G	2.501425	1.32275	0.000191	0.022026	yes
ENST0000	ENSG0000	MRPL51	mitochonc	2.214005	1.146659	0.000226	0.024335	yes
ENST0000	ENSG0000	COL12A1	collagen ty	0.193181	-2.37197	0.000363	0.032391	yes
ENST0000	ENSG0000	COL12A1	collagen ty	0.24781	-2.0127	5.36E-05	0.010366	yes
ENST0000	ENSG0000	FRK	fyn related	2.336687	1.224464	3.78E-06	0.001928	yes
ENST0000	ENSG0000	NEDD9	neural pre	3.269792	1.709199	2.22E-06	0.001451	yes
ENST0000	ENSG0000	TPD52L1	TPD52 like	9.265927	3.211935	2.12E-05	0.005759	yes
ENST0000	ENSG0000	UST	uronyl 2-s	2.779369	1.474757	6.19E-07	0.00066	yes
ENST0000	ENSG0000	ULBP1	UL16 bind	2.194747	1.134054	0.00028	0.027853	yes
ENST0000	ENSG0000	CAP2	cyclase ass	0.389713	-1.35952	0.000581	0.043491	yes
ENST0000	ENSG0000	CAP2	cyclase ass	0.181268	-2.4638	0.00014	0.018161	yes
ENST0000	ENSG0000	PTP4A1	protein tyr	28.21796	4.818542	0.000236	0.025076	yes
ENST0000	ENSG0000	ASCC3	activating	0.437601	-1.19231	0.000649	0.046449	yes
ENST0000	ENSG0000	PERP	p53 apopt	2.009405	1.006768	3.31E-05	0.00754	yes

ENST0000	ENSG0000	ARFGEF3	ARFGEF fa	4.928181	2.301055	0.000327	0.030529	yes
ENST0000	ENSG0000	SLC29A1	solute carr	4.457814	2.156336	8.51E-05	0.013756	yes
ENST0000	ENSG0000	CLIC5	chloride in	468.6875	8.872482	0.000127	0.017422	yes
ENST0000	ENSG0000	SEMA5A	semaphori	0.408969	-1.28994	0.000403	0.034767	yes
ENST0000	ENSG0000	LOX	lysyl oxida	3.268381	1.708576	0.000674	0.047341	yes
ENST0000	ENSG0000	WWC1	WW and C	2.078663	1.055656	0.000709	0.048481	yes
ENST0000	ENSG0000	UNC5A	unc-5 netr	5.705227	2.512284	7.9E-06	0.003128	yes
ENST0000	ENSG0000	BCL6	BCL6 trans	3.831384	1.937866	5.73E-07	0.000636	yes
ENST0000	ENSG0000	AMOTL2	angiomoti	2.303355	1.203737	1.98E-05	0.005519	yes
ENST0000	ENSG0000	GNAI2	G protein s	2.127088	1.08888	5.58E-06	0.002513	yes
ENST0000	ENSG0000	GNAI2	G protein s	3.468857	1.794461	0.000159	0.019702	yes
ENST0000	ENSG0000	C3orf52	chromoson	459.8324	8.844964	0.000123	0.017193	yes
ENST0000	ENSG0000	KLHL24	kelch like f	2.213026	1.14602	0.00053	0.041366	yes
ENST0000	ENSG0000	TFCP2L1	transcriptio	5.077118	2.34401	2.89E-08	0.000155	yes
ENST0000	ENSG0000	OTOF	otoferlin [S	799.4547	9.642873	3.57E-05	0.007973	yes
ENST0000	ENSG0000	RTN4	reticulon 4	0.480518	-1.05734	6.11E-05	0.011273	yes
ENST0000	ENSG0000	EVA1A	eva-1 hom	0.316648	-1.65905	0.000686	0.047735	yes
ENST0000	ENSG0000	EFEMP1	EGF contai	0.353765	-1.49914	0.000723	0.049057	yes
ENST0000	ENSG0000	EFEMP1	EGF contai	0.361558	-1.4677	4.54E-06	0.002186	yes
ENST0000	ENSG0000	EFEMP1	EGF contai	0.353211	-1.5014	1.45E-05	0.004498	yes
ENST0000	ENSG0000	GLS	glutaminas	0.291526	-1.77831	1.63E-05	0.004788	yes
ENST0000	ENSG0000	GLS	glutaminas	0.30943	-1.69231	4.13E-06	0.002063	yes
ENST0000	ENSG0000	GLS	glutaminas	0.299653	-1.73863	1.79E-05	0.005176	yes
ENST0000	ENSG0000	GLS	glutaminas	0.360263	-1.47288	8.15E-07	0.000788	yes
ENST0000	ENSG0000	IL1RL1	interleukin	0.218383	-2.19507	0.000408	0.035101	yes
ENST0000	ENSG0000	FHL2	four and a	2.855398	1.513692	2.9E-06	0.001653	yes
ENST0000	ENSG0000	FHL2	four and a	4.040103	2.014392	5E-07	0.000605	yes
ENST0000	ENSG0000	UXS1	UDP-glucu	2.2806	1.189414	2.49E-05	0.00628	yes
ENST0000	ENSG0000	SLC1A4	solute carr	3.485768	1.801476	1.25E-07	0.000296	yes
ENST0000	ENSG0000	KYNU	kynurenina	5.972842	2.578418	1.97E-06	0.001397	yes
ENST0000	ENSG0000	KYNU	kynurenina	3.912322	1.968025	0.000168	0.020174	yes
ENST0000	ENSG0000	THADA	THADA arı	0.359735	-1.475	1.58E-05	0.004739	yes
ENST0000	ENSG0000	THADA	THADA arı	0.391138	-1.35425	7.33E-05	0.012532	yes
ENST0000	ENSG0000	MSH6	mutS hom	0.47177	-1.08384	6.6E-05	0.011743	yes
ENST0000	ENSG0000	EPHA4	EPH recep	6.530217	2.707131	4.32E-06	0.002115	yes
ENST0000	ENSG0000	QSOX1	quiescin su	3.435809	1.78065	4.15E-07	0.000542	yes
ENST0000	ENSG0000	QSOX1	quiescin su	3.452056	1.787456	1.31E-07	0.000301	yes
ENST0000	ENSG0000	KCNC4	potassium	2.520814	1.33389	0.000745	0.049699	yes
ENST0000	ENSG0000	RHOU	ras homolo	2.188494	1.129938	0.000709	0.048481	yes
ENST0000	ENSG0000	DNAJC6	DnaJ heat	0.206646	-2.27477	0.000248	0.025935	yes
ENST0000	ENSG0000	NCF2	neutrophil	13.02001	3.702658	4.75E-05	0.0095	yes
ENST0000	ENSG0000	NCF2	neutrophil	13.31575	3.735062	2.76E-05	0.006703	yes
ENST0000	ENSG0000	NCF2	neutrophil	10.90302	3.446655	1.59E-07	0.000343	yes
ENST0000	ENSG0000	WLS	Wnt ligand	2.655204	1.408823	7.78E-06	0.003091	yes
ENST0000	ENSG0000	CRYZ	crystallin z	2.901556	1.536827	0.000215	0.023614	yes
ENST0000	ENSG0000	RIMS3	regulating	5.397419	2.43227	1.93E-07	0.000368	yes
ENST0000	ENSG0000	PADI2	peptidyl ar	3.869417	1.952116	0.000156	0.019479	yes
ENST0000	ENSG0000	ECE1	endothelir	2.991128	1.58069	5.29E-07	0.000618	yes
ENST0000	ENSG0000	PTBP2	polypyrimi	0.417836	-1.25899	7.53E-05	0.012762	yes
ENST0000	ENSG0000	NRP2	neuropilin	0.173786	-2.52462	0.000119	0.016792	yes
ENST0000	ENSG0000	NRP2	neuropilin	0.230749	-2.1156	2.02E-07	0.000368	yes
ENST0000	ENSG0000	NRP2	neuropilin	0.233883	-2.09614	2.64E-05	0.006523	yes
ENST0000	ENSG0000	NRP2	neuropilin	0.272466	-1.87585	4.91E-06	0.002294	yes
ENST0000	ENSG0000	RXYLT1	ribitol xylo	0.494455	-1.01609	0.000711	0.048544	yes
ENST0000	ENSG0000	TJP2	tight junct	0.471897	-1.08346	0.00037	0.032827	yes
ENST0000	ENSG0000	C1orf198	chromoson	2.208738	1.143222	2.58E-06	0.001561	yes
ENST0000	ENSG0000	NEK6	NIMA relat	0.002075	-8.9125	0.000303	0.029137	yes
ENST0000	ENSG0000	IFI27L2	interferon	2.847228	1.509558	0.000345	0.031583	yes

ENST0000	ENSG0000	MLH3	mutL hom	0.334787	-1.57868	0.000204	0.023015	yes
ENST0000	ENSG0000	PPP4R4	protein ph	4.971618	2.313715	0.000281	0.027869	yes
ENST0000	ENSG0000	YPEL5	yippee like	2.500403	1.32216	7.13E-05	0.012317	yes
ENST0000	ENSG0000	YPEL5	yippee like	2.347223	1.230955	3.63E-05	0.008091	yes
ENST0000	ENSG0000	OGFRL1	opioid gro	3.009456	1.589503	1.22E-05	0.00406	yes
ENST0000	ENSG0000	OGFRL1	opioid gro	2.760924	1.465151	1.77E-05	0.005137	yes
ENST0000	ENSG0000	SMAD9	SMAD fam	11.32971	3.502039	0.00019	0.02191	yes
ENST0000	ENSG0000	TGFBI	transformi	0.232515	-2.1046	1.19E-05	0.003986	yes
ENST0000	ENSG0000	TGFBI	transformi	0.360311	-1.47269	6.12E-08	0.000227	yes
ENST0000	ENSG0000	UTP20	UTP20 sma	0.432723	-1.20848	1.25E-06	0.001041	yes
ENST0000	ENSG0000	DUSP4	dual speci	5.10374	2.351555	6.3E-05	0.011415	yes
ENST0000	ENSG0000	DUSP4	dual speci	4.251208	2.087873	1.1E-07	0.000275	yes
ENST0000	ENSG0000	PDLIM2	PDZ and L	4.599682	2.201534	0.000693	0.047905	yes
ENST0000	ENSG0000	PDLIM2	PDZ and L	4.152678	2.054042	0.000186	0.0216	yes
ENST0000	ENSG0000	ZNF211	zinc finger	0.002224	-8.81295	0.000169	0.020288	yes
ENST0000	ENSG0000	ADGRB2	adhesion (	8.203038	3.036158	0.000107	0.015589	yes
ENST0000	ENSG0000	ZMIZ2	zinc finger	4.33228	2.115126	0.000377	0.033159	yes
ENST0000	ENSG0000	EEPD1	endonucle	3.082204	1.623962	8.57E-07	0.000823	yes
ENST0000	ENSG0000	ARL4A	ADP ribosy	0.439154	-1.1872	0.000105	0.015451	yes
ENST0000	ENSG0000	SLC25A51	solute carr	2.835629	1.503669	0.000294	0.028507	yes
ENST0000	ENSG0000	CHST3	carbohydr	2.179509	1.124003	2.05E-06	0.001415	yes
ENST0000	ENSG0000	ADGRE5	adhesion (	2.139359	1.097178	0.000247	0.025926	yes
ENST0000	ENSG0000	NLN	neurolysin	0.453043	-1.14228	2.54E-06	0.001555	yes
ENST0000	ENSG0000	LRP1	LDL recept	2.321999	1.215367	2.49E-06	0.001541	yes
ENST0000	ENSG0000	G0S2	G0/G1 swi	2.209013	1.143402	1.17E-05	0.003971	yes
ENST0000	ENSG0000	MXD4	MAX dime	2.28517	1.192301	2.1E-05	0.005735	yes
ENST0000	ENSG0000	SLC12A4	solute carr	2.128829	1.09006	1.55E-05	0.004682	yes
ENST0000	ENSG0000	KCNK15	potassium	10.13366	3.341084	7.38E-05	0.012563	yes
ENST0000	ENSG0000	IQSEC2	IQ motif a	2.212966	1.145981	0.000569	0.043097	yes
ENST0000	ENSG0000	F13A1	coagulatio	12.15024	3.602913	0.000228	0.024519	yes
ENST0000	ENSG0000	SERPINB6	serpin fam	2.947999	1.559736	0.000335	0.031085	yes
ENST0000	ENSG0000	SERPINB6	serpin fam	3.106197	1.635149	4.88E-06	0.00229	yes
ENST0000	ENSG0000	SERPINB6	serpin fam	3.410498	1.769983	0.000619	0.045096	yes
ENST0000	ENSG0000	SERPINB6	serpin fam	4.429818	2.147247	2.26E-05	0.005932	yes
ENST0000	ENSG0000	SERPINB6	serpin fam	3.910766	1.967451	6.69E-07	0.00069	yes
ENST0000	ENSG0000	CDKN1A	cyclin dep	2.204099	1.140189	2.76E-05	0.006703	yes
ENST0000	ENSG0000	SOX4	SRY-box t	2.156692	1.10882	1.27E-06	0.001053	yes
ENST0000	ENSG0000	RUNX2	RUNX fam	0.29974	-1.73822	6.22E-05	0.011415	yes
ENST0000	ENSG0000	RUNX2	RUNX fam	0.289751	-1.78711	0.000283	0.027959	yes
ENST0000	ENSG0000	SH3TC1	SH3 doma	3.959518	1.985325	0.00013	0.017568	yes
ENST0000	ENSG0000	C3	compleme	9.191511	3.200302	4.65E-05	0.009366	yes
ENST0000	ENSG0000	C3	compleme	9.270797	3.212693	1.19E-08	8.84E-05	yes
ENST0000	ENSG0000	RTN2	reticulon 2	3.277924	1.712783	0.000498	0.03966	yes
ENST0000	ENSG0000	RTN2	reticulon 2	4.239474	2.083885	0.000442	0.036907	yes
ENST0000	ENSG0000	RBCK1	RANBP2-t	309.4978	8.273785	0.000351	0.031828	yes
ENST0000	ENSG0000	NR1D1	nuclear re	3.10751	1.635759	1.93E-05	0.005445	yes
ENST0000	ENSG0000	IFI6	interferon	4.730493	2.241991	7.66E-06	0.003067	yes
ENST0000	ENSG0000	IFI6	interferon	4.487685	2.165971	9.8E-05	0.014836	yes
ENST0000	ENSG0000	HSPA2	heat shock	2.755308	1.462213	6.42E-07	0.000678	yes
ENST0000	ENSG0000	SLC10A3	solute carr	2.370848	1.245403	2.35E-05	0.006055	yes
ENST0000	ENSG0000	ABHD8	abhydrolas	3.714906	1.893326	8.21E-05	0.013484	yes
ENST0000	ENSG0000	SIN3B	SIN3 trans	3.611954	1.85278	2.27E-07	0.000395	yes
ENST0000	ENSG0000	SGCE	sarcoglyca	0.002916	-8.42158	0.000456	0.037615	yes
ENST0000	ENSG0000	SGCE	sarcoglyca	484.5948	8.920635	0.000242	0.025553	yes
ENST0000	ENSG0000	ZFP36	ZFP36 ring	2.191772	1.132098	0.00025	0.026118	yes
ENST0000	ENSG0000	MGAT3	mannosyl	276.6687	8.112016	6.75E-06	0.00283	yes
ENST0000	ENSG0000	MGAT3	mannosyl	795.388	9.635515	3.46E-05	0.007776	yes
ENST0000	ENSG0000	A4GALT	alpha 1,4-	4.937898	2.303897	0.000318	0.029974	yes

ENST0000(	ENSG0000	A4GALT	alpha 1,4-	4.439978	2.150553	3.99E-05	0.008599	yes
ENST0000(	ENSG0000	RAC2	Rac family	3.646577	1.866543	3.82E-07	0.000516	yes
ENST0000(	ENSG0000	SPECC1	sperm anti	2.004118	1.002967	1.03E-05	0.003703	yes
ENST0000(	ENSG0000	CPA4	carboxype	0.367676	-1.44349	0.000535	0.041687	yes
ENST0000(	ENSG0000	PODXL	podocalyx	7.338928	2.875569	1.19E-05	0.003986	yes
ENST0000(	ENSG0000	PODXL	podocalyx	7.114715	2.830806	2.99E-09	5.59E-05	yes
ENST0000(	ENSG0000	LRRC4	leucine ricl	7.716729	2.947989	9.95E-07	0.000919	yes
ENST0000(	ENSG0000	IRF5	interferon	1229.982	10.26442	4.79E-05	0.009542	yes
ENST0000(	ENSG0000	CGNL1	cingulin lik	3.607038	1.850815	1.02E-06	0.00093	yes
ENST0000(	ENSG0000	DLL4	delta like c	18.56986	4.214891	3.03E-05	0.007139	yes
ENST0000(	ENSG0000	CHAC1	ChaC gluta	4.224045	2.078625	9.79E-06	0.003618	yes
ENST0000(	ENSG0000	PALLD	palladin, c	0.449083	-1.15495	5.23E-05	0.010189	yes
ENST0000(	ENSG0000	FOXA1	forkhead b	5.99816	2.58452	7.33E-06	0.003001	yes
ENST0000(	ENSG0000	FGF13	fibroblast (	0.496635	-1.00974	0.000668	0.047165	yes
ENST0000(	ENSG0000	CDKN1C	cyclin dep	8.405146	3.071273	0.000301	0.028982	yes
ENST0000(	ENSG0000	NECTIN2	nectin cell	3.606143	1.850457	2.37E-05	0.006092	yes
ENST0000(	ENSG0000	NECTIN2	nectin cell	4.150333	2.053227	2.78E-06	0.001614	yes
ENST0000(	ENSG0000	NECTIN2	nectin cell	3.341171	1.740354	0.00022	0.023898	yes
ENST0000(	ENSG0000	NECTIN2	nectin cell	3.146782	1.653877	0.000143	0.018357	yes
ENST0000(	ENSG0000	ATP8B3	ATPase ph	3.554203	1.829526	1.94E-06	0.001384	yes
ENST0000(	ENSG0000	PXDN	peroxidasi	3.284954	1.715873	2.81E-07	0.000443	yes
ENST0000(	ENSG0000	ZBTB46	zinc finger	3.564554	1.833722	2.48E-05	0.006265	yes
ENST0000(	ENSG0000	HELZ2	helicase w	2.06557	1.04654	0.00016	0.019763	yes
ENST0000(	ENSG0000	SAMD10	sterile alpł	2.041431	1.029581	0.000383	0.033585	yes
ENST0000(	ENSG0000	LAMA5	laminin su	3.574372	1.83769	3.61E-06	0.001881	yes
ENST0000(	ENSG0000	ASS1	argininosu	3.03934	1.603758	7.16E-07	0.000711	yes
ENST0000(	ENSG0000	SLC6A8	solute carr	2.247939	1.168603	4.83E-05	0.009589	yes
ENST0000(	ENSG0000	RAB11FIP4	RAB11 fam	2.721813	1.444568	6.13E-05	0.011298	yes
ENST0000(	ENSG0000	RAB11FIP4	RAB11 fam	3.838065	1.940379	3.8E-07	0.000516	yes
ENST0000(	ENSG0000	SH3BP5	SH3 doma	2.235233	1.160425	3.24E-05	0.007421	yes
ENST0000(	ENSG0000	AOC2	amine oxic	0.004592	-7.76655	0.000673	0.04733	yes
ENST0000(	ENSG0000	ANO1	anoctamin	29.82377	4.898391	0.000131	0.017674	yes
ENST0000(	ENSG0000	TNS4	tensin 4 [S	9.538109	3.253703	0.000737	0.049465	yes
ENST0000(	ENSG0000	TNS4	tensin 4 [S	7.846366	2.972025	1.58E-08	9.94E-05	yes
ENST0000(	ENSG0000	RARA	retinoic ac	2.413534	1.271147	0.000308	0.029435	yes
ENST0000(	ENSG0000	PTPRE	protein tyr	2.054203	1.038579	2.18E-05	0.005845	yes
ENST0000(	ENSG0000	VPS13B	vacuolar p	0.213391	-2.22843	9.96E-05	0.015022	yes
ENST0000(	ENSG0000	MATN2	matrilin 2 |	9.745901	3.284796	1.05E-06	0.000947	yes
ENST0000(	ENSG0000	MATN2	matrilin 2 |	10.55403	3.399722	0.000439	0.036807	yes
ENST0000(	ENSG0000	MATN2	matrilin 2 |	7.525969	2.911877	0.000157	0.01952	yes
ENST0000(	ENSG0000	MATN2	matrilin 2 |	6.637484	2.730636	0.000158	0.019631	yes
ENST0000(	ENSG0000	NASP	nuclear au	0.488743	-1.03285	0.000631	0.045587	yes
ENST0000(	ENSG0000	VSTM2L	V-set and	2.919461	1.545702	4.61E-06	0.002206	yes
ENST0000(	ENSG0000	EPSTI1	epithelial s	5.184617	2.374238	0.000122	0.017009	yes
ENST0000(	ENSG0000	CNDP2	carnosine	517.8893	9.0165	0.000123	0.017159	yes
ENST0000(	ENSG0000	LARGE1	LARGE xyl(	352.0589	8.459673	0.000308	0.029435	yes
ENST0000(	ENSG0000	C1QTNF6	C1q and T	3.116083	1.639734	6.5E-07	0.000678	yes
ENST0000(	ENSG0000	C1QTNF6	C1q and T	3.740115	1.903083	0.000516	0.040686	yes
ENST0000(	ENSG0000	TEX15	testis expr(	0.416544	-1.26346	6.09E-05	0.011266	yes
ENST0000(	ENSG0000	TTC9	tetratricop	2.972902	1.571872	1.26E-05	0.004118	yes
ENST0000(	ENSG0000	EIF2S1	eukaryotic	0.304518	-1.7154	0.000461	0.037873	yes
ENST0000(	ENSG0000	CTIF	cap bindin	3.012106	1.590773	8.88E-06	0.003402	yes
ENST0000(	ENSG0000	SPIRE1	spire type	709.5058	9.470671	0.000137	0.018068	yes
ENST0000(	ENSG0000	RSAD2	radical S-a	2.790602	1.480576	1.08E-05	0.003774	yes
ENST0000(	ENSG0000	CABLES1	Cdk5 and ,	5.441232	2.443933	4.37E-05	0.00904	yes
ENST0000(	ENSG0000	CABLES1	Cdk5 and ,	2.533142	1.340928	3.98E-05	0.008589	yes
ENST0000(	ENSG0000	RNF138	ring finger	0.362523	-1.46386	0.000399	0.034515	yes
ENST0000(	ENSG0000	FADS2	fatty acid (	3.457939	1.789913	2.59E-05	0.006447	yes

ENST0000	ENSG0000	TCN1	transcobal	29.49046	4.882177	1.47E-08	9.68E-05	yes
ENST0000	ENSG0000	CTSL	cathepsin	2.080976	1.05726	0.000153	0.019247	yes
ENST0000	ENSG0000	CTSL	cathepsin	2.446486	1.290711	5.02E-07	0.000605	yes
ENST0000	ENSG0000	PKIB	cAMP-dep	3.816543	1.932266	7.5E-06	0.003032	yes
ENST0000	ENSG0000	PKIB	cAMP-dep	2.677177	1.420712	0.000438	0.036763	yes
ENST0000	ENSG0000	SMPD2	sphingomy	2.005815	1.004189	0.000572	0.043121	yes
ENST0000	ENSG0000	STX11	syntaxin 1	2.736397	1.452278	3.77E-05	0.008297	yes
ENST0000	ENSG0000	DYSF	dysferlin [S	3.096372	1.630579	1.17E-06	0.00101	yes
ENST0000	ENSG0000	DYSF	dysferlin [S	3.314552	1.728814	6.99E-06	0.002913	yes
ENST0000	ENSG0000	KCNMB4	potassium	2.290992	1.195972	0.000135	0.017921	yes
ENST0000	ENSG0000	CPM	carboxype	5.140463	2.361898	3.54E-07	0.000503	yes
ENST0000	ENSG0000	KIAA0513	KIAA0513	8.275216	3.048797	0.000164	0.019966	yes
ENST0000	ENSG0000	SLC19A3	solute carr	4.734368	2.243172	0.000104	0.015379	yes
ENST0000	ENSG0000	DNAJB2	DnaJ heat	2.295267	1.198662	0.000235	0.02504	yes
ENST0000	ENSG0000	CYP27A1	cytochrom	3.625387	1.858135	7.19E-06	0.00296	yes
ENST0000	ENSG0000	FLNB	filamin B [S	4741.633	12.21117	8.72E-05	0.01391	yes
ENST0000	ENSG0000	LCP1	lymphocyt	2.059946	1.042607	2.77E-05	0.006704	yes
ENST0000	ENSG0000	SETDB2	SET domai	2.223053	1.152542	0.000407	0.035057	yes
ENST0000	ENSG0000	TTYH3	tweety fam	4.839478	2.274851	0.00021	0.023313	yes
ENST0000	ENSG0000	TTYH3	tweety fam	2.549725	1.350342	9.19E-05	0.014335	yes
ENST0000	ENSG0000	ZFHX2	zinc finger	4.191577	2.067493	0.000258	0.026621	yes
ENST0000	ENSG0000	ADAMTS7	ADAM me	17.48821	4.128311	1.2E-06	0.001026	yes
ENST0000	ENSG0000	ABHD17C	abhydrolas	2.120731	1.084561	6.24E-05	0.011415	yes
ENST0000	ENSG0000	HS6ST1	heparan su	2.332766	1.222041	2.73E-06	0.001599	yes
ENST0000	ENSG0000	KLF4	Kruppel lik	2.270343	1.18291	0.000139	0.018142	yes
ENST0000	ENSG0000	KLF4	Kruppel lik	3.170352	1.664643	0.000257	0.026603	yes
ENST0000	ENSG0000	TMOD1	tropomod	12942.98	13.65988	3.09E-09	5.59E-05	yes
ENST0000	ENSG0000	DAB2IP	DAB2 inter	2.60161	1.379405	0.000234	0.024961	yes
ENST0000	ENSG0000	TEX10	testis expr	0.002969	-8.39588	0.000498	0.039652	yes
ENST0000	ENSG0000	DNAJB5	DnaJ heat	3.687107	1.882489	1.59E-06	0.001213	yes
ENST0000	ENSG0000	PIM1	Pim-1 pro	2.411012	1.269639	1.3E-06	0.001061	yes
ENST0000	ENSG0000	ARRB1	arrestin be	3.146229	1.653623	4.69E-06	0.002224	yes
ENST0000	ENSG0000	ARRB1	arrestin be	5.48773	2.45621	2.78E-07	0.000442	yes
ENST0000	ENSG0000	RDX	radixin [So	4.408404	2.140256	5.06E-05	0.009965	yes
ENST0000	ENSG0000	TMPRSS13	transmeml	10.33695	3.369739	0.000294	0.028507	yes
ENST0000	ENSG0000	TMPRSS13	transmeml	4.719562	2.238653	7.95E-06	0.003139	yes
ENST0000	ENSG0000	ALKBH8	alkB homo	0.476158	-1.07049	6.9E-05	0.012128	yes
ENST0000	ENSG0000	SQOR	sulfide qui	2.166428	1.115318	2.91E-05	0.00695	yes
ENST0000	ENSG0000	THBS1	thrombosp	0.462986	-1.11096	5.35E-07	0.00062	yes
ENST0000	ENSG0000	CYP19A1	cytochrom	92.17314	6.526275	0.000633	0.045664	yes
ENST0000	ENSG0000	IFI44L	interferon	2.877602	1.524867	4.56E-05	0.009296	yes
ENST0000	ENSG0000	ARHGAP29	Rho GTPas	0.358661	-1.47931	1.31E-05	0.004218	yes
ENST0000	ENSG0000	CYP1B1	cytochrom	0.255383	-1.96926	2.29E-07	0.000395	yes
ENST0000	ENSG0000	PREPL	prolyl endo	0.002494	-8.6475	0.000341	0.031341	yes
ENST0000	ENSG0000	LOXL4	lysyl oxida	8.482138	3.084428	0.000124	0.017217	yes
ENST0000	ENSG0000	TACC2	transformi	7.646716	2.93484	0.000437	0.036697	yes
ENST0000	ENSG0000	ARL3	ADP ribosy	2.34415	1.229065	4.97E-05	0.009807	yes
ENST0000	ENSG0000	PLCE1	phospholip	0.437599	-1.19232	3.2E-05	0.007355	yes
ENST0000	ENSG0000	SLC40A1	solute carr	3.754896	1.908773	0.000572	0.043121	yes
ENST0000	ENSG0000	PARP9	poly(ADP-	2.372176	1.246211	0.000659	0.046816	yes
ENST0000	ENSG0000	FRAS1	Fraser extr	3.601614	1.848644	5.11E-05	0.010033	yes
ENST0000	ENSG0000	C1RL	compleme	2.648898	1.405392	6.18E-06	0.002648	yes
ENST0000	ENSG0000	CLSTN3	calsyntenir	3.554627	1.829698	5.96E-06	0.002616	yes
ENST0000	ENSG0000	MARCH9	membrane	5.960718	2.575486	0.000225	0.024297	yes
ENST0000	ENSG0000	MARCH9	membrane	3.557847	1.831004	0.000258	0.026621	yes
ENST0000	ENSG0000	INHBE	inhibin sub	14.96137	3.90317	0.000513	0.040596	yes
ENST0000	ENSG0000	FAM222A	family with	2.541668	1.345776	0.000246	0.025831	yes
ENST0000	ENSG0000	RHOF	ras homolo	2.767366	1.468514	5.05E-07	0.000605	yes

ENST0000	ENSG0000	GRTP1	growth ho	11.58944	3.534738	0.000126	0.017301	yes
ENST0000	ENSG0000	SYT16	synaptotag	2.130304	1.091059	6.98E-05	0.012208	yes
ENST0000	ENSG0000	ESR2	estrogen r	39132.79	15.25609	1.43E-08	9.68E-05	yes
ENST0000	ENSG0000	ESR2	estrogen r	26998.21	14.72058	1.05E-05	0.003703	yes
ENST0000	ENSG0000	ESR2	estrogen r	6679.595	12.70554	3.61E-08	0.000175	yes
ENST0000	ENSG0000	AK7	adenylate	2.383648	1.253171	0.000697	0.048095	yes
ENST0000	ENSG0000	TLE3	TLE family	2.024121	1.017296	0.000471	0.038369	yes
ENST0000	ENSG0000	BCL2A1	BCL2 relat	5.188952	2.375443	8.14E-05	0.013446	yes
ENST0000	ENSG0000	MFGE8	milk fat glo	3.00051	1.585208	1.29E-05	0.00419	yes
ENST0000	ENSG0000	CPNE2	copine 2 [S	2.135408	1.094511	0.00051	0.040402	yes
ENST0000	ENSG0000	MAP1LC3B	microtubu	2.010768	1.007747	2.2E-06	0.00145	yes
ENST0000	ENSG0000	OSGIN1	oxidative s	3.113078	1.638342	3.12E-05	0.007247	yes
ENST0000	ENSG0000	KSR1	kinase sup	5.040132	2.333461	1.73E-06	0.001256	yes
ENST0000	ENSG0000	KSR1	kinase sup	4.730732	2.242063	0.000145	0.01847	yes
ENST0000	ENSG0000	KSR1	kinase sup	4.498066	2.169305	3.25E-05	0.007421	yes
ENST0000	ENSG0000	TMC6	transmeml	10.64227	3.411734	8.19E-05	0.013478	yes
ENST0000	ENSG0000	TTYH2	tweety fam	0.408619	-1.29117	0.000422	0.035986	yes
ENST0000	ENSG0000	IGFBP4	insulin like	4.945691	2.306172	1.39E-07	0.000314	yes
ENST0000	ENSG0000	CACNA1A	calcium vo	0.003394	-8.20268	0.00071	0.048481	yes
ENST0000	ENSG0000	APP	amyloid be	2.021968	1.01576	1.04E-05	0.003703	yes
ENST0000	ENSG0000	APP	amyloid be	4.378131	2.130315	1.89E-05	0.005371	yes
ENST0000	ENSG0000	DOP1B	DOP1 leuc	0.454515	-1.1376	2.08E-05	0.005691	yes
ENST0000	ENSG0000	IGLON5	IgLON fam	3.814462	1.93148	0.000233	0.024939	yes
ENST0000	ENSG0000	WDTC1	WD and te	2.695042	1.430308	2.65E-05	0.00653	yes
ENST0000	ENSG0000	CELSR2	cadherin E	7.231776	2.85435	4.75E-07	0.000592	yes
ENST0000	ENSG0000	MGST3	microsoma	2.195297	1.134416	0.000261	0.026826	yes
ENST0000	ENSG0000	MGST3	microsoma	2.120269	1.084247	0.000375	0.033063	yes
ENST0000	ENSG0000	RGL1	ral guanine	4.451202	2.154195	7.98E-05	0.013315	yes
ENST0000	ENSG0000	ANXA9	annexin A9	12.33018	3.624122	1.48E-07	0.000326	yes
ENST0000	ENSG0000	TAF1A	TATA-box	0.497293	-1.00783	0.000349	0.031751	yes
ENST0000	ENSG0000	TAF1A	TATA-box	0.003342	-8.22518	0.00048	0.038842	yes
ENST0000	ENSG0000	HHIPL2	HHIP like 2	0.367069	-1.44588	9.11E-05	0.014222	yes
ENST0000	ENSG0000	C1orf43	chromosor	4.08745	2.031201	3.01E-05	0.007123	yes
ENST0000	ENSG0000	FLG	filaggrin [S	0.08635	-3.53366	1.63E-05	0.004788	yes
ENST0000	ENSG0000	LYST	lysosomal	0.369307	-1.43711	2.83E-05	0.006822	yes
ENST0000	ENSG0000	EPHX1	epoxide hy	2.42813	1.279846	2.37E-06	0.001498	yes
ENST0000	ENSG0000	PPFIA4	PTPRF inte	2.907029	1.539546	6.25E-05	0.011415	yes
ENST0000	ENSG0000	CDCA7	cell divisio	928.8625	9.859321	0.00037	0.032827	yes
ENST0000	ENSG0000	ACKR3	atypical ch	6.915579	2.78985	0.000135	0.017921	yes
ENST0000	ENSG0000	IQSEC1	IQ motif ar	2.002291	1.001652	1.34E-05	0.004275	yes
ENST0000	ENSG0000	IQSEC1	IQ motif ar	6.26532	2.647388	0.000282	0.027894	yes
ENST0000	ENSG0000	IQSEC1	IQ motif ar	2.331857	1.221479	3.39E-05	0.00765	yes
ENST0000	ENSG0000	MYH15	myosin he	4.53708	2.181764	1.03E-05	0.003703	yes
ENST0000	ENSG0000	MUC4	mucin 4, c	87.61333	6.453078	0.00074	0.049492	yes
ENST0000	ENSG0000	SETD7	SET domai	6.847356	2.775547	1.91E-05	0.0054	yes
ENST0000	ENSG0000	OTULINL	OTU deub	4.149499	2.052937	6.38E-06	0.002713	yes
ENST0000	ENSG0000	SSBP2	single stra	0.424585	-1.23588	0.00057	0.043103	yes
ENST0000	ENSG0000	RASA1	RAS p21 p	0.432123	-1.21049	4.96E-05	0.009786	yes
ENST0000	ENSG0000	ARHGAP2	Rho GTPas	3.221312	1.687648	0.00012	0.016882	yes
ENST0000	ENSG0000	CXCL14	C-X-C mo	439.1752	8.778653	1.91E-07	0.000368	yes
ENST0000	ENSG0000	TENM2	teneurin tr	0.039551	-4.66014	5.96E-06	0.002616	yes
ENST0000	ENSG0000	SLC2A12	solute carr	3.206672	1.681077	8.81E-06	0.003387	yes
ENST0000	ENSG0000	SLC22A3	solute carr	7.593036	2.924677	0.000417	0.035682	yes
ENST0000	ENSG0000	CREB5	cAMP resp	2.102133	1.071854	1.96E-05	0.005483	yes
ENST0000	ENSG0000	NIPSNAP2	nipsnap ho	0.224829	-2.1531	0.000593	0.044039	yes
ENST0000	ENSG0000	SYTL5	synaptotag	758.6395	9.567271	0.000173	0.020532	yes
ENST0000	ENSG0000	SYTL5	synaptotag	21.43473	4.421878	2.19E-09	5.59E-05	yes
ENST0000	ENSG0000	HDAC8	histone de	0.001078	-9.85793	0.000199	0.022677	yes

ENST0000	ENSG0000	FRMPD3	FERM and	474.7153	8.890919	0.00027	0.02729	yes
ENST0000	ENSG0000	FRMPD3	FERM and	2.19535	1.134451	0.000559	0.042691	yes
ENST0000	ENSG0000	ZNF185	zinc finger	3.237355	1.694815	0.000602	0.044405	yes
ENST0000	ENSG0000	ZNF185	zinc finger	2.765372	1.467474	0.000113	0.016285	yes
ENST0000	ENSG0000	ZNF185	zinc finger	3.012164	1.590801	2.25E-07	0.000395	yes
ENST0000	ENSG0000	ZNF185	zinc finger	3.299762	1.722362	4.34E-05	0.00904	yes
ENST0000	ENSG0000	RPL10	ribosomal	2.544632	1.347457	0.000671	0.04726	yes
ENST0000	ENSG0000	ATP6V0D2	ATPase H+	9.670508	3.273592	5.7E-08	0.000222	yes
ENST0000	ENSG0000	EBAG9	estrogen r	0.456475	-1.13139	0.000683	0.04759	yes
ENST0000	ENSG0000	TATDN1	TatD DNa:	0.322882	-1.63092	1.07E-05	0.003738	yes
ENST0000	ENSG0000	VLDLR	very low d	3.646634	1.866565	1.24E-05	0.004074	yes
ENST0000	ENSG0000	VLDLR	very low d	2.372438	1.246371	0.000197	0.022511	yes
ENST0000	ENSG0000	SHC3	SHC adapt	2.77343	1.471671	0.000118	0.016643	yes
ENST0000	ENSG0000	GSN	gelsolin [S	6.199702	2.632199	8.14E-06	0.003205	yes
ENST0000	ENSG0000	GSN	gelsolin [S	11.03019	3.463386	2.22E-05	0.00589	yes
ENST0000	ENSG0000	GSN	gelsolin [S	14.28034	3.835959	2.16E-06	0.001442	yes
ENST0000	ENSG0000	GSN	gelsolin [S	10.31727	3.36699	2.57E-07	0.000423	yes
ENST0000	ENSG0000	ANKRD1	ankyrin re	4.405375	2.139265	2.79E-06	0.001614	yes
ENST0000	ENSG0000	INA	internexin	2.292851	1.197143	0.00034	0.031341	yes
ENST0000	ENSG0000	ITPRIP	inositol 1,4	2.323816	1.216496	1.11E-05	0.003849	yes
ENST0000	ENSG0000	ITPRIP	inositol 1,4	2.615652	1.387171	0.000391	0.033976	yes
ENST0000	ENSG0000	DGKZ	diacylglyce	2.350374	1.23289	2.14E-05	0.005792	yes
ENST0000	ENSG0000	DGKZ	diacylglyce	3.416818	1.772653	3E-06	0.001682	yes
ENST0000	ENSG0000	PTPRJ	protein tyr	0.404967	-1.30412	4.39E-06	0.002128	yes
ENST0000	ENSG0000	CAPN5	calpain 5 [	3.240982	1.696431	9.23E-07	0.000869	yes
ENST0000	ENSG0000	CAPN5	calpain 5 [	2.758735	1.464007	6.42E-05	0.011562	yes
ENST0000	ENSG0000	MTA2	metastasis	5.798833	2.535763	0.000237	0.025183	yes
ENST0000	ENSG0000	CHEK1	checkpoin	0.390834	-1.35537	0.000284	0.028006	yes
ENST0000	ENSG0000	TAGLN	transgelin	0.232625	-2.10392	8.81E-05	0.013938	yes
ENST0000	ENSG0000	CABLES2	Cdk5 and	3.340401	1.740021	7.73E-07	0.000757	yes
ENST0000	ENSG0000	HMGA2	high mobi	0.361168	-1.46926	8.38E-05	0.013607	yes
ENST0000	ENSG0000	HMGA2	high mobi	0.460043	-1.12016	2.82E-05	0.006802	yes
ENST0000	ENSG0000	HMGA2	high mobi	0.286584	-1.80297	3.31E-06	0.001771	yes
ENST0000	ENSG0000	ABCB9	ATP bindir	2.432234	1.282282	0.000142	0.018294	yes
ENST0000	ENSG0000	UBC	ubiquitin C	8.412275	3.072496	8.23E-08	0.000243	yes
ENST0000	ENSG0000	ITPR1	inositol 1,4	0.471864	-1.08356	9.79E-05	0.014836	yes
ENST0000	ENSG0000	MAGI1	membrane	3.09209	1.628582	0.000208	0.023246	yes
ENST0000	ENSG0000	MAGI1	membrane	2.236433	1.1612	0.000707	0.048456	yes
ENST0000	ENSG0000	MAGI1	membrane	2.30557	1.205124	0.000289	0.028193	yes
ENST0000	ENSG0000	AKAP6	A-kinase a	0.430455	-1.21607	1.96E-05	0.005483	yes
ENST0000	ENSG0000	AKAP6	A-kinase a	0.480154	-1.05843	0.000164	0.019974	yes
ENST0000	ENSG0000	AKAP6	A-kinase a	0.215949	-2.21124	0.000225	0.024297	yes
ENST0000	ENSG0000	ACSL1	acyl-CoA s	2.338071	1.225319	3.07E-05	0.007164	yes
ENST0000	ENSG0000	DST	dystonin [S	0.392895	-1.34779	6.08E-06	0.002623	yes
ENST0000	ENSG0000	MZT2B	mitotic spi	2.275844	1.186402	0.000734	0.049416	yes
ENST0000	ENSG0000	MZT2B	mitotic spi	2.022775	1.016336	0.000457	0.037651	yes
ENST0000	ENSG0000	TUBA3E	tubulin alp	33344.27	15.02515	4.66E-08	0.000197	yes
ENST0000	ENSG0000	HSPB8	heat shock	10.56944	3.401827	4.85E-08	0.000197	yes
ENST0000	ENSG0000	PDK1	pyruvate c	2.191113	1.131664	0.000689	0.047835	yes
ENST0000	ENSG0000	PLEKHH2	pleckstrin	3.109104	1.636499	0.000593	0.044038	yes
ENST0000	ENSG0000	GJA1	gap juncti	2.272387	1.184208	7.06E-05	0.012232	yes
ENST0000	ENSG0000	GJA1	gap juncti	2.504597	1.324579	0.000265	0.026978	yes
ENST0000	ENSG0000	RASGRP3	RAS guany	7.538956	2.914365	2.86E-05	0.006858	yes
ENST0000	ENSG0000	ANKRD22	ankyrin re	2.613509	1.385988	0.000698	0.048102	yes
ENST0000	ENSG0000	PANK1	pantothen	0.486792	-1.03862	0.00039	0.033912	yes
ENST0000	ENSG0000	PLOD2	procollage	2.067879	1.048152	1E-05	0.003659	yes
ENST0000	ENSG0000	PLOD2	procollage	2.447572	1.291351	0.000685	0.047706	yes
ENST0000	ENSG0000	CARHSP1	calcium re	2.041137	1.029373	8.48E-05	0.013733	yes

ENST0000(	ENSG0000	CARHSP1	calcium re	2.006754	1.004864	0.000245	0.025831	yes
ENST0000(	ENSG0000	ADGRF1	adhesion (	0.319742	-1.64502	0.000133	0.017777	yes
ENST0000(	ENSG0000	PLEKHG4B	pleckstrin	0.176673	-2.50085	4.88E-06	0.00229	yes
ENST0000(	ENSG0000	DDAH1	dimethylar	3.128526	1.645483	2.06E-06	0.001415	yes
ENST0000(	ENSG0000	C16orf74	chromosor	2.708289	1.437382	0.00049	0.039253	yes
ENST0000(	ENSG0000	C16orf74	chromosor	2.658225	1.410463	9.11E-05	0.014222	yes
ENST0000(	ENSG0000	PITPNC1	phosphatic	4.717273	2.237953	0.000225	0.024297	yes
ENST0000(	ENSG0000	PITPNC1	phosphatic	3.917536	1.969946	2.18E-07	0.000391	yes
ENST0000(	ENSG0000	FAM167A	family with	2.802756	1.486846	2.44E-05	0.006215	yes
ENST0000(	ENSG0000	ACSS1	acyl-CoA s	99.71589	6.639751	0.000108	0.015731	yes
ENST0000(	ENSG0000	ACSS1	acyl-CoA s	185.5387	7.535576	2.66E-09	5.59E-05	yes
ENST0000(	ENSG0000	GOLGA7B	golgin A7	3.406166	1.768149	0.000623	0.045307	yes
ENST0000(	ENSG0000	GOLGA7B	golgin A7	2.324265	1.216774	0.000609	0.044647	yes
ENST0000(	ENSG0000	ADK	adenosine	2.365538	1.242168	4.28E-05	0.008982	yes
ENST0000(	ENSG0000	HKDC1	hexokinase	4.324345	2.112482	9.14E-08	0.00025	yes
ENST0000(	ENSG0000	HK1	hexokinase	3.066419	1.616555	3.48E-07	0.000503	yes
ENST0000(	ENSG0000	RAB11FIP1	RAB11 fam	2.234289	1.159816	6.75E-07	0.00069	yes
ENST0000(	ENSG0000	RAB11FIP1	RAB11 fam	2.139749	1.097442	4.34E-05	0.00904	yes
ENST0000(	ENSG0000	B3GNT7	UDP-GlcN	2.937222	1.554452	9.65E-07	0.000896	yes
ENST0000(	ENSG0000	NMNAT2	nicotinami	2.931055	1.55142	1.23E-06	0.001034	yes
ENST0000(	ENSG0000	STEAP2	STEAP2 m	0.434472	-1.20266	0.000103	0.015325	yes
ENST0000(	ENSG0000	TMEM164	transmeml	4.845709	2.276708	3.73E-05	0.00825	yes
ENST0000(	ENSG0000	MX1	MX dynam	4.610319	2.204867	9.5E-08	0.00025	yes
ENST0000(	ENSG0000	DGKI	diacylglyce	3.51154	1.812104	0.000347	0.031677	yes
ENST0000(	ENSG0000	XDH	xanthine d	4.002444	2.000881	0.000218	0.023804	yes
ENST0000(	ENSG0000	GPR153	G protein-	13.31708	3.735206	8.08E-09	7.81E-05	yes
ENST0000(	ENSG0000	KCNB1	potassium	4.05519	2.01977	0.000325	0.030398	yes
ENST0000(	ENSG0000	AHCYL2	adenosylh	2.089558	1.063198	0.000432	0.036513	yes
ENST0000(	ENSG0000	SLC45A3	solute carr	3.515522	1.813739	3.63E-06	0.001888	yes
ENST0000(	ENSG0000	NBL1	NBL1, DAN	2.995664	1.582876	3.81E-06	0.001937	yes
ENST0000(	ENSG0000	CDA	cytidine de	2.973168	1.572001	1.23E-06	0.001034	yes
ENST0000(	ENSG0000	PINK1	PTEN indu	2.598112	1.377463	5.68E-07	0.000636	yes
ENST0000(	ENSG0000	DMTN	dematin ac	2.419127	1.274487	0.000468	0.038309	yes
ENST0000(	ENSG0000	STC1	stanniocal	4.521204	2.176707	2.03E-07	0.000368	yes
ENST0000(	ENSG0000	RCAN1	regulator c	2.087918	1.062065	0.000138	0.018088	yes
ENST0000(	ENSG0000	ZFYVE28	zinc finger	41.01037	5.357917	0.000738	0.049471	yes
ENST0000(	ENSG0000	SLC37A1	solute carr	2.880546	1.526342	5.37E-06	0.002457	yes
ENST0000(	ENSG0000	SLC37A1	solute carr	2.371953	1.246076	0.000206	0.02315	yes
ENST0000(	ENSG0000	ZBTB7B	zinc finger	2.267971	1.181402	5.76E-05	0.010822	yes
ENST0000(	ENSG0000	CYGB	cytoglobin	6.166164	2.624373	0.000358	0.032206	yes
ENST0000(	ENSG0000	CYGB	cytoglobin	5.110628	2.3535	6.04E-06	0.002616	yes
ENST0000(	ENSG0000	AQP5	aquaporin	315.3143	8.300647	0.000437	0.036697	yes
ENST0000(	ENSG0000	RPL29	ribosomal	0.003202	-8.28694	0.000698	0.048103	yes
ENST0000(	ENSG0000	PLPP3	phospholip	2.691584	1.428455	1.23E-05	0.004071	yes
ENST0000(	ENSG0000	ZSWIM5	zinc finger	4.81422	2.267302	0.000287	0.028085	yes
ENST0000(	ENSG0000	LAPTM5	lysosomal	22.05343	4.462931	3.57E-05	0.007973	yes
ENST0000(	ENSG0000	SDC3	syndecan 3	5.873691	2.554267	9.23E-09	8.33E-05	yes
ENST0000(	ENSG0000	SDC3	syndecan 3	4.407998	2.140124	2.32E-05	0.006013	yes
ENST0000(	ENSG0000	NLRP3	NLR family	972.1286	9.925003	2.02E-05	0.005575	yes
ENST0000(	ENSG0000	NLRP3	NLR family	524.1703	9.033892	0.00016	0.019721	yes
ENST0000(	ENSG0000	OLFML2B	olfactome	0.285451	-1.80869	0.000216	0.023684	yes
ENST0000(	ENSG0000	ATF3	activating	3.330766	1.735854	4.58E-06	0.002197	yes
ENST0000(	ENSG0000	CAPN2	calpain 2 [	2.241061	1.164182	0.000205	0.023015	yes
ENST0000(	ENSG0000	SLC16A14	solute carr	0.409887	-1.2867	0.00038	0.033321	yes
ENST0000(	ENSG0000	IVL	involucrin	12.09006	3.595749	6.81E-07	0.00069	yes
ENST0000(	ENSG0000	S100A9	S100 calciu	16.25593	4.022894	4.03E-05	0.008657	yes
ENST0000(	ENSG0000	TGFA	transformi	7.051901	2.818012	6.52E-09	7.38E-05	yes
ENST0000(	ENSG0000	ALPP	alkaline ph	195.7041	7.61253	2.86E-10	2.07E-05	yes

ENST0000	ENSG0000	ALPG	alkaline ph	8.627256	3.108902	3.89E-07	0.000517	yes
ENST0000	ENSG0000	PBXIP1	PBX home	7.456255	2.898451	0.00026	0.026736	yes
ENST0000	ENSG0000	IGFN1	immunogl	0.132596	-2.91489	5.12E-05	0.010033	yes
ENST0000	ENSG0000	ELF3	E74 like ET	2.763517	1.466506	4.44E-06	0.002146	yes
ENST0000	ENSG0000	ELF3	E74 like ET	3.015454	1.592375	2.32E-06	0.001482	yes
ENST0000	ENSG0000	ADORA1	adenosine	8.408928	3.071922	0.000213	0.023565	yes
ENST0000	ENSG0000	ADAMTS9	ADAM me	0.319304	-1.647	0.000164	0.019966	yes
ENST0000	ENSG0000	TM4SF18	transmeml	0.370849	-1.4311	5.29E-06	0.002427	yes
ENST0000	ENSG0000	LIPH	lipase H [S	2.484659	1.313048	7.1E-05	0.012272	yes
ENST0000	ENSG0000	MELTF	melanotra	2.643822	1.402625	2.95E-07	0.00046	yes
ENST0000	ENSG0000	GASK1B	golgi asso	3.620786	1.856303	0.00066	0.046863	yes
ENST0000	ENSG0000	EDIL3	EGF like re	0.292877	-1.77163	2.24E-05	0.005904	yes
ENST0000	ENSG0000	EDIL3	EGF like re	0.309349	-1.69269	0.000119	0.01679	yes
ENST0000	ENSG0000	PRRC1	proline ricl	2022.428	10.98187	0.000309	0.02946	yes
ENST0000	ENSG0000	RHOBTB3	Rho relate	2.580153	1.367457	7.41E-05	0.012583	yes
ENST0000	ENSG0000	RHOBTB3	Rho relate	2.285255	1.192355	7.35E-06	0.003001	yes
ENST0000	ENSG0000	RHOBTB3	Rho relate	4.256423	2.089641	0.000218	0.023796	yes
ENST0000	ENSG0000	STXBP5	syntaxin bi	0.409364	-1.28854	2.42E-05	0.006185	yes
ENST0000	ENSG0000	RAET1E	retinoic ac	3.949966	1.98184	2.11E-05	0.005746	yes
ENST0000	ENSG0000	GALNT10	polypeptic	2.084156	1.059463	1.61E-06	0.001213	yes
ENST0000	ENSG0000	COL1A2	collagen ty	0.21951	-2.18764	9.87E-05	0.014914	yes
ENST0000	ENSG0000	HNF4G	hepatocyte	30.50076	4.930773	9.36E-06	0.003506	yes
ENST0000	ENSG0000	HNF4G	hepatocyte	43.631	5.447282	0.000587	0.043753	yes
ENST0000	ENSG0000	GPER1	G protein-	8.321267	3.056803	0.000185	0.021578	yes
ENST0000	ENSG0000	GPER1	G protein-	7.692751	2.9435	6.77E-05	0.011972	yes
ENST0000	ENSG0000	MICALL2	MICAL like	2.234413	1.159896	1.04E-05	0.003703	yes
ENST0000	ENSG0000	YWHAZ	tyrosine 3-	0.464244	-1.10705	0.000183	0.021368	yes
ENST0000	ENSG0000	CTHRC1	collagen tr	3.147982	1.654427	0.000104	0.015418	yes
ENST0000	ENSG0000	SYK	spleen ass	3.318468	1.730517	0.000647	0.046436	yes
ENST0000	ENSG0000	ABCA1	ATP bindir	0.454309	-1.13826	7.33E-06	0.003001	yes
ENST0000	ENSG0000	FBP1	fructose-b	3.632447	1.860942	0.000217	0.023776	yes
ENST0000	ENSG0000	CARD19	caspase re	2.415815	1.27251	1.9E-05	0.005391	yes
ENST0000	ENSG0000	ZCCHC24	zinc finger	2.789404	1.479957	1.45E-06	0.001148	yes
ENST0000	ENSG0000	SLC16A9	solute carr	0.185261	-2.43237	5.68E-07	0.000636	yes
ENST0000	ENSG0000	DEPP1	DEPP1 aut	3.00305	1.586429	1.22E-06	0.001034	yes
ENST0000	ENSG0000	JCAD	junctional	0.444864	-1.16856	6.16E-07	0.00066	yes
ENST0000	ENSG0000	PACSIN3	protein kir	2.215226	1.147454	8.65E-06	0.003344	yes
ENST0000	ENSG0000	IFI27	interferon	5.973519	2.578581	2.07E-06	0.001415	yes
ENST0000	ENSG0000	IFI27	interferon	5.489575	2.456694	1.62E-06	0.001213	yes
ENST0000	ENSG0000	AMOTL1	angiomoti	2.963897	1.567495	1.13E-05	0.003861	yes
ENST0000	ENSG0000	CKB	creatine ki	5.798844	2.535765	9.46E-08	0.00025	yes
ENST0000	ENSG0000	CLMP	CXADR like	2.928986	1.550401	5.21E-07	0.000613	yes
ENST0000	ENSG0000	APBB1	amyloid be	3.990014	1.996394	3.71E-05	0.00822	yes
ENST0000	ENSG0000	SYNPO2L	synaptopo	6.938105	2.794542	3.97E-05	0.008585	yes
ENST0000	ENSG0000	TPP1	tripeptidyl	2.278495	1.188081	1.12E-06	0.000978	yes
ENST0000	ENSG0000	PPFIBP2	PPFIA binc	316.3964	8.305589	0.000626	0.045477	yes
ENST0000	ENSG0000	SLFN5	schlafen fa	2.272599	1.184343	2.01E-05	0.005557	yes
ENST0000	ENSG0000	YPEL4	yippee like	31.15421	4.961355	0.000125	0.017282	yes
ENST0000	ENSG0000	YPEL4	yippee like	1248.394	10.28586	1.02E-05	0.003679	yes
ENST0000	ENSG0000	NAB2	NGFI-A bi	3.063819	1.615331	0.000101	0.015178	yes
ENST0000	ENSG0000	TEF	TEF transc	2.289819	1.195234	0.000251	0.026195	yes
ENST0000	ENSG0000	PIP5KL1	phosphatic	4.042097	2.015104	0.00026	0.02674	yes
ENST0000	ENSG0000	FAM102A	family with	3.720728	1.895585	3.71E-06	0.001908	yes
ENST0000	ENSG0000	FAM102A	family with	3.207247	1.681335	1.37E-06	0.001104	yes
ENST0000	ENSG0000	GPRC5B	G protein-	5.887155	2.557571	0.000274	0.027458	yes
ENST0000	ENSG0000	CACNB3	calcium vc	2.137068	1.095633	8.17E-05	0.013453	yes
ENST0000	ENSG0000	CYP2S1	cytochrom	2.11767	1.082478	0.000129	0.017538	yes
ENST0000	ENSG0000	TMC4	transmeml	2.021225	1.01523	3.15E-05	0.007267	yes

ENST0000	ENSG0000	ANKRD33	ankyrin re	2.457746	1.297336	0.000134	0.017841	yes
ENST0000	ENSG0000	SEMA6B	semaphori	3.607555	1.851021	5.86E-05	0.010951	yes
ENST0000	ENSG0000	SEMA6B	semaphori	3.560425	1.832049	2.17E-05	0.005836	yes
ENST0000	ENSG0000	ANGPTL4	angiopoie	11.27606	3.495191	0.000143	0.018357	yes
ENST0000	ENSG0000	CD300C	CD300c m	65.69875	6.037794	0.0002	0.022718	yes
ENST0000	ENSG0000	HID1	HID1 dom	8.90794	3.155092	7.09E-08	0.000239	yes
ENST0000	ENSG0000	MGAT5B	alpha-1,6-	2.004662	1.003359	7.66E-05	0.012898	yes
ENST0000	ENSG0000	SLC3A2	solute carr	3.15902	1.659477	0.000106	0.015522	yes
ENST0000	ENSG0000	SLC3A2	solute carr	2.638376	1.39965	0.000349	0.031751	yes
ENST0000	ENSG0000	SLC3A2	solute carr	2.322148	1.21546	9.11E-06	0.003457	yes
ENST0000	ENSG0000	SLC3A2	solute carr	3.555734	1.830147	3.29E-08	0.00017	yes
ENST0000	ENSG0000	TRANK1	tetratricop	2.921355	1.546638	2.67E-06	0.001585	yes
ENST0000	ENSG0000	TRANK1	tetratricop	1183.09	10.20834	0.000442	0.036942	yes
ENST0000	ENSG0000	ENTPD3	ectonucle	816.4365	9.673197	4.78E-05	0.009542	yes
ENST0000	ENSG0000	ENTPD3	ectonucle	21.90672	4.453302	2.79E-08	0.000155	yes
ENST0000	ENSG0000	VASN	vasorin [Sc	17.66835	4.143095	7.69E-09	7.81E-05	yes
ENST0000	ENSG0000	BDKRB2	bradykinin	4.102227	2.036407	0.000455	0.037584	yes
ENST0000	ENSG0000	BDKRB2	bradykinin	3.935965	1.976717	0.000547	0.042207	yes
ENST0000	ENSG0000	HR	HR lysine c	4.404726	2.139052	0.000105	0.015461	yes
ENST0000	ENSG0000	BMP1	bone mor	3.387929	1.760404	6.32E-05	0.011415	yes
ENST0000	ENSG0000	BMP1	bone mor	3.520457	1.815763	5.75E-07	0.000636	yes
ENST0000	ENSG0000	SERINC2	serine inc	2.498748	1.321206	6.88E-06	0.002874	yes
ENST0000	ENSG0000	AXIN2	axin 2 [Sou	525.5252	9.037616	0.000287	0.028085	yes
ENST0000	ENSG0000	IL7R	interleukin	2.392936	1.258782	7.38E-06	0.003004	yes
ENST0000	ENSG0000	AHCYL1	adenosylh	3.805522	1.928095	0.000448	0.037231	yes
ENST0000	ENSG0000	MAP2K1	mitogen-a	2.30713	1.206099	8.32E-06	0.003251	yes
ENST0000	ENSG0000	MAP2K1	mitogen-a	2.461221	1.299374	8.29E-05	0.013553	yes
ENST0000	ENSG0000	VXN	vexin [Sou	3.276452	1.712135	0.00021	0.023339	yes
ENST0000	ENSG0000	MN1	MN1 proto	3.992227	1.997194	1.09E-06	0.000967	yes
ENST0000	ENSG0000	RAB24	RAB24, me	3.416904	1.77269	0.000265	0.026978	yes
ENST0000	ENSG0000	ADRB2	adrenocep	0.352683	-1.50356	3.15E-05	0.007267	yes
ENST0000	ENSG0000	B3GALNT1	beta-1,3-N	0.002844	-8.45783	0.00039	0.033912	yes
ENST0000	ENSG0000	BUB1	BUB1 mito	0.000993	-9.97556	0.000357	0.032149	yes
ENST0000	ENSG0000	LIMS1	LIM zinc fi	3.231799	1.692338	0.000372	0.032914	yes
ENST0000	ENSG0000	TMEM266	transmeml	13.46906	3.751577	3.54E-05	0.00792	yes
ENST0000	ENSG0000	TM4SF1	transmeml	0.321496	-1.63713	8.26E-06	0.003237	yes
ENST0000	ENSG0000	TM4SF1	transmeml	0.479358	-1.06083	9.71E-05	0.014821	yes
ENST0000	ENSG0000	FRMPD4	FERM and	106.2888	6.731846	0.000229	0.024613	yes
ENST0000	ENSG0000	ALCAM	activated l	915.2475	9.838018	3.01E-05	0.007123	yes
ENST0000	ENSG0000	SERPINA6	serpin fam	489.3003	8.934576	0.000106	0.015572	yes
ENST0000	ENSG0000	CST5	cystatin D	385.095	8.58907	0.000181	0.021247	yes
ENST0000	ENSG0000	CST2	cystatin SA	41.13014	5.362124	2.16E-05	0.00583	yes
ENST0000	ENSG0000	CST1	cystatin SN	3415.709	11.73797	3.52E-06	0.00184	yes
ENST0000	ENSG0000	CST1	cystatin SN	139.3286	7.122348	0.000218	0.023812	yes
ENST0000	ENSG0000	ADORA2B	adenosine	0.460867	-1.11758	3.17E-06	0.001721	yes
ENST0000	ENSG0000	LONRF2	LON pepti	9.288857	3.215501	8.12E-05	0.013446	yes
ENST0000	ENSG0000	EMB	embigin [S	0.47467	-1.075	2.65E-05	0.00653	yes
ENST0000	ENSG0000	HAS2	hyaluronan	0.337874	-1.56544	9.22E-07	0.000869	yes
ENST0000	ENSG0000	FUT3	fucosyltrar	6.254155	2.644815	0.000389	0.033912	yes
ENST0000	ENSG0000	CDC42BPC	CDC42 bir	2.449625	1.292561	1.04E-06	0.000938	yes
ENST0000	ENSG0000	CANT1	calcium ac	2.237318	1.16177	7.84E-05	0.013135	yes
ENST0000	ENSG0000	KRT19	keratin 19	2.171252	1.118527	6.47E-07	0.000678	yes
ENST0000	ENSG0000	KRT15	keratin 15	8.263697	3.046787	0.000732	0.049342	yes
ENST0000	ENSG0000	KRT15	keratin 15	8.863277	3.14784	0.000319	0.030034	yes
ENST0000	ENSG0000	KRT15	keratin 15	8.864346	3.148014	4.78E-07	0.000592	yes
ENST0000	ENSG0000	KRT13	keratin 13	4339.085	12.08318	5.87E-07	0.000645	yes
ENST0000	ENSG0000	DSEL	dermatan	0.477418	-1.06668	1.26E-05	0.004118	yes
ENST0000	ENSG0000	LRRC8C	leucine ric	4.143139	2.050724	0.000209	0.02327	yes

ENST0000	ENSG0000	BCL2L1	BCL2 like 1	2.31251	1.20946	1.81E-05	0.00521	yes
ENST0000	ENSG0000	CLSTN1	calsyntenir	2.221993	1.151854	4.19E-06	0.002077	yes
ENST0000	ENSG0000	CXXC5	CXXC fing	2.061761	1.043877	0.000547	0.042221	yes
ENST0000	ENSG0000	CXXC5	CXXC fing	2.353938	1.235076	0.000101	0.015104	yes
ENST0000	ENSG0000	ENC1	ectoderma	0.492352	-1.02224	0.000687	0.047736	yes
ENST0000	ENSG0000	ENC1	ectoderma	0.395013	-1.34003	2.27E-05	0.005942	yes
ENST0000	ENSG0000	SYNPO	synaptopo	0.481743	-1.05366	7.81E-05	0.013114	yes
ENST0000	ENSG0000	CYCS	cytochrom	0.494938	-1.01468	5.65E-05	0.010718	yes
ENST0000	ENSG0000	SNTB1	syntrophin	742.3976	9.536048	0.000104	0.01541	yes
ENST0000	ENSG0000	SNTB1	syntrophin	9.29323	3.21618	9.48E-08	0.00025	yes
ENST0000	ENSG0000	ISG20	interferon	7.86316	2.975109	7.61E-05	0.012865	yes
ENST0000	ENSG0000	ISG20	interferon	5.750804	2.523764	2.77E-05	0.006703	yes
ENST0000	ENSG0000	ISG20	interferon	7.771152	2.958129	3.4E-07	0.000498	yes
ENST0000	ENSG0000	GTPBP2	GTP bindir	3.01384	1.591603	1.53E-05	0.004652	yes
ENST0000	ENSG0000	ZNF24	zinc finger	0.274295	-1.8662	0.000168	0.020174	yes
ENST0000	ENSG0000	MAB21L4	mab-21 lik	15.03426	3.910182	3.35E-05	0.007571	yes
ENST0000	ENSG0000	TMCC1	transmeml	3226.843	11.65591	0.000358	0.032206	yes
ENST0000	ENSG0000	RAB37	RAB37, me	959.7175	9.906466	2.19E-05	0.005845	yes
ENST0000	ENSG0000	MYEOV	myeloma c	3.867887	1.951546	0.000555	0.042547	yes
ENST0000	ENSG0000	MYEOV	myeloma c	3.501125	1.807819	0.000119	0.01679	yes
ENST0000	ENSG0000	MYEOV	myeloma c	3.841828	1.941793	2.45E-05	0.006235	yes
ENST0000	ENSG0000	GXYLT2	glucoside	0.434369	-1.20301	0.000448	0.037249	yes
ENST0000	ENSG0000	RHOD	ras homolo	4.677552	2.225754	7.17E-06	0.00296	yes
ENST0000	ENSG0000	ABLIM3	actin bindi	2.215419	1.14758	4.7E-06	0.002224	yes
ENST0000	ENSG0000	ABLIM3	actin bindi	2.20048	1.137818	4.28E-05	0.008982	yes
ENST0000	ENSG0000	GLRX	glutaredox	16.94177	4.082513	7.98E-08	0.000243	yes
ENST0000	ENSG0000	SYT12	synaptotag	15.02422	3.909218	5.92E-09	7.38E-05	yes
ENST0000	ENSG0000	SYT12	synaptotag	10.77765	3.429971	4.9E-05	0.009692	yes
ENST0000	ENSG0000	C11orf86	chromosor	3.858901	1.94819	0.000367	0.032668	yes
ENST0000	ENSG0000	SNCG	synuclein g	2.681726	1.423162	0.000339	0.03132	yes
ENST0000	ENSG0000	NABP1	nucleic aci	0.328634	-1.60545	0.000288	0.028183	yes
ENST0000	ENSG0000	HEG1	heart deve	2.155854	1.10826	0.000378	0.033255	yes
ENST0000	ENSG0000	HEG1	heart deve	2.081279	1.05747	2.55E-05	0.006392	yes
ENST0000	ENSG0000	CD34	CD34 mole	601.0443	9.231327	6.03E-05	0.011209	yes
ENST0000	ENSG0000	SH3PXD2E	SH3 and P	3.431406	1.7788	5.83E-08	0.000222	yes
ENST0000	ENSG0000	PDZK1	PDZ doma	11.37061	3.507238	0.000599	0.044296	yes
ENST0000	ENSG0000	PDZK1	PDZ doma	21.00857	4.392906	0.000565	0.042883	yes
ENST0000	ENSG0000	ATR	ATR serine	0.33132	-1.5937	0.000324	0.030397	yes
ENST0000	ENSG0000	MARCKSL1	MARCKS li	2.782849	1.476562	1.27E-07	0.000296	yes
ENST0000	ENSG0000	YPEL2	yippee like	5.673919	2.504346	1.39E-06	0.001109	yes
ENST0000	ENSG0000	DDIT3	DNA dama	2.646972	1.404343	0.000501	0.039854	yes
ENST0000	ENSG0000	TP53I11	tumor pro	0.36461	-1.45557	8.85E-05	0.013977	yes
ENST0000	ENSG0000	PROP1	PROP pair	570.2454	9.155439	7.66E-05	0.012898	yes
ENST0000	ENSG0000	CLTB	clathrin lig	2.698493	1.432154	5.01E-06	0.002327	yes
ENST0000	ENSG0000	LONRF3	LON pepti	6.081427	2.60441	0.000291	0.028305	yes
ENST0000	ENSG0000	NR2F1	nuclear re	0.329857	-1.60009	2.5E-06	0.001541	yes
ENST0000	ENSG0000	CRYBG2	crystallin b	6.170529	2.625394	3.02E-06	0.001682	yes
ENST0000	ENSG0000	CRYBG2	crystallin b	6.013597	2.588228	6.29E-05	0.011415	yes
ENST0000	ENSG0000	BNIP3	BCL2 inter	2.050326	1.035853	9.75E-05	0.014836	yes
ENST0000	ENSG0000	RIMS2	regulating	0.235383	-2.08692	1.01E-05	0.003659	yes
ENST0000	ENSG0000	SYNE3	spectrin re	7.618691	2.929543	1.91E-07	0.000368	yes
ENST0000	ENSG0000	BASP1	brain abur	0.444512	-1.1697	2.64E-05	0.006523	yes
ENST0000	ENSG0000	METRNL	meteorin l	2.809237	1.490178	1.82E-05	0.005224	yes
ENST0000	ENSG0000	METRNL	meteorin l	4.074023	2.026454	3.73E-06	0.001913	yes
ENST0000	ENSG0000	MUC20	mucin 20,	3.937377	1.977235	8.65E-05	0.013856	yes
ENST0000	ENSG0000	MUC20	mucin 20,	4.540696	2.182914	0.000283	0.027951	yes
ENST0000	ENSG0000	ACER2	alkaline ce	5.58194	2.480767	8.9E-06	0.003402	yes
ENST0000	ENSG0000	ULK1	unc-51 like	2.324674	1.217029	3.66E-05	0.008132	yes

ENST0000(	ENSG0000	RPS6KA3	ribosomal	0.004106	-7.92801	0.00056	0.042691	yes
ENST0000(	ENSG0000	MIEF2	mitochonc	4.695505	2.23128	5.16E-06	0.002381	yes
ENST0000(	ENSG0000	ACOT4	acyl-CoA t	4.117146	2.041645	2.84E-06	0.001627	yes
ENST0000(	ENSG0000	IRX3	iroquois h	2.933586	1.552665	2.17E-06	0.001442	yes
ENST0000(	ENSG0000	ALS2CL	ALS2 C-te	2.799542	1.485191	7.51E-06	0.003032	yes
ENST0000(	ENSG0000	GRAMD1C	GRAM dor	3.015977	1.592625	2.19E-05	0.005845	yes
ENST0000(	ENSG0000	MAF	MAF bZIP	104.0268	6.700811	1.26E-07	0.000296	yes
ENST0000(	ENSG0000	MAF	MAF bZIP	52.67491	5.719044	0.00014	0.018198	yes
ENST0000(	ENSG0000	KCTD12	potassium	2.076807	1.054367	2.04E-05	0.005589	yes
ENST0000(	ENSG0000	CIITA	class II ma	0.338674	-1.56203	0.000357	0.032149	yes
ENST0000(	ENSG0000	PCED1B	PC-esteras	0.326819	-1.61344	4.51E-05	0.009255	yes
ENST0000(	ENSG0000	B3GNT3	UDP-GlcN	3.623985	1.857577	1.62E-06	0.001213	yes
ENST0000(	ENSG0000	MCFD2	multiple cc	2.83346	1.502565	0.000455	0.037584	yes
ENST0000(	ENSG0000	MCFD2	multiple cc	4.777892	2.256374	0.000204	0.023015	yes
ENST0000(	ENSG0000	MCFD2	multiple cc	2.50209	1.323134	3.31E-07	0.000489	yes
ENST0000(	ENSG0000	TMEM64	transmeml	2.458853	1.297986	1.37E-05	0.004337	yes
ENST0000(	ENSG0000	CHRM4	cholinergi	30.00768	4.90726	8.97E-06	0.00342	yes
ENST0000(	ENSG0000	HIGD1A	HIG1 hypc	2.354492	1.235416	1.44E-05	0.004497	yes
ENST0000(	ENSG0000	HIGD1A	HIG1 hypc	4.260631	2.091067	0.000682	0.047501	yes
ENST0000(	ENSG0000	SOX2	SRY-box t	5.88594	2.557273	0.000591	0.043949	yes
ENST0000(	ENSG0000	TMIE	transmeml	12.61454	3.657015	0.000195	0.022298	yes
ENST0000(	ENSG0000	TNFSF15	TNF super	3.540163	1.823816	0.000454	0.037584	yes
ENST0000(	ENSG0000	SHMT2	serine hyd	2.075588	1.05352	0.000415	0.035523	yes
ENST0000(	ENSG0000	CACNB4	calcium vc	8.117051	3.020956	0.00056	0.042691	yes
ENST0000(	ENSG0000	CACNB4	calcium vc	705.8117	9.46314	7.61E-05	0.012865	yes
ENST0000(	ENSG0000	CACNB4	calcium vc	423.1481	8.725019	0.000192	0.022059	yes
ENST0000(	ENSG0000	XKRX	XK related	6.465308	2.692719	0.000217	0.023776	yes
ENST0000(	ENSG0000	CSF1R	colony stir	2.856103	1.514048	3.51E-06	0.00184	yes
ENST0000(	ENSG0000	C1orf116	chromosor	3.975332	1.991075	3.21E-07	0.00048	yes
ENST0000(	ENSG0000	C1orf116	chromosor	3.729204	1.898868	7.12E-07	0.000711	yes
ENST0000(	ENSG0000	COL18A1	collagen ty	3.982416	1.993644	1.14E-06	0.000989	yes
ENST0000(	ENSG0000	COL18A1	collagen ty	5.700678	2.511133	4.6E-05	0.009339	yes
ENST0000(	ENSG0000	CADM1	cell adhesi	15.37814	3.942809	5E-06	0.002327	yes
ENST0000(	ENSG0000	CADM1	cell adhesi	28.78909	4.84745	2E-07	0.000368	yes
ENST0000(	ENSG0000	CADM1	cell adhesi	17.87822	4.160131	6.6E-05	0.011743	yes
ENST0000(	ENSG0000	CADM1	cell adhesi	31.34987	4.970388	6.62E-09	7.38E-05	yes
ENST0000(	ENSG0000	ABAT	4-aminobu	7.171253	2.842225	1.38E-06	0.001104	yes
ENST0000(	ENSG0000	GRIN2A	glutamate	0.206294	-2.27722	0.000729	0.049284	yes
ENST0000(	ENSG0000	GTF2H2C	GTF2H2 fa	0.493474	-1.01895	0.000596	0.04413	yes
ENST0000(	ENSG0000	SFXN4	sideroflexi	0.460322	-1.11929	0.000128	0.017469	yes
ENST0000(	ENSG0000	MACC1	MET transc	2.517943	1.332246	6.33E-06	0.0027	yes
ENST0000(	ENSG0000	ALDH1A3	aldehyde c	0.210875	-2.24554	2.53E-06	0.001554	yes
ENST0000(	ENSG0000	ALDH1A3	aldehyde c	0.239686	-2.06078	1.12E-05	0.003849	yes
ENST0000(	ENSG0000	FOXO4	forkhead b	2.421445	1.275868	0.000436	0.036683	yes
ENST0000(	ENSG0000	DUSP8	dual specit	2.197201	1.135667	0.0006	0.044296	yes
ENST0000(	ENSG0000	TMEM173	transmeml	4.403312	2.138589	1.61E-07	0.000343	yes
ENST0000(	ENSG0000	SEPTIN9	septin 9 [S	5.036907	2.332538	0.000692	0.047905	yes
ENST0000(	ENSG0000	RBM33	RNA bindi	0.296874	-1.75208	0.000287	0.028085	yes
ENST0000(	ENSG0000	JAG2	jagged car	0.436357	-1.19642	0.000491	0.039345	yes
ENST0000(	ENSG0000	FMNL1	formin like	11.24329	3.490993	8.7E-05	0.013906	yes
ENST0000(	ENSG0000	MAFF	MAF bZIP	4.254697	2.089056	1.44E-05	0.004497	yes
ENST0000(	ENSG0000	MAFF	MAF bZIP	2.652605	1.40741	0.000128	0.017435	yes
ENST0000(	ENSG0000	FLRT2	fibronectir	0.18951	-2.39965	1.1E-06	0.000974	yes
ENST0000(	ENSG0000	FLRT2	fibronectir	0.118656	-3.07514	9.27E-08	0.00025	yes
ENST0000(	ENSG0000	FAM43A	family wit	8.394596	3.069461	7.31E-07	0.000721	yes
ENST0000(	ENSG0000	TCN2	transcobal	2.23036	1.157277	0.000652	0.046449	yes
ENST0000(	ENSG0000	TEDC1	tubulin ep	0.007341	-7.0899	0.000214	0.023604	yes
ENST0000(	ENSG0000	METTL7A	methyltrar	354.3986	8.469229	0.000239	0.025343	yes

ENST0000(	ENSG0000	SV2B	synaptic ve	0.00386	-8.01735	0.00052	0.04087	yes
ENST0000(	ENSG0000	ZFP36L1	ZFP36 ring	0.38234	-1.38707	9.19E-06	0.003468	yes
ENST0000(	ENSG0000	ZFP36L1	ZFP36 ring	0.468704	-1.09325	2.78E-06	0.001614	yes
ENST0000(	ENSG0000	YTHDF3	YTH N6-m	202.9781	7.66518	0.000112	0.016136	yes
ENST0000(	ENSG0000	IFIT1	interferon	2.201425	1.138438	5.62E-06	0.002522	yes
ENST0000(	ENSG0000	ADAMTSL!	ADAMTS l	3.204461	1.680082	1.21E-05	0.00406	yes
ENST0000(	ENSG0000	PCYT2	phosphate	3.324854	1.733291	1.34E-05	0.004275	yes
ENST0000(	ENSG0000	C15orf41	chromosoı	0.457608	-1.12782	0.000469	0.038333	yes
ENST0000(	ENSG0000	ZNRF1	zinc and ri	2.215329	1.147521	4.07E-05	0.008722	yes
ENST0000(	ENSG0000	ZNRF1	zinc and ri	2.447849	1.291515	0.000476	0.038607	yes
ENST0000(	ENSG0000	CYP4F3	cytochrom	224.2359	7.808873	0.000731	0.049317	yes
ENST0000(	ENSG0000	NF2	neurofibro	0.448182	-1.15784	0.000163	0.019892	yes
ENST0000(	ENSG0000	HPDL	4-hydroxy	2.020928	1.015018	0.000311	0.029511	yes
ENST0000(	ENSG0000	CYP27C1	cytochrom	0.1907	-2.39062	0.000117	0.016581	yes
ENST0000(	ENSG0000	MPPED1	metallophı	350.7519	8.454307	0.000687	0.047736	yes
ENST0000(	ENSG0000	MPPED1	metallophı	1291.517	10.33485	1.41E-05	0.004431	yes
ENST0000(	ENSG0000	HYAL3	hyaluronid	3.086587	1.626012	0.000727	0.049227	yes
ENST0000(	ENSG0000	ZNF395	zinc finger	2.628839	1.394426	4.11E-07	0.000541	yes
ENST0000(	ENSG0000	ZNF395	zinc finger	2.740667	1.454527	0.000358	0.032206	yes
ENST0000(	ENSG0000	ESPN	espin [Sou	4.431374	2.147754	0.000121	0.016907	yes
ENST0000(	ENSG0000	ESPN	espin [Sou	7.322964	2.872428	8.63E-05	0.013835	yes
ENST0000(	ENSG0000	ENTPD5	ectonucleo	0.497125	-1.00832	2.2E-05	0.005845	yes
ENST0000(	ENSG0000	MT1X	metallothi	3.104208	1.634225	5.18E-07	0.000613	yes
ENST0000(	ENSG0000	DYNC2H1	dynein cyt	0.46368	-1.1088	7.53E-05	0.012762	yes
ENST0000(	ENSG0000	TAF9B	TATA-box	0.446828	-1.16221	6.63E-05	0.011774	yes
ENST0000(	ENSG0000	KCNJ11	potassium	2.552516	1.35192	0.000541	0.041943	yes
ENST0000(	ENSG0000	PLEKHN1	pleckstrin	0.46329	-1.11001	9.3E-05	0.014471	yes
ENST0000(	ENSG0000	ISG15	ISG15 ubic	4.26493	2.092522	0.000185	0.021578	yes
ENST0000(	ENSG0000	SPRY4	sprouty RT	3.608906	1.851562	6.91E-07	0.000696	yes
ENST0000(	ENSG0000	TRPV2	transient r	2.679051	1.421722	7.24E-05	0.012456	yes
ENST0000(	ENSG0000	THSD4	thrombosp	2.148034	1.103017	5.56E-06	0.002513	yes
ENST0000(	ENSG0000	FANCM	FA comple	0.399006	-1.32552	0.000434	0.036568	yes
ENST0000(	ENSG0000	PEAR1	platelet en	459.64	8.844361	0.000627	0.045496	yes
ENST0000(	ENSG0000	ARL4C	ADP ribosy	2.095985	1.067628	2.48E-06	0.001541	yes
ENST0000(	ENSG0000	SMTNL2	smoothelir	37.35013	5.223041	4.21E-05	0.008912	yes
ENST0000(	ENSG0000	PLSCR1	phospholip	2.037415	1.02674	0.000165	0.02004	yes
ENST0000(	ENSG0000	SERPINA5	serpin fam	6.240611	2.641687	0.000347	0.031677	yes
ENST0000(	ENSG0000	SERPINA5	serpin fam	7.324732	2.872776	1.48E-05	0.004565	yes
ENST0000(	ENSG0000	NANOS1	nanos C2H	3.352905	1.745412	1.97E-05	0.005494	yes
ENST0000(	ENSG0000	DPYD	dihydropy	0.365435	-1.45231	8.34E-05	0.013571	yes
ENST0000(	ENSG0000	S100A16	S100 calciu	2.188758	1.130113	7.05E-05	0.012229	yes
ENST0000(	ENSG0000	GJB3	gap junctio	2.638312	1.399615	5.9E-06	0.002616	yes
ENST0000(	ENSG0000	GJB3	gap junctio	2.852499	1.512227	0.000152	0.019188	yes
ENST0000(	ENSG0000	MAOA	monoamir	2.944334	1.557941	3.03E-05	0.007139	yes
ENST0000(	ENSG0000	S100A14	S100 calciu	3.377309	1.755874	2.58E-06	0.001561	yes
ENST0000(	ENSG0000	KIAA0408	KIAA0408	4.309993	2.107685	0.000462	0.037973	yes
ENST0000(	ENSG0000	KIAA0408	KIAA0408	3.274163	1.711126	5.78E-06	0.002569	yes
ENST0000(	ENSG0000	PLEKHG4	pleckstrin	0.240025	-2.05874	0.000695	0.04804	yes
ENST0000(	ENSG0000	PLEKHG4	pleckstrin	0.212675	-2.23328	3.81E-05	0.008348	yes
ENST0000(	ENSG0000	FAT4	FAT atypic	3.154288	1.657314	2.27E-06	0.001472	yes
ENST0000(	ENSG0000	TMEM63A	transmeml	2.209451	1.143688	0.000169	0.020288	yes
ENST0000(	ENSG0000	GREB1	growth reg	1560.51	10.6078	8.46E-06	0.003287	yes
ENST0000(	ENSG0000	GREB1	growth reg	3.92092	1.971192	3.11E-07	0.000474	yes
ENST0000(	ENSG0000	GREB1	growth reg	342.3211	8.419206	1.47E-05	0.004544	yes
ENST0000(	ENSG0000	GREB1	growth reg	577.9554	9.174814	0.000156	0.019457	yes
ENST0000(	ENSG0000	GREB1	growth reg	93.71788	6.550252	0.000105	0.015451	yes
ENST0000(	ENSG0000	GREB1	growth reg	2703.538	11.40063	2.22E-06	0.001451	yes
ENST0000(	ENSG0000	GREB1	growth reg	66.84693	6.062789	0.000272	0.027333	yes

ENST0000	ENSG0000	NTNG2	netrin G2 [	31.5698	4.980473	7.94E-05	0.013254	yes
ENST0000	ENSG0000	NTNG2	netrin G2 [	9.412129	3.234521	6.05E-06	0.002616	yes
ENST0000	ENSG0000	LONP1	lon peptid	1737.578	10.76286	5.44E-06	0.002478	yes
ENST0000	ENSG0000	SRGAP2B	SLIT-ROB(	3.062579	1.614747	2.15E-05	0.00582	yes
ENST0000	ENSG0000	SLC6A9	solute carr	4.639735	2.214042	3.21E-07	0.00048	yes
ENST0000	ENSG0000	LAMA2	laminin su	4.080538	2.028759	0.000206	0.02315	yes
ENST0000	ENSG0000	RABL6	RAB, mem	3.329854	1.735459	0.000567	0.042991	yes
ENST0000	ENSG0000	COL27A1	collagen ty	2.142015	1.098969	1.22E-05	0.00406	yes
ENST0000	ENSG0000	GM2A	GM2 gang	3.759298	1.910463	7.28E-08	0.00024	yes
ENST0000	ENSG0000	MAML3	mastermin	3.750379	1.907036	3.03E-05	0.007139	yes
ENST0000	ENSG0000	CBWD3	COBW doi	0.49956	-1.00127	0.000379	0.033268	yes
ENST0000	ENSG0000	HCAR1	hydroxyca	0.48444	-1.04561	4.24E-05	0.008955	yes
ENST0000	ENSG0000	FLNA	filamin A [	0.256963	-1.96037	3.31E-06	0.001771	yes
ENST0000	ENSG0000	SRGAP1	SLIT-ROB(	0.493761	-1.01811	3.47E-06	0.001831	yes
ENST0000	ENSG0000	FAM3C	family with	0.439252	-1.18688	3.67E-06	0.001892	yes
ENST0000	ENSG0000	SMIM10L2	small integ	22.17098	4.470601	1.58E-05	0.004739	yes
ENST0000	ENSG0000	ACSL5	acyl-CoA s	16.60284	4.053358	1.71E-05	0.004999	yes
ENST0000	ENSG0000	ACSL5	acyl-CoA s	3.951463	1.982387	0.000124	0.017236	yes
ENST0000	ENSG0000	SERPINA1	serpin fam	8.443234	3.077796	7.87E-05	0.013169	yes
ENST0000	ENSG0000	SERPINA1	serpin fam	8.910809	3.155556	1.22E-08	8.84E-05	yes
ENST0000	ENSG0000	SERPINA1	serpin fam	7.972721	2.995072	1.29E-05	0.004191	yes
ENST0000	ENSG0000	STMN3	stathmin 3	0.403941	-1.30778	1.32E-05	0.004239	yes
ENST0000	ENSG0000	ZNF628	zinc finger	0.000818	-10.2552	1.02E-05	0.003679	yes
ENST0000	ENSG0000	ZNF628	zinc finger	2.168981	1.117017	0.000436	0.036682	yes
ENST0000	ENSG0000	SERPINB2	serpin fam	0.111718	-3.16206	5.53E-05	0.010587	yes
ENST0000	ENSG0000	DPP4	dipeptidyl	3.30838	1.726125	2.65E-06	0.001585	yes
ENST0000	ENSG0000	PSAP	prosaposir	2.083035	1.058687	4.62E-05	0.009339	yes
ENST0000	ENSG0000	PSAP	prosaposir	2.284789	1.192061	1.32E-06	0.001072	yes
ENST0000	ENSG0000	UGT2B17	UDP glucu	28.36904	4.826245	0.000119	0.016792	yes
ENST0000	ENSG0000	KIF13B	kinesin fan	2.916582	1.544278	5.55E-07	0.000636	yes
ENST0000	ENSG0000	ZNF347	zinc finger	0.484042	-1.0468	0.000577	0.043417	yes
ENST0000	ENSG0000	SIRPA	signal regu	2.136958	1.095559	2.29E-06	0.001473	yes
ENST0000	ENSG0000	SIRPA	signal regu	2.448717	1.292026	0.000485	0.039084	yes
ENST0000	ENSG0000	HIBCH	3-hydroxy	0.00148	-9.40053	6.08E-05	0.011266	yes
ENST0000	ENSG0000	PHETA1	PH domair	4.965983	2.312079	0.000611	0.044725	yes
ENST0000	ENSG0000	PHETA1	PH domair	6.423573	2.683376	0.000101	0.015104	yes
ENST0000	ENSG0000	ASPH	aspartate I	0.480303	-1.05798	5.97E-06	0.002616	yes
ENST0000	ENSG0000	MT1F	metallothi	3.278471	1.713023	0.000287	0.028085	yes
ENST0000	ENSG0000	B3GNT6	UDP-GlcN	76.89002	6.264724	1E-08	8.33E-05	yes
ENST0000	ENSG0000	PAPSS2	3'-phosph	2.727067	1.44735	3.32E-06	0.001771	yes
ENST0000	ENSG0000	FAN1	FANCD2 a	0.002213	-8.8198	0.000218	0.023815	yes
ENST0000	ENSG0000	GLMP	glycosylate	2.143332	1.099855	0.000416	0.03562	yes
ENST0000	ENSG0000	PLXNB3	plexin B3 [	2.497561	1.32052	0.000113	0.016305	yes
ENST0000	ENSG0000	APCDD1L	APC down	2.58819	1.371944	1.53E-05	0.004641	yes
ENST0000	ENSG0000	GK	glycerol ki	2.356434	1.236605	0.000681	0.047501	yes
ENST0000	ENSG0000	GK	glycerol ki	7.030082	2.813541	7.15E-06	0.00296	yes
ENST0000	ENSG0000	RUSC2	RUN and S	2.157973	1.109677	2.7E-06	0.001596	yes
ENST0000	ENSG0000	SHISA4	shisa famil	2.302263	1.203053	0.00036	0.032267	yes
ENST0000	ENSG0000	TBKBP1	TBK1 bind	3.608525	1.851409	4.86E-07	0.000597	yes
ENST0000	ENSG0000	TGM2	transgluta	8.340964	3.060214	1.65E-09	5.59E-05	yes
ENST0000	ENSG0000	RBM20	RNA bindi	8.505962	3.088474	0.00027	0.027285	yes
ENST0000	ENSG0000	TCEA3	transcripti	2.462485	1.300115	0.000728	0.049246	yes
ENST0000	ENSG0000	HLA-DRA	major hist	0.055321	-4.17602	1.11E-06	0.000975	yes
ENST0000	ENSG0000	CD177	CD177 mc	3.055327	1.611327	0.000231	0.024721	yes
ENST0000	ENSG0000	ADGRG1	adhesion (	2.342257	1.227899	6.55E-05	0.011696	yes
ENST0000	ENSG0000	MT1A	metallothi	2.360501	1.239093	1.42E-05	0.004445	yes
ENST0000	ENSG0000	KRT81	keratin 81	0.077842	-3.6833	6.66E-08	0.000235	yes
ENST0000	ENSG0000	CCDC85C	coiled-coil	2.101185	1.071203	7.68E-06	0.003067	yes

ENST0000(	ENSG0000	CCDC85C	coiled-coil	2.385061	1.254026	2.66E-06	0.001585	yes
ENST0000(	ENSG0000	KLRC3	killer cell le	300.2145	8.22985	0.00047	0.038351	yes
ENST0000(	ENSG0000	RNPS1	RNA bindi	0.002988	-8.3867	0.000554	0.042547	yes
ENST0000(	ENSG0000	ATP10A	ATPase ph	0.250302	-1.99826	3.16E-06	0.001721	yes
ENST0000(	ENSG0000	HLA-A	major histo	2.495711	1.319451	3.54E-07	0.000503	yes
ENST0000(	ENSG0000	IGHE	immunogl	3.108146	1.636054	3.06E-05	0.007153	yes
ENST0000(	ENSG0000	CGB8	chorionic g	0.08805	-3.50553	1.11E-05	0.003835	yes
ENST0000(	ENSG0000	MLLT11	MLLT11 tra	2.421119	1.275674	0.000515	0.040669	yes
ENST0000(	ENSG0000	QTRT1	queuine tR	0.475	-1.074	0.000355	0.032089	yes
ENST0000(	ENSG0000	LBH	LBH regula	5.323843	2.412468	9.53E-06	0.003541	yes
ENST0000(	ENSG0000	S1PR3	sphingosir	3.181227	1.669583	3.27E-06	0.001764	yes
ENST0000(	ENSG0000	EMP2	epithelial r	2.048934	1.034873	1.63E-06	0.001213	yes
ENST0000(	ENSG0000	IRF9	interferon	2.957337	1.564299	7.86E-07	0.000764	yes
ENST0000(	ENSG0000	ITGA1	integrin su	4.853502	2.279026	3.98E-06	0.002001	yes
ENST0000(	ENSG0000	SH3D21	SH3 doma	2.498312	1.320954	1.6E-05	0.004766	yes
ENST0000(	ENSG0000	SH3D21	SH3 doma	3.481998	1.799915	1.22E-05	0.00406	yes
ENST0000(	ENSG0000	SOGA3	SOGA fam	4.850611	2.278166	0.000136	0.01797	yes
ENST0000(	ENSG0000	SOGA3	SOGA fam	3.149491	1.655119	0.000295	0.02853	yes
ENST0000(	ENSG0000	NEURL1B	neuralized	3.417299	1.772856	9.07E-05	0.014222	yes
ENST0000(	ENSG0000	ZSWIM8	zinc finger	2.581771	1.368361	1.69E-05	0.004959	yes
ENST0000(	ENSG0000	ARHGEF28	Rho guani	2.175767	1.121524	0.000597	0.044178	yes
ENST0000(	ENSG0000	ZNF407	zinc finger	0.34522	-1.53441	0.000117	0.016637	yes
ENST0000(	ENSG0000	TENM3	teneurin tr	0.457193	-1.12912	0.000117	0.016637	yes
ENST0000(	ENSG0000	TRIM16	tripartite m	2.382636	1.252558	0.000139	0.018142	yes
ENST0000(	ENSG0000	TINCR	TINCR ubi(	3.040541	1.604328	4.66E-05	0.009371	yes
ENST0000(	ENSG0000	ZNF469	zinc finger	0.408667	-1.291	0.000375	0.033048	yes
ENST0000(	ENSG0000	SCAMP4	secretory c	3.25302	1.70178	0.00054	0.041891	yes
ENST0000(	ENSG0000	HLA-DPA1	major histo	0.078565	-3.66998	4.38E-05	0.00904	yes
ENST0000(	ENSG0000	ZNF736	zinc finger	2.833817	1.502746	6.76E-06	0.00283	yes
ENST0000(	ENSG0000	ZNF736	zinc finger	2.565231	1.359089	1.07E-06	0.000961	yes
ENST0000(	ENSG0000	CASTOR3	CASTOR fa	2.542553	1.346278	1.88E-05	0.005365	yes
ENST0000(	ENSG0000	PRR20G	proline ricl	3.002484	1.586157	0.000271	0.027306	yes
ENST0000(	ENSG0000	CFB	compleme	0.345252	-1.53428	9.33E-06	0.003504	yes
ENST0000(	ENSG0000	RTL9	retrotransp	4.750796	2.248169	5.56E-05	0.010626	yes
ENST0000(	ENSG0000	IFITM10	interferon	4.317177	2.110088	4.31E-07	0.000552	yes
ENST0000(	ENSG0000	ETV5	ETS varian	2.376454	1.24881	2.63E-05	0.006519	yes
ENST0000(	ENSG0000	TWF2	twinfilin ac	3.767407	1.913572	4.03E-06	0.00202	yes
ENST0000(	ENSG0000	FMN1	formin 1 [S	3.130757	1.646512	9.58E-06	0.003549	yes
ENST0000(	ENSG0000	FMN1	formin 1 [S	3.693071	1.884821	2.62E-07	0.000425	yes
ENST0000(	ENSG0000	TMEM158	transmeml	0.441447	-1.17969	0.000536	0.041687	yes
ENST0000(	ENSG0000	RTL1	retrotransp	0.234651	-2.09141	9.42E-05	0.014571	yes
ENST0000(	ENSG0000	AC068775	novel prot	88.03813	6.460057	0.000472	0.038404	yes
ENST0000(	ENSG0000	KCNJ18	potassium	4.708373	2.235229	0.000498	0.039652	yes
ENST0000(	ENSG0000	MMP12	matrix met	405.5343	8.66368	9.49E-06	0.003537	yes
ENST0000(	ENSG0000	MYZAP	myocardia	4.075953	2.027137	0.000108	0.015742	yes
ENST0000(	ENSG0000	OTUD7B	OTU deub	2.559417	1.355815	6.76E-07	0.00069	yes
ENST0000(	ENSG0000	RASSF5	Ras associ	3.495614	1.805546	0.000124	0.017217	yes
ENST0000(	ENSG0000	ERVV-2	endogeno	13.3119	3.734644	1.73E-08	0.000104	yes
ENST0000(	ENSG0000	ERVV-1	endogeno	13.44868	3.749393	8.2E-05	0.013478	yes
ENST0000(	ENSG0000	FAM47E-S	FAM47E-S	230.9809	7.85163	0.00059	0.043913	yes
ENST0000(	ENSG0000	LHX1	LIM home	0.385117	-1.37663	0.000559	0.042691	yes
ENST0000(	ENSG0000	CASTOR2	cytosolic a	3.560675	1.832151	6.91E-08	0.000238	yes
ENST0000(	ENSG0000	NATD1	N-acetyltra	5.790975	2.533806	1.67E-05	0.004893	yes
ENST0000(	ENSG0000	CCL15	C-C motif	398.4372	8.638208	0.000211	0.023396	yes
ENST0000(	ENSG0000	AC007906	novel prot	5.15155	2.365007	0.0003	0.028879	yes

Regulate	oeNC_1	oeNC_2	oeNC_3	oeESR2_1	oeESR2_2	oeESR2_3	oeNC	oeESR2
up	4.06	4.34	4.32	8.73	9.38	8.53	4.24	8.88
up	1.43	1.75	1.84	3.83	3.81	4.22	1.673333	3.953333
up	56.4	63.41	61.23	107.85	130.96	106.94	60.34667	115.25
up	1.14	1.29	1.09	4.38	2.91	3.89	1.173333	3.726667
up	12.42	16.24	14.29	34.72	38.72	36.87	14.31667	36.77
up	0.44	0.42	0.41	2.97	3.47	3.44	0.423333	3.293333
up	1.04	0.71	1.01	3.92	4.65	3.93	0.92	4.166667
up	2.2	2.9	2.24	5.47	4.27	4.26	2.446667	4.666667
up	1.91	1.78	1.75	6.58	8.06	6.62	1.813333	7.086667
up	1.19	0.91	1.05	2.69	2.75	2.44	1.05	2.626667
up	0.95	0.57	0.8	3.26	5.93	2.35	0.773333	3.846667
up	6.39	7.75	7	14.15	13.44	13.73	7.046667	13.77333
up	0.38	0.5	0.43	1.11	1.22	1.39	0.436667	1.24
down	5.04	4.23	2.99	0.37	0.7	0.39	4.086667	0.486667
down	8.91	10.64	11.85	2.44	3.25	2.41	10.46667	2.7
up	1.22	1.33	1.4	2.36	2.31	2.74	1.316667	2.47
up	20.91	18.48	17.06	44.39	38.89	55.12	18.81667	46.13333
up	87.32	79.76	96.7	204.64	306.14	168.72	87.92667	226.5
up	6	9.81	7.42	21.3	21.98	19.78	7.743333	21.02
up	1.5	2.13	1.76	3.33	4.09	5.37	1.796667	4.263333
up	5.77	5.97	5.83	11.3	12.74	11.12	5.856667	11.72
up	3.28	3.61	3.26	93.48	102.31	89.13	3.383333	94.97333
up	2.46	2.03	2.31	46.91	29.65	48.43	2.266667	41.66333
up	0	0	0	3.56	5.54	3.81	0	4.303333
up	0.14	0.1	0.22	0.73	1.48	0.86	0.153333	1.023333
up	5.4	6.09	6.09	12.27	15.92	9.3	5.86	12.49667
up	1.76	1.98	2.2	3.8	3.52	3.83	1.98	3.716667
up	1.38	2.46	1.72	5.62	7.61	5.42	1.853333	6.216667
up	1.58	1.41	1.71	3.78	3.91	3.73	1.566667	3.806667
down	18.96	16.7	17.99	7.67	8.64	8.59	17.88333	8.3
up	8.07	8.89	8.77	20.03	22.87	20.22	8.576667	21.04
up	1.98	2.25	2.21	6.76	4.93	7.54	2.146667	6.41
up	12.34	11.32	12.74	29.81	38.98	29.64	12.13333	32.81
down	62.5	54.46	58.93	11.26	16.6	11.75	58.63	13.20333
down	2.41	2.47	2.58	0	0.07	0	2.486667	0.023333
up	0.76	0.83	1.06	2.53	1.86	2.78	0.883333	2.39
up	1.75	1.95	1.97	3.42	2.92	4.27	1.89	3.536667
down	7.07	6.8	7.76	2.16	2.71	2.74	7.21	2.536667
up	0.32	0.27	0.23	1.03	1.19	1.23	0.273333	1.15
up	1.81	2.37	1.35	5.37	4.09	6.7	1.843333	5.386667
up	65.79	71.73	65.5	152.13	117.95	150.61	67.67333	140.23
down	0.39	0.14	0.33	0	0	0	0.286667	0
up	2.87	2.74	2.32	4.71	4.5	5.73	2.643333	4.98
up	0	0	0	0.4	0.42	0.42	0	0.413333
down	1.91	1.58	1.75	0.36	0.51	0.47	1.746667	0.446667
down	2	1.71	1.88	0.6	0.84	0.67	1.863333	0.703333
up	0.87	0.94	1.09	3.8	3.35	4.04	0.966667	3.73
up	0.64	1.22	2.18	7.61	9.66	6.6	1.346667	7.956667
up	1.03	0.97	1.03	2.03	1.65	2.58	1.01	2.086667
up	14.72	14.89	14.99	30.77	30.38	29.93	14.86667	30.36
up	7.65	6.7	7.1	19.77	23.5	19.12	7.15	20.79667
up	1.76	2.35	2.18	13.66	14.33	13.5	2.096667	13.83
up	1.13	1.07	1.14	2.24	2.69	2.18	1.113333	2.37
up	0.3	0.94	0.52	3.28	3.11	2.6	0.586667	2.996667
up	0.31	0.58	0.62	1.8	1.48	1.87	0.503333	1.716667

up	0.5	0.23	0.62	2.31	2.31	2.3	0.45	2.306667
up	0.47	0.46	0.3	1.97	1.68	2.01	0.41	1.886667
up	17.25	16.84	17.41	33.82	34.27	33.77	17.16667	33.95333
down	1.93	1.67	2.02	0.75	0.68	0.75	1.873333	0.726667
up	0.01	0.01	0.02	0.7	0.56	0.63	0.013333	0.63
up	0.08	0.1	0.1	1.42	1.11	0.57	0.093333	1.033333
up	7.45	7.82	7.52	15.51	12.29	14.73	7.596667	14.17667
up	20.46	19.93	21.18	41.67	55.02	44.23	20.52333	46.97333
up	5.66	4.82	6.01	31.55	36.51	33.76	5.496667	33.94
up	1.99	2.6	2	6.22	5.27	5.19	2.196667	5.56
down	25.92	29.8	27.08	9.51	14.79	10.57	27.6	11.62333
up	38.79	55.25	41.71	85.14	95.06	88.64	45.25	89.61333
up	2.29	2.79	2.25	5.29	5.21	5.27	2.443333	5.256667
up	22.08	25.43	21.87	47.94	40.98	48.66	23.12667	45.86
up	0	0	0	0.14	0.23	0.18	0	0.183333
up	5.06	8.2	6.25	43.68	35.21	47.44	6.503333	42.11
up	6.82	6.52	6.85	12.88	16.95	14.71	6.73	14.84667
down	6.82	8.82	7.61	3.83	2.45	3.51	7.75	3.263333
down	0.38	0.53	0.33	0	0	0	0.413333	0
up	1.94	2.14	2.4	5.41	4.69	6.1	2.16	5.4
up	5.38	7.2	5.86	14.19	12.76	14.18	6.146667	13.71
up	3.68	2.82	2.37	6.06	6.95	5.11	2.956667	6.04
down	5.59	5.34	5.95	2.07	2.39	1.44	5.626667	1.966667
up	6.87	7.6	7.22	27.04	26.86	25.95	7.23	26.61667
up	0	0	0	0.37	0.92	0.21	0	0.5
up	1.44	1.57	1.47	3.68	3.06	4.08	1.493333	3.606667
up	1.25	1.6	1.33	7.08	7.47	7.56	1.393333	7.37
down	7.25	7.51	6.72	3.24	2.42	3.22	7.16	2.96
up	7.28	9.64	7.71	15.93	13.23	19.43	8.21	16.19667
up	4.31	4.49	4.42	7.62	8.8	8.62	4.406667	8.346667
up	0.47	0.79	0.64	3.28	2.87	2.99	0.633333	3.046667
up	6.94	9.12	6.2	12.97	14.67	14.71	7.42	14.11667
down	5.06	5.37	4.66	2.31	2.52	1.79	5.03	2.206667
up	4.54	4.1	4.49	10.48	10.73	10.89	4.376667	10.7
up	6.26	6.62	6.36	10.92	13.31	11.65	6.413333	11.96
up	2.93	2.4	2.58	6.82	6.15	6.95	2.636667	6.64
up	0.15	0.18	0.17	0.39	0.48	0.36	0.166667	0.41
up	2.07	1.83	1.79	4	4.05	4.43	1.896667	4.16
up	0	0	0	0.43	0.86	1.01	0	0.766667
up	2.1	2.03	2.23	5.37	5.19	5.58	2.12	5.38
down	1.78	1.55	1.76	0.58	0.66	0.61	1.696667	0.616667
down	1.08	1.19	1.2	0.26	0.35	0.3	1.156667	0.303333
up	6.42	6.08	5.78	23.47	22.56	24.61	6.093333	23.54667
up	7.23	9.36	9.21	30.41	38.28	29.01	8.6	32.56667
down	2.82	0.91	1.15	0	0	0	1.626667	0
up	1.4	1.42	1.39	4.68	4.61	4.79	1.403333	4.693333
down	4.29	5.3	5.17	1.81	1.91	1.99	4.92	1.903333
up	0.97	1.88	1.61	5.3	5.31	4.81	1.486667	5.14
up	10.28	11.16	10.45	21.5	23.7	21.18	10.63	22.12667
down	5.2	4.61	4.43	1.45	2.17	1.77	4.746667	1.796667
up	0	0	0	40.24	52.16	38.57	0	43.65667
up	4.05	3.97	4.15	9.52	10.83	10.33	4.056667	10.22667
up	19.98	20.04	19.71	58.1	60.22	55.88	19.91	58.06667
up	0	0	0.01	1.22	1.18	1.13	0.003333	1.176667
up	0.68	0.63	0.71	6.6	6.68	6.76	0.673333	6.68
down	1.38	0.87	1.51	0.38	0.35	0.47	1.253333	0.4
up	10.36	11.92	12.89	24.02	23.71	21.39	11.72333	23.04
up	0.89	1.39	0.58	2.86	4.04	4.13	0.953333	3.676667

up	3.22	3.37	3.86	6.5	8.4	7.46	3.483333	7.453333
up	0	0	0	0.12	0.26	0.16	0	0.18
up	1.33	1.01	1.49	3.04	4.22	2.85	1.276667	3.37
up	5.72	5.48	5.77	10.42	10.93	11.06	5.656667	10.80333
up	9.78	13.95	9.86	24.5	18.45	27.68	11.19667	23.54333
up	22.22	20.93	22.42	43.31	58.78	43.31	21.85667	48.46667
down	0.22	0.74	0.41	0	0	0	0.456667	0
up	2.34	2.9	2.62	18.86	19.59	19.1	2.62	19.18333
down	1.31	0.91	1.04	0.12	0.13	0.11	1.086667	0.12
down	54.05	46.2	54.6	20.79	29.51	21.39	51.61667	23.89667
down	1.88	1.66	1.7	0.31	0.46	0.37	1.746667	0.38
down	0.44	0.95	0.82	0	0	0	0.736667	0
up	0.31	0.31	0.29	2.13	2.66	2.05	0.303333	2.28
down	8.37	6.36	7.47	3.33	3.63	3.13	7.4	3.363333
up	12.22	13.36	12.96	23.88	26.63	23.38	12.84667	24.63
down	12.69	12	11.87	3.48	4.41	4.39	12.18667	4.093333
up	0.82	0.85	0.86	2.34	2.16	2.15	0.843333	2.216667
up	1.64	1.69	1.15	3.75	5.57	4.46	1.493333	4.593333
up	0.04	0.06	0.05	0.36	0.37	0.39	0.05	0.373333
up	0.67	0.6	0.64	1.36	1.3	1.42	0.636667	1.36
up	14.82	14.31	15.5	26.08	33.84	26.76	14.87667	28.89333
up	1.96	3.39	2.23	14.59	11.6	15.6	2.526667	13.93
up	2.35	3.96	2.92	6.51	8.22	6.77	3.076667	7.166667
up	6.04	7.02	4.61	12.04	10.49	14.06	5.89	12.19667
up	1.37	1.35	1.5	6.38	4.68	6.54	1.406667	5.866667
up	2.12	2.46	1.6	5.92	6.81	4.74	2.06	5.823333
up	2.14	2.68	1.68	6.36	7.99	6.79	2.166667	7.046667
up	2.7	3.42	2.64	9.84	9.34	9.23	2.92	9.47
up	2.4	2.56	2.72	6.33	7.5	6.93	2.56	6.92
up	0.47	0.75	0.6	4.88	4.21	6.22	0.606667	5.103333
up	8.17	9.21	8.02	16.51	16.35	15.4	8.466667	16.08667
up	3.74	3.82	3.78	29.39	29.19	30.05	3.78	29.54333
up	8.69	7.58	8	15.97	17.7	16.04	8.09	16.57
up	5.75	5.73	6.04	12.79	12.31	13.14	5.84	12.74667
up	1.8	2.69	2.78	7.83	8.26	6.17	2.423333	7.42
up	1.38	1.56	1.64	3.29	3.49	4.04	1.526667	3.606667
up	1.36	1.87	1.55	3.5	3.28	3.31	1.593333	3.363333
up	2.92	6.75	7.03	20.59	20.89	20.22	5.566667	20.56667
down	7.95	5.98	8.07	2.12	3.23	1.56	7.333333	2.303333
up	0.84	0.76	1.12	4.09	2.85	3.96	0.906667	3.633333
up	2.42	2.98	3.16	14.25	10.63	16.52	2.853333	13.8
up	1.39	1.34	0.98	3.69	3.88	3.13	1.236667	3.566667
down	16.15	22.36	20.07	5.91	5.48	5.71	19.52667	5.7
up	27.39	28.99	28.06	54.84	52.43	57.84	28.14667	55.03667
up	4.08	3.49	5.68	11.8	10.12	15.9	4.416667	12.60667
up	14.45	16.14	14.77	28.86	32.43	26.7	15.12	29.33
up	11.7	11.85	10.6	27.25	24.52	27.34	11.38333	26.37
up	24.13	24.24	24.3	48.88	53.1	50.3	24.22333	50.76
up	8.06	9.85	8.3	19.7	17.88	19.59	8.736667	19.05667
up	6.27	7.86	7.33	20.5	20.34	21.32	7.153333	20.72
up	3.01	3.48	3.38	10.94	10.29	12.02	3.29	11.08333
up	0.31	0.4	0.29	4.21	4.51	4.73	0.333333	4.483333
up	0.5	0.62	0.68	1.52	2.44	1.86	0.6	1.94
up	12.73	14.46	13.66	25.81	25.53	25.34	13.61667	25.56
up	0.41	0.38	0.51	1.22	1.86	1.1	0.433333	1.393333
up	7.56	7.41	7.66	14.81	13.51	13.98	7.543333	14.1
up	0.81	0.74	0.62	3.02	2.81	2.88	0.723333	2.903333
up	0.35	0.48	0.38	1.39	1.1	1.54	0.403333	1.343333

up	7.57	7.82	7.28	15.55	19.02	12.71	7.556667	15.76
up	0.1	0.12	0.13	1.63	1.6	1.89	0.116667	1.706667
up	22.42	20.07	21.59	42.67	40.66	36.98	21.36	40.10333
up	3.47	3.75	3.53	7.39	6.42	7.9	3.583333	7.236667
up	0.97	0.81	1.06	3.83	3.77	3.67	0.946667	3.756667
down	1.41	1.16	1.55	0.28	0.6	0.46	1.373333	0.446667
down	17.87	19.07	23.66	7.07	8.53	9.24	20.2	8.28
down	2.36	2.23	3.35	0.43	0.92	0.66	2.646667	0.67
up	8.06	8.53	10.27	20.73	26.82	16.32	8.953333	21.29
up	2.39	2.99	2.78	18.73	13.29	13.75	2.72	15.25667
up	12.2	13.11	13.3	44.64	51.98	49.11	12.87	48.57667
up	0	0	0	0.24	0.12	0.29	0	0.216667
up	1.5	1.37	1.38	2.61	2.76	2.85	1.416667	2.74
up	0	0	0	0.06	0.24	0.12	0	0.14
up	1.07	1.47	0.75	5.14	6.07	7.17	1.096667	6.126667
up	1.38	0.75	0.49	4.37	4.77	3.16	0.873333	4.1
down	8.88	11.62	10.48	3.57	3.62	3.59	10.32667	3.593333
up	0.09	0.06	0.05	1.72	1.89	1.47	0.066667	1.693333
up	1	0.5	0.76	3.65	4.8	4.15	0.753333	4.2
up	119.38	123.34	117.99	384.94	358.78	379.23	120.2367	374.3167
up	0	0	0	56.34	65.89	56.82	0	59.68333
up	2.56	3.34	2.82	7.34	8.09	7.8	2.906667	7.743333
up	5.6	4.62	6.11	11.3	13.66	12.5	5.443333	12.48667
up	4.17	13.28	7.65	40.7	40.11	42.86	8.366667	41.22333
up	42.29	38.38	43.45	89	92.94	99.31	41.37333	93.75
up	1.96	2.53	2.23	4.38	4.35	4.52	2.24	4.416667
up	0	0	0	0.86	0.33	1.63	0	0.94
up	0	0	0	0.93	1.8	0.81	0	1.18
up	6.03	5.58	6.04	11.61	10.66	10.98	5.883333	11.08333
up	2.78	3.65	2.43	6.45	5.9	5.72	2.953333	6.023333
up	3.52	3.3	3.56	5.96	6.22	8.21	3.46	6.796667
up	2.02	2.06	1.65	4.86	5.11	3.97	1.91	4.646667
up	2.28	2.64	2.41	5.11	5.18	5.56	2.443333	5.283333
down	0.6	0.57	0.68	0.14	0.18	0.13	0.616667	0.15
down	8.86	9.65	8.99	2.57	3.07	2.52	9.166667	2.72
up	7.88	11.72	8.87	19.44	19.42	20.03	9.49	19.63
down	2.64	3.15	3.21	1.12	1.32	1.37	3	1.27
up	92.65	101.82	99.24	298.42	335.9	281.7	97.90333	305.34
up	15.61	12	5.57	164.76	72.82	186.44	11.06	141.34
down	3.1	2.33	3.21	1.08	0.94	0.95	2.88	0.99
down	29.02	26.92	27.41	8.5	9.39	9.34	27.78333	9.076667
up	0.59	0.39	0.41	4.85	4.6	3.93	0.463333	4.46
up	0.5	0.73	0.68	1.9	1.82	3.05	0.636667	2.256667
up	2.05	1.93	2.1	6.95	12.11	9.48	2.026667	9.513333
down	0.53	0.68	0.55	0.13	0.11	0.1	0.586667	0.113333
down	0.64	0.68	0.54	0.1	0.07	0.13	0.62	0.1
up	4.46	5.66	4.37	10.39	12.77	8.57	4.83	10.57667
up	1.02	1.23	0.75	2.52	2.78	2.56	1	2.62
up	2.07	3.47	2.3	7.25	5.26	5.63	2.613333	6.046667
up	0.87	0.77	0.91	1.74	1.91	1.97	0.85	1.873333
down	26.33	22.86	24.05	9.3	9.19	10.84	24.41333	9.776667
up	0.14	0.13	0.19	0.62	0.42	0.58	0.153333	0.54
up	0.71	0.83	1.09	2.24	2.33	2.31	0.876667	2.293333
down	105.39	99.49	112.12	43.53	32.31	54.16	105.6667	43.33333
down	105.2	100.38	102.1	44.58	45.56	48.98	102.56	46.37333
up	0.61	1.28	1.25	5.32	3.24	4.92	1.046667	4.493333
up	6.32	7.03	6.49	15.18	14.97	14.55	6.613333	14.9
up	3.5	3.95	4.09	8.56	7.67	9.06	3.846667	8.43

down	148.36	157.66	151.53	25.56	25.92	27.9	152.5167	26.46
up	1.03	0.94	0.97	3.22	3.83	3.26	0.98	3.436667
up	0.75	0.64	0.67	2.58	3.46	3.82	0.686667	3.286667
up	11.05	12.91	10.61	23.35	17.79	25.37	11.52333	22.17
up	10.18	10.71	10.72	20.56	23.19	20.03	10.53667	21.26
up	2.21	2.66	2.11	4.44	4.04	4.66	2.326667	4.38
up	2.62	1.78	2.29	6.96	5.23	5.94	2.23	6.043333
up	0	0	0	0.07	0.08	0.05	0	0.066667
up	1.8	2.14	2.35	5.15	5.12	5.41	2.096667	5.226667
up	0	0	0	0.59	0.77	1.35	0	0.903333
up	3.77	4.34	3.69	10.2	9.81	10.63	3.933333	10.21333
up	0.39	0.55	0.41	1.66	2.13	1.77	0.45	1.853333
up	0.38	0.21	0.31	3.17	2.55	2.82	0.3	2.846667
up	3.61	3.39	3.78	6.76	7.44	6.67	3.593333	6.956667
down	6.13	6.1	5.9	1.17	1.49	1.33	6.043333	1.33
up	3.15	3.75	3.07	11.9	12.45	12.92	3.323333	12.42333
up	11.05	14.9	13.24	53.68	43.22	52.95	13.06333	49.95
up	28.34	28.19	29.02	102.33	127.9	97.7	28.51667	109.31
up	10.35	11.73	10.69	34.52	34.25	36.09	10.92333	34.95333
down	7.22	5.23	5.79	1.61	1.26	1.46	6.08	1.443333
up	7	6.1	9.88	25.72	38.94	24.44	7.66	29.7
up	2.3	1.87	1.72	7.55	8.05	8.44	1.963333	8.013333
up	0.63	0.62	0.57	1.92	1.55	1.52	0.606667	1.663333
up	20.46	16.41	18.71	34.75	37.99	36.19	18.52667	36.31
up	2.96	3.16	5.06	8.34	12.78	11.02	3.726667	10.71333
up	0.6	0.9	0.88	2.62	3.36	2.8	0.793333	2.926667
up	8.98	9.26	7.68	25.72	16.82	23.77	8.64	22.10333
down	17.05	19.16	17.47	7.62	8.24	8.03	17.89333	7.963333
up	55.79	57.1	55.87	125.06	129.76	127.85	56.25333	127.5567
down	2.72	2.53	3.07	0.81	0.96	0.94	2.773333	0.903333
down	9.7	6.27	9	2.99	3.76	3.42	8.323333	3.39
up	49.07	62.24	55.28	594.33	663.72	582.22	55.53	613.4233
up	4.21	4.2	4.85	17.9	14.65	18.71	4.42	17.08667
up	3.35	4.73	2.86	12.2	9.18	12.19	3.646667	11.19
down	16.19	14.81	16.29	6.36	7.62	6.6	15.76333	6.86
up	0	0	0.01	0.24	0.25	0.23	0.003333	0.24
up	1.21	1.37	1.48	4.78	5.32	4.61	1.353333	4.903333
up	0.2	0.15	0.06	3.57	3.17	4.2	0.136667	3.646667
up	0.16	0.25	0.13	10.12	9.82	11.31	0.18	10.41667
up	0	0	0	1.26	2.12	0.4	0	1.26
up	0	0	0	1.85	1.88	2.48	0	2.07
up	0.53	0.43	0.41	3.33	3.42	2.29	0.456667	3.013333
up	0.72	0.73	0.62	1.65	2.69	3.32	0.69	2.553333
up	2.03	2.39	2.45	8.17	8.51	8.25	2.29	8.31
up	1.68	1.9	1.37	3.96	3.58	3.96	1.65	3.833333
up	8.47	9.31	8.64	18.39	17.57	17.83	8.806667	17.93
down	1.5	2.78	1.99	0.29	0.6	0.25	2.09	0.38
down	1.96	2.04	2.23	0.46	0.42	0.55	2.076667	0.476667
up	2.57	2.56	2.74	5.49	5.46	6.22	2.623333	5.723333
up	4.67	5.38	4.74	13.36	18.14	13.76	4.93	15.08667
up	1.3	1.85	0.93	11.34	14.94	9.2	1.36	11.82667
up	3.57	3.8	3.9	9.72	10.11	9.38	3.756667	9.736667
up	1.27	1.35	1.29	2.29	2.89	2.86	1.303333	2.68
down	6.31	6.12	6.65	1.7	3.11	2.16	6.36	2.323333
down	2.91	1.81	1.98	0.47	0.34	0.31	2.233333	0.373333
up	0.05	0.04	0.03	0.62	1.42	1.01	0.04	1.016667
down	2.96	2.38	2.69	1.02	1.32	0.94	2.676667	1.093333
up	19.08	18.64	18.1	35.02	31.2	38.33	18.60667	34.85

up	0.03	0.04	0.03	0.13	0.15	0.14	0.033333	0.14
up	0.84	0.88	1.33	4.97	4.1	3.58	1.016667	4.216667
up	0	0	0	0.28	0.21	0.27	0	0.253333
down	0.71	0.57	0.67	0.21	0.26	0.28	0.65	0.25
up	2.61	4.28	1.88	8.55	9.9	8.13	2.923333	8.86
up	15.14	12.36	13.72	23.56	35	21.74	13.74	26.76667
up	0.61	0.46	0.57	3.16	2.58	2.98	0.546667	2.906667
up	3.66	3.77	3.86	11.78	15.84	12.88	3.763333	13.5
up	13.64	16.83	15.98	34.21	37.27	28.23	15.48333	33.23667
up	75.73	79.32	78.96	145.18	178.61	140.73	78.00333	154.84
up	1.4	2.1	1.22	4.54	4.73	5.94	1.573333	5.07
up	0	0	0	0.62	0.81	0.62	0	0.683333
up	0.7	1.06	1.11	1.8	2.19	1.93	0.956667	1.973333
up	1.09	1.16	1.07	5.02	5.31	5.41	1.106667	5.246667
up	0	0	0	0.41	0.49	0.57	0	0.49
down	11.38	10.17	11.1	4.89	5.55	4.23	10.88333	4.89
down	2.39	1.83	1.99	0.65	0.59	0.59	2.07	0.61
down	4.08	3.89	4.38	0.88	1.89	1.34	4.116667	1.37
down	39.16	42.23	42.6	14.61	14.65	12.25	41.33	13.83667
down	16.31	14.52	14.71	5.5	4.4	5.06	15.18	4.986667
down	72.43	66.31	67.49	13.86	22.69	19.04	68.74333	18.53
down	45.16	38.28	44.18	11.82	11	14.04	42.54	12.28667
down	17.71	19.19	15.41	5.26	4.43	4.81	17.43667	4.833333
down	25.84	24.32	24.35	8.36	8.04	8.65	24.83667	8.35
down	0.75	0.89	0.62	0.12	0.16	0.17	0.753333	0.15
up	2.07	2.44	2.03	5.73	5.96	5.71	2.18	5.8
up	13.79	12.45	12.5	46.69	45.09	53.94	12.91333	48.57333
up	14.66	15.15	18.18	31.59	39.42	31.13	15.99667	34.04667
up	3.93	3.97	3.76	12.3	13.08	12.58	3.886667	12.65333
up	1.37	1.26	1.15	6.77	7.09	7.14	1.26	7
up	2.11	3.43	2.52	9.57	11.65	8.08	2.686667	9.766667
down	2.54	2.38	2.49	0.81	0.93	0.74	2.47	0.826667
down	1.95	1.89	2.01	0.61	0.71	0.81	1.95	0.71
down	2.96	2.51	2.91	1.19	1.27	1.24	2.793333	1.233333
up	0.29	0.29	0.24	1.69	1.42	1.85	0.273333	1.653333
up	27.03	27.19	25.99	88.46	77.64	90.3	26.73667	85.46667
up	7.41	8.6	8.41	26.96	25.25	26.3	8.14	26.17
up	0.11	0.14	0.11	0.25	0.27	0.32	0.12	0.28
up	0.95	0.82	1.15	1.94	2.12	1.93	0.973333	1.996667
down	1.78	1.81	1.04	0.36	0.22	0.31	1.543333	0.296667
up	0.48	1.59	1.57	14.39	14.2	15.15	1.213333	14.58
up	1.7	0.8	0.71	10.69	17.3	12.55	1.07	13.51333
up	3.68	3.45	3.88	32.56	42.19	36.03	3.67	36.92667
up	7	8.1	7.54	18.7	16.49	20.7	7.546667	18.63
up	3.69	2.27	3.07	7.99	7.62	8.89	3.01	8.166667
up	0.59	0.7	0.67	3.31	3.27	3.27	0.653333	3.283333
up	0.29	0.24	0.34	1.13	1.05	0.97	0.29	1.05
up	20.22	26.05	23.35	64.88	67.13	61.89	23.20667	64.63333
down	1.26	1.05	1.2	0.46	0.47	0.45	1.17	0.46
down	8.83	6.67	9.08	1.21	1.11	1.6	8.193333	1.306667
down	4.18	4.49	4.17	0.89	0.97	0.9	4.28	0.92
down	2.69	3.17	3.65	0.66	0.82	0.6	3.17	0.693333
down	2.14	2.6	2.52	0.63	0.61	0.61	2.42	0.616667
down	5.54	7.18	5.26	2.46	3.06	2.77	5.993333	2.763333
down	40.42	34.83	41.7	20.7	14.7	15.91	38.98333	17.10333
up	5.79	6.48	6.24	12.34	12.73	13.05	6.17	12.70667
down	0.7	0.3	0.78	0	0	0	0.593333	0
up	10.2	8	12.68	26.96	24.17	29.15	10.29333	26.76

down	4.7	2.97	3.46	1.03	1.16	1.31	3.71	1.166667
up	0.23	0.14	0.19	0.71	0.92	0.95	0.186667	0.86
up	1.99	1.88	2	4.82	4.69	4.16	1.956667	4.556667
up	3.74	3.47	4.06	8.26	7.96	8.43	3.756667	8.216667
up	4.2	4.53	3.81	11.91	9.83	13.41	4.18	11.71667
up	4.23	3.48	4.81	9.38	11.53	11.52	4.173333	10.81
up	0.18	0.1	0.32	1.29	3.05	2.09	0.2	2.143333
down	14.81	10.76	12.91	2.65	3.06	2.58	12.82667	2.763333
down	649.48	657.16	658.86	213.08	235.25	212.33	655.1667	220.22
down	13.54	12.77	13.05	5.18	5.51	5.23	13.12	5.306667
up	0.3	0.51	0.47	2.1	2.19	1.79	0.426667	2.026667
up	3.55	3.4	3.17	13.42	13.34	13.41	3.373333	13.39
up	0.95	0.97	1.87	3.75	7.66	4.93	1.263333	5.446667
up	0.17	0.24	0.23	0.81	0.73	0.93	0.213333	0.823333
down	0.7	0.44	0.5	0	0	0	0.546667	0
up	0.41	0.17	0.5	2.38	3.06	2.88	0.36	2.773333
up	2.27	6.46	3.57	15.45	16.38	17.45	4.1	16.42667
up	3.39	4.2	4.14	11.26	11.44	10.96	3.91	11.22
down	8.22	6.31	7.3	2.77	3.03	3.17	7.276667	2.99
up	1.36	1.79	1.96	5.03	4.32	4.09	1.703333	4.48
up	7.49	8.36	7.7	16.24	15.86	15.75	7.85	15.95
up	8.83	9.07	10.23	18.38	23.14	14.69	9.376667	18.73667
down	14.26	13.13	13.84	5.59	6.2	5.69	13.74333	5.826667
up	3.2	3.16	3.03	7.04	6.71	6.61	3.13	6.786667
up	34.4	32.88	34.41	64.94	79.62	63.76	33.89667	69.44
up	2.26	2.73	2.42	5.33	5.26	5.2	2.47	5.263333
up	4.17	4.33	4.26	8.67	7.78	8.84	4.253333	8.43
up	0.23	0.29	0.12	2.2	2.12	1.7	0.213333	2.006667
up	1.27	1.62	1.24	2.6	3.5	2.44	1.376667	2.846667
up	0.03	0.02	0.03	0.34	0.29	0.25	0.026667	0.293333
up	2.24	2.56	1.78	5.43	5.7	6.92	2.193333	6.016667
up	3.58	3.32	3.37	8.59	12.12	9.21	3.423333	9.973333
up	2.4	2.57	3.23	7.7	12.74	5.71	2.733333	8.716667
up	5.1	6.74	4.62	25.36	17.61	24.42	5.486667	22.46333
up	34.41	27.22	31.79	105.33	124.08	111.15	31.14	113.52
up	12.79	10.93	11.94	22.03	24.14	27.25	11.88667	24.47333
up	11.92	11.8	12.39	23.28	25.67	23.81	12.03667	24.25333
down	4.46	3.63	4.19	1.03	1.51	0.92	4.093333	1.153333
down	2.35	2.71	2.67	0.76	0.8	0.51	2.576667	0.69
up	0.3	0.37	0.43	1.25	1.17	1.63	0.366667	1.35
up	1.01	1.79	1.37	11.9	15.22	8.33	1.39	11.81667
up	5.35	5.39	5.32	43.59	54.47	41.34	5.353333	46.46667
up	1.39	1.75	1.43	3.78	6.18	4.03	1.523333	4.663333
up	1.05	0.48	0.77	3.01	3.05	3.12	0.766667	3.06
up	0	0	0	1.47	2.36	2.18	0	2.003333
up	3.2	2.8	3.16	7.65	10.99	8.06	3.053333	8.9
up	22.46	16.3	19.72	73.49	109.23	74.46	19.49333	85.72667
up	5.26	5.77	6.28	19.79	35.13	17.34	5.77	24.08667
up	7.54	8.43	7.52	18.98	21.58	19.75	7.83	20.10333
up	7.29	7.02	6.67	16.87	14.35	15.02	6.993333	15.41333
up	0.82	0.89	0.72	2.97	2.92	2.5	0.81	2.796667
up	5.96	5.29	5.82	19.15	19.63	18.83	5.69	19.20333
down	0.92	0.4	0.87	0	0	0	0.73	0
up	0	0	0	0.68	1.37	0.57	0	0.873333
up	2.71	2.49	2.57	5.28	5.66	4.93	2.59	5.29
up	0.02	0	0.01	3.21	2.66	3	0.01	2.956667
up	0	0	0	5.73	4.73	5.79	0	5.416667
up	1.45	2.38	3.16	12.35	12.85	6.74	2.33	10.64667

up	0.95	0.94	1.35	3.69	4.72	5.05	1.08	4.486667
up	59.34	51.72	50.54	178.96	176.07	193.28	53.86667	182.77
up	2.91	3.32	3.16	5.94	5.71	5.9	3.13	5.85
down	15.48	18.91	18.61	5.15	7.58	5.12	17.66667	5.95
up	2.41	2.49	2.93	14.38	26.17	13.33	2.61	17.96
up	3.53	3.93	3.97	24.66	26.69	24.5	3.81	25.28333
up	0.28	0.3	0.31	1.98	2.28	2.15	0.296667	2.136667
up	0	0	0	0.91	1.86	2.16	0	1.643333
up	4.94	4.82	5.17	15.05	20.28	15.15	4.976667	16.82667
up	0.04	0.04	0.03	0.64	0.75	0.48	0.036667	0.623333
up	1.59	2.02	2.07	7.07	7.08	8.17	1.893333	7.44
down	18.24	20.13	18.91	7.86	6.84	9.22	19.09333	7.973333
up	0.38	0.33	0.36	1.89	2.02	2.09	0.356667	2
down	5.61	4.75	5.58	2.18	2.78	2.43	5.313333	2.463333
up	0.19	0.12	0.17	1.17	1.3	1.46	0.16	1.31
up	3.75	2.96	3.06	11.64	9.09	12.07	3.256667	10.93333
up	2.02	1.8	2.25	7.19	7.59	8.71	2.023333	7.83
up	4.58	3.61	5.73	15.44	12.67	14.67	4.64	14.26
up	5.37	6.26	6.02	16.86	22.21	12.57	5.883333	17.21333
up	1.66	1.7	2.13	5.81	6.27	6.13	1.83	6.07
up	1.85	2.13	1.92	5.81	6.29	5.97	1.966667	6.023333
up	0.73	0.69	0.61	2.54	2.18	2.03	0.676667	2.25
up	2.87	3.59	2.89	6.01	5.19	6.79	3.116667	5.996667
up	2.14	2.24	2.13	3.82	4.65	3.92	2.17	4.13
up	2.71	2.97	2.49	9.72	7.69	9.78	2.723333	9.063333
up	22.15	27.37	23.27	68.31	67.14	69.88	24.26333	68.44333
up	3.08	2.6	2.73	6.11	5.83	5.72	2.803333	5.886667
up	4.26	3.95	4.07	9.45	13.1	8.73	4.093333	10.42667
up	1.46	1.5	1.65	5.5	5.15	5.84	1.536667	5.496667
up	7.64	6.03	7.13	13.8	15.62	14.1	6.933333	14.50667
down	0.24	0.28	0.3	0	0	0	0.273333	0
up	0.01	0.01	0.01	0.25	0.38	0.25	0.01	0.293333
up	0.35	0.18	0.21	2.23	1.87	2.49	0.246667	2.196667
up	2.36	2.59	2.54	17	21	16.94	2.496667	18.31333
up	3.14	5.67	4.79	9.5	10.97	10.01	4.533333	10.16
up	4.59	5.04	5.37	8.43	10.92	9.46	5	9.603333
down	4.91	4.41	3.87	0.6	0.83	1.21	4.396667	0.88
up	0.63	0.65	0.44	4.98	5.36	5.28	0.573333	5.206667
up	0.15	0.17	0.53	2.63	3.62	2.16	0.283333	2.803333
up	0.78	0.78	0.33	5.65	3.35	4.22	0.63	4.406667
up	1.12	0.71	0.71	5.27	4.98	5.3	0.846667	5.183333
down	26.99	21.47	22.85	10.21	13.73	8.76	23.77	10.9
up	4.06	4.6	5.14	11.89	13.15	12.5	4.6	12.51333
up	0.59	0.35	0.73	2.44	2.86	2.8	0.556667	2.7
up	0	0	0	3.03	5.13	3.38	0	3.846667
up	0	0	0	0.31	0.3	0.18	0	0.263333
up	12.92	13.39	12.43	37.66	34.67	40.07	12.91333	37.46667
up	1.55	2.55	2.15	6.01	10.65	5.14	2.083333	7.266667
down	3.98	2.98	3.78	1.4	1.35	1.44	3.58	1.396667
up	5.3	5.83	6.47	15.14	20.16	13.66	5.866667	16.32
down	4.99	5.77	5.65	1.87	0.99	1.75	5.47	1.536667
up	1.42	1.84	1.7	4.74	4.2	4.96	1.653333	4.633333
up	0	0	0	1.42	0.64	1.41	0	1.156667
up	2.86	3.05	2.98	7.3	6.95	8.87	2.963333	7.706667
up	0.42	0.47	0.45	2.02	1.65	3.1	0.446667	2.256667
up	2.67	2.77	2.25	5.63	6.21	6.33	2.563333	6.056667
down	4.41	4.09	5.26	1.71	1.22	1.69	4.586667	1.54
up	3.83	2.57	3.05	10.84	10	9.71	3.15	10.18333

up	3.62	5.3	4.22	119.34	107.65	132.09	4.38	119.6933
up	37.45	30.54	35.02	65.35	78.67	56.11	34.33667	66.71
up	94.19	98.34	102.21	208.1	247.28	215.73	98.24667	223.7033
up	4.16	4.68	3.67	15.04	12.7	16.58	4.17	14.77333
up	1.45	1.18	1.61	3.03	3.53	4.03	1.413333	3.53
up	3.31	2.8	3.36	5.79	5.82	6.1	3.156667	5.903333
up	0.77	0.7	0.72	1.79	1.97	1.84	0.73	1.866667
up	2.65	3.18	2.52	7.67	8.17	8.27	2.783333	8.036667
up	1.87	1.49	1.96	5.67	5.19	5.62	1.773333	5.493333
up	0.86	0.87	0.84	1.82	1.88	1.8	0.856667	1.833333
up	3.07	2.55	2.97	14.26	12.72	14.26	2.863333	13.74667
up	0.48	0.75	0.22	3.9	3.08	4.14	0.483333	3.706667
up	0.15	0.21	0.25	0.89	0.83	0.99	0.203333	0.903333
up	2.2	2.3	2.3	4.63	4.07	5.84	2.266667	4.846667
up	2.46	2.18	2.03	7.14	7.7	7.75	2.223333	7.53
up	0	0	0	1.15	2.61	0.63	0	1.463333
up	5.35	4.61	5.12	9.07	9.93	10.05	5.026667	9.683333
up	0.95	0.89	1.11	1.66	2.39	2.1	0.983333	2.05
up	0.79	1	1.77	4.07	6.99	5.06	1.186667	5.373333
up	3.36	4.48	3.75	10.83	8.11	8.42	3.863333	9.12
up	0.07	0.09	0.1	0.3	0.33	0.38	0.086667	0.336667
up	0.11	0.1	0.14	2.16	1.51	2	0.116667	1.89
up	5.56	5.35	4.84	10.1	9.73	11.29	5.25	10.37333
up	6	7.27	6.49	14.16	14.49	14.31	6.586667	14.32
up	5.53	5.32	5.92	10.17	14.87	10.6	5.59	11.88
up	2.59	2.47	1.46	6.05	5.95	7.29	2.173333	6.43
up	0	0.01	0	38.65	49.2	40.75	0.003333	42.86667
up	1.32	2.01	1.71	4.36	3.3	4.47	1.68	4.043333
down	0.54	0.28	0.24	0	0	0	0.353333	0
up	3.94	3.55	4.22	13.18	14.91	12.23	3.903333	13.44
up	13.17	14.17	14.55	31.61	33.02	29.46	13.96333	31.36333
up	5.01	5.54	6.18	15.48	18.85	14.75	5.576667	16.36
up	1.86	2.01	1.97	10.3	8.57	10.98	1.946667	9.95
up	2.38	2.64	4.12	9.69	13.09	14.81	3.046667	12.53
up	0.17	0.31	0.27	0.99	2.56	3.7	0.25	2.416667
up	0.86	0.89	0.94	3.21	4.7	3.97	0.896667	3.96
down	10.13	8.65	9.15	3.56	4.77	4.14	9.31	4.156667
up	4.88	5.29	5.13	10.14	10.35	10.35	5.1	10.28
down	63.95	63.68	62.22	26.59	28.52	27.04	63.28333	27.38333
up	0	0	0.01	0.55	0.2	0.38	0.003333	0.376667
up	1.52	1.14	1.29	3.32	4.16	3.21	1.316667	3.563333
down	9.96	9.15	8.83	3.12	3.33	2.88	9.313333	3.11
down	39.11	34.3	38.29	8.17	8.87	9.64	37.23333	8.893333
down	0.35	0.48	0.86	0	0	0	0.563333	0
up	0.06	0.08	0.05	0.48	0.55	0.52	0.063333	0.516667
up	0.02	0.02	0.02	0.17	0.14	0.15	0.02	0.153333
up	3.02	2.85	3.46	5.88	8.03	6.6	3.11	6.836667
down	2.13	1.88	1.93	0.84	0.79	0.8	1.98	0.81
up	0.52	0.3	0.62	1.55	1.66	1.87	0.48	1.693333
up	3.5	4.32	5.4	10.24	11.36	7.57	4.406667	9.723333
up	0.1	0.14	0.11	0.37	0.4	0.41	0.116667	0.393333
up	3.13	3.17	3.23	7.77	8.76	7.06	3.176667	7.863333
up	1.01	0.95	1.09	3.49	3.38	3.27	1.016667	3.38
up	1.06	0.46	0.8	4.94	4.38	3.6	0.773333	4.306667
up	0.44	0.47	0.34	1.41	1.41	1.35	0.416667	1.39
up	0.05	0.03	0.11	0.88	0.76	1.14	0.063333	0.926667
up	1.51	2.17	1.86	4.35	5.13	3.64	1.846667	4.373333
up	17.43	20.99	20.64	47.71	53.36	51.17	19.68667	50.74667

up	0.22	0.13	0.36	2.6	2.3	2.77	0.236667	2.556667
up	1.43	1.23	1.25	2.49	2.41	2.9	1.303333	2.6
up	0	0	0	60.88	56.48	56.39	0	57.91667
up	0	0	0	52.91	27.8	73.16	0	51.29
up	0.02	0	0	44.69	69.03	40.52	0.006667	51.41333
up	1.11	0.85	0.89	2.13	2.33	1.91	0.95	2.123333
up	2.53	2.4	2.24	4.39	3.91	5.23	2.39	4.51
up	1.44	2.47	1.85	10.38	9.31	7.87	1.92	9.186667
up	6.22	7.11	6.56	19.61	15.44	20.28	6.63	18.44333
up	14.31	17.43	17.11	29.6	42.7	25.18	16.28333	32.49333
up	37.27	37.22	38.63	66.21	71.8	74.02	37.70667	70.67667
up	2.82	3.56	3.11	9.13	7.76	10.48	3.163333	9.123333
up	1.72	1.64	1.25	7.7	7.26	6.72	1.536667	7.226667
up	0.6	0.3	0.61	2.29	2.16	2.28	0.503333	2.243333
up	1.87	1.94	2.61	6.78	12.44	7.98	2.14	9.066667
up	0.06	0.04	0.1	0.72	0.67	0.67	0.066667	0.686667
down	1.81	1.68	1.58	0.58	0.69	0.67	1.69	0.646667
up	6.76	7.97	6.59	31.23	36.67	30.41	7.106667	32.77
down	0.13	0.05	0.13	0	0	0	0.103333	0
up	54.53	53.13	54.17	96	117.32	92.68	53.94333	102
up	16.23	20.87	13.77	64.45	89.26	54.27	16.95667	69.32667
down	5.42	4.8	4.64	2.11	2.09	2.11	4.953333	2.103333
up	0.52	0.36	0.49	1.65	1.75	1.5	0.456667	1.633333
up	3.35	4.86	4.4	11.35	9.85	10.34	4.203333	10.51333
up	0.43	0.65	0.49	3.59	3.37	3.59	0.523333	3.516667
up	42.18	50.63	54.48	87	127.17	85.41	49.09667	99.86
up	24.93	35.59	23.66	57.27	53.47	53.39	28.06	54.71
up	0.59	1.12	1.19	3.38	4.56	4.09	0.966667	4.01
up	0.96	1.13	1.3	13.21	12.32	13.13	1.13	12.88667
down	5.95	6.59	5.39	2.52	2.9	2.87	5.976667	2.763333
down	0.31	0.6	0.64	0	0	0	0.516667	0
down	3.01	3.06	3.26	0.99	1.16	1.04	3.11	1.063333
up	2.77	3.48	3.23	13.04	9.02	13.79	3.16	11.95
down	0.35	0.24	0.36	0.02	0.03	0.03	0.316667	0.026667
down	7.34	6.24	6.52	2.37	1.96	2.61	6.7	2.313333
up	14.78	16.42	14.27	33.76	33.63	35.24	15.15667	34.21
up	0.82	0.67	0.66	2.07	1.82	1.94	0.716667	1.943333
up	0	0	0	3.27	1.88	0.7	0	1.95
up	0.17	0.19	0.18	1.25	0.95	1.21	0.18	1.136667
up	5.09	4.94	4.97	8.83	10.58	8.72	5	9.376667
up	0.11	0.1	0.06	0.62	0.51	0.45	0.09	0.526667
up	3.11	2.68	3.28	6.32	7.28	6.19	3.023333	6.596667
up	1.22	0.78	1.04	3.97	4.84	4.18	1.013333	4.33
up	0	0	0.04	1.23	1.31	0.79	0.013333	1.11
up	0.12	0.13	0.12	0.79	0.93	0.7	0.123333	0.806667
up	0.37	0.5	0.45	1.52	1.83	1.79	0.44	1.713333
down	0.62	0.73	0.68	0.24	0.31	0.26	0.676667	0.27
down	32.53	26.03	32.63	12.06	13.95	10.84	30.39667	12.28333
up	1.2	1.66	1.83	3.58	5.1	5.39	1.563333	4.69
up	0.08	0.03	0.01	16.2	21.28	15.28	0.04	17.58667
down	0.44	0.62	0.51	0.02	0.03	0.01	0.523333	0.02
up	0.71	0.7	0.78	2.11	2.22	2.23	0.73	2.186667
up	0.07	0.25	0.18	1.18	1.05	1.25	0.166667	1.16
up	4.04	3.43	3.77	6.77	7.76	7.62	3.746667	7.383333
down	1.08	1.11	1.45	0.27	0.25	0.24	1.213333	0.253333
up	0	0	0	0.74	0.35	0.3	0	0.463333
up	0.67	0.6	0.64	11.83	12.71	13.65	0.636667	12.73
down	0.77	0.52	0.22	0	0	0	0.503333	0

up	0	0	0	0.11	0.17	0.29	0	0.19
up	0.66	0.62	0.62	1.35	1.5	1.05	0.633333	1.3
up	1.33	2.97	2.5	5.75	8.64	6.13	2.266667	6.84
up	0.99	0.97	0.93	2.85	2.41	2.2	0.963333	2.486667
up	11.83	11.41	11.85	32.16	35.64	30.99	11.69667	32.93
up	6.04	8.28	7.11	21.88	27.71	16.39	7.143333	21.99333
up	4.59	5.84	6.22	12.99	9.73	16.49	5.55	13.07
up	0.97	0.97	0.9	7.9	9.02	8.67	0.946667	8.53
down	8.81	7.9	8.83	3.02	4.62	3.23	8.513333	3.623333
down	26.48	23.88	24.53	6.85	6.89	8.69	24.96333	7.476667
up	1.48	1.14	1.28	4.13	4.46	4.71	1.3	4.433333
up	0.36	0.38	0.36	0.81	0.88	0.75	0.366667	0.813333
up	0.48	0.66	0.48	1.54	1.46	1.19	0.54	1.396667
up	3	3.54	4.01	13.53	26.14	21.75	3.516667	20.47333
up	4.4	5.3	2.84	52.48	26.68	48.5	4.18	42.55333
up	0.67	1.3	0.92	12.94	11.87	13.6	0.963333	12.80333
up	3.36	3.08	3.4	36.13	25.44	32.71	3.28	31.42667
up	5.2	4.45	6.25	20.29	25.11	20.02	5.3	21.80667
up	2.2	1.74	1.79	3.78	4.9	3.64	1.91	4.106667
up	4.1	3.99	3.36	8.12	8.64	8.13	3.816667	8.296667
up	1.8	2.38	3.3	5.94	5.57	6.69	2.493333	6.066667
up	9.9	10.5	10.66	19.62	27.79	20.89	10.35333	22.76667
up	9	8.62	8.2	29.31	23.55	29.24	8.606667	27.36667
down	7.93	8.5	7.93	2.81	2.95	3.45	8.12	3.07
up	1.99	2.28	2.3	6.38	6.58	6.89	2.19	6.616667
up	3.91	2.86	3.7	9.47	9.64	7.87	3.49	8.993333
up	1.5	1	0.73	5.25	4.21	8.12	1.076667	5.86
down	7.36	5.59	6.85	2.32	1.97	2.92	6.6	2.403333
down	9.95	16.03	11.51	3.28	2.13	2.61	12.49667	2.673333
up	8.36	7.44	7.94	22.88	28.17	23.22	7.913333	24.75667
down	5.39	5.55	5.44	2.07	1.78	1.64	5.46	1.83
down	8.73	7.79	9.73	3.52	4.08	3.69	8.75	3.763333
down	1.97	1.98	1.97	0.48	0.53	0.57	1.973333	0.526667
up	1.22	1.16	1.36	2.62	3.23	2.66	1.246667	2.836667
up	1.54	2.15	2.07	14.85	15.91	14.3	1.92	15.02
down	2.18	2.78	2.86	1.21	1.16	1.06	2.606667	1.143333
up	0.51	0.45	0.46	1.47	1.14	1.49	0.473333	1.366667
up	0.63	0.91	0.65	1.41	1.77	1.38	0.73	1.52
up	0.99	0.76	0.98	1.89	2.28	1.72	0.91	1.963333
down	2.13	1.98	1.79	0.8	0.79	0.78	1.966667	0.79
down	2.31	2.5	2.38	0.99	1.15	1.08	2.396667	1.073333
down	0.95	1.02	1.47	0.16	0.24	0.29	1.146667	0.23
up	6.29	6.27	5.72	12.34	12.03	15.5	6.093333	13.29
down	8.68	7.16	7.56	2.57	3.09	2.96	7.8	2.873333
up	11.5	12.89	9.8	22.66	23.19	24.88	11.39667	23.57667
up	13.01	14.49	16.45	30.03	22.96	29.02	14.65	27.33667
up	0	0	0	65.27	81.51	61.04	0	69.27333
up	3.02	3.6	2.73	30.27	28.33	33.27	3.116667	30.62333
up	1.6	1.49	1.81	3.13	2.89	3.97	1.633333	3.33
up	0.29	0.23	0.19	0.62	0.84	0.64	0.236667	0.7
up	10.41	9.81	11.55	24.97	19.29	22.82	10.59	22.36
up	6.31	7.46	5.55	13.4	12.39	19.25	6.44	15.01333
up	0.14	0.11	0.11	0.82	0.78	0.9	0.12	0.833333
up	0.51	0.74	0.64	1.45	1.35	1.8	0.63	1.533333
down	5.13	5.22	5.76	2.09	2.98	2.26	5.37	2.443333
up	18.13	16.14	17.16	31.17	32.85	35.34	17.14333	33.12
up	11.73	7.69	9.26	20.86	17.67	27.12	9.56	21.88333
up	4.51	4.77	4.03	8.33	7.92	9.05	4.436667	8.433333

up	18.32	23.47	19.07	35.21	35.52	41.48	20.28667	37.40333
down	1.49	1.62	1.75	0.45	0.4	0.59	1.62	0.48
down	0.87	0.86	0.86	0.13	0.17	0.12	0.863333	0.14
up	6.79	6.21	6.54	17.28	22.49	17.5	6.513333	19.09
up	2.02	2.05	2.21	5.33	5.43	5.01	2.093333	5.256667
up	9.08	9.73	9.71	25.27	19.05	25.41	9.506667	23.24333
up	1.24	0.98	0.97	4.4	5.24	4.39	1.063333	4.676667
up	2.03	2.2	1.86	7.27	7.78	7.21	2.03	7.42
up	1.26	1.49	1.25	3.67	3.29	3.46	1.333333	3.473333
up	0.01	0.11	0.01	4.19	5.52	2.85	0.043333	4.186667
up	0.15	0.16	0.11	25.67	21.12	25.57	0.14	24.12
up	0.55	0.35	0.74	1.47	2.18	1.6	0.546667	1.75
up	0.77	0.99	0.64	1.65	2.07	1.52	0.8	1.746667
up	14.12	12.16	13.48	23.95	33.31	30.52	13.25333	29.26
up	10.35	9.17	10.06	38.13	43.36	38.12	9.86	39.87
up	70.15	73.35	72.46	193	238.28	187.67	71.98667	206.3167
up	9.31	10.04	10.2	20.31	21.57	19.73	9.85	20.53667
up	8.96	7.67	8.12	16.41	14.97	18.09	8.25	16.49
up	3.45	3.87	4.02	10.07	10.27	10.67	3.78	10.33667
up	1.8	1.96	2.03	4.89	5.6	5.36	1.93	5.283333
down	2.25	2.24	2.25	1.02	0.9	0.82	2.246667	0.913333
up	1.02	1.41	1.16	5.66	3.75	6.75	1.196667	5.386667
up	4.34	3.92	4.06	16.88	18.69	17.47	4.106667	17.68
up	0.07	0.1	0.08	0.23	0.3	0.28	0.083333	0.27
up	0.18	0.15	0.17	0.63	0.55	0.69	0.166667	0.623333
up	0.6	0.6	0.62	7.52	7.4	7.66	0.606667	7.526667
up	0.05	0.07	0.07	0.27	0.25	0.23	0.063333	0.25
up	1.7	1.74	1.45	2.77	2.96	3.82	1.63	3.183333
up	1.78	2.25	2.42	6.94	6.94	7.22	2.15	7.033333
up	8.22	10.63	10.39	27.25	25.3	28.7	9.746667	27.08333
up	42.88	40.28	44.39	112.46	131.93	106.73	42.51667	117.04
up	17.94	21.1	19.36	47.1	47.01	47.03	19.46667	47.04667
up	1.54	2.37	1.9	4.75	4.3	4	1.936667	4.35
up	6.2	5.39	6.23	23.11	28.16	24.16	5.94	25.14333
up	4.15	4.28	3.34	7.58	7.77	7.56	3.923333	7.636667
up	0.03	0	0.01	0.68	0.48	0.57	0.013333	0.576667
up	5.72	5.1	6.31	14.29	17.57	14.27	5.71	15.37667
up	2.23	3.33	2.25	5.22	5.72	6.31	2.603333	5.75
up	4.92	6.05	5.81	13.21	10.46	11.64	5.593333	11.77
up	0.49	1.08	0.75	5.21	4.38	3.6	0.773333	4.396667
up	0.98	1.08	1.1	5.15	5.6	4.26	1.053333	5.003333
up	0	0	0	0.65	0.47	0.94	0	0.686667
down	2.95	1.04	1.62	0	0	0	1.87	0
up	3.04	2.84	2.56	7.05	6.77	7.38	2.813333	7.066667
up	0.11	0.12	0.08	0.44	0.43	0.52	0.103333	0.463333
up	0.3	0.08	0.22	3.97	4.47	3.81	0.2	4.083333
up	8.36	7.71	7.84	42.9	45.37	42.99	7.97	43.75333
up	0.55	0.93	0.86	3.19	2.79	3.57	0.78	3.183333
up	0	0	0	0.77	0.68	0.77	0	0.74
up	0	0	0	0.58	0.34	0.66	0	0.526667
down	1.42	1.18	1.45	0.34	0.38	0.37	1.35	0.363333
up	2.48	2.7	2.63	8.36	8.2	7.63	2.603333	8.063333
up	4.79	5.98	4.81	9.29	13.85	9.5	5.193333	10.88
down	4.37	4.41	4.05	1.77	1.23	1.89	4.276667	1.63
up	1.49	1.35	1.55	14.19	22.34	13.35	1.463333	16.62667
up	0.99	0.5	0.38	9.13	8.88	10.31	0.623333	9.44
up	6.36	7.27	7.05	45.8	43.45	46.46	6.893333	45.23667
up	0.61	0.7	0.87	131.68	137.38	130.42	0.726667	133.16

up	0.65	0.62	0.63	5.31	5.13	4.81	0.633333	5.083333
up	0.72	0.57	0.22	3.14	3.94	3.5	0.503333	3.526667
down	1.17	0.93	1.07	0.15	0.17	0.07	1.056667	0.13
up	9.96	11.36	11.43	25.7	33.2	25.48	10.91667	28.12667
up	10.4	11	10.25	30.41	26.14	32.23	10.55	29.59333
up	0.5	0.49	0.29	2.98	4.54	2.53	0.426667	3.35
down	2.12	2.41	2.24	0.64	0.91	0.48	2.256667	0.676667
down	7.09	6.41	6.87	2.18	2.49	2.39	6.79	2.353333
up	2.03	2.53	1.82	4.97	4.6	5.17	2.126667	4.913333
up	9.66	10.31	9.92	24.34	24.64	24.71	9.963333	24.56333
up	0.83	0.49	0.57	1.41	2.26	2.76	0.63	2.143333
down	9.14	7.03	9.26	2.28	2.76	1.93	8.476667	2.323333
down	3.65	3.93	2.9	0.98	0.8	1.24	3.493333	1.006667
up	0	0	0	0.92	4.59	1.29	0	2.266667
up	3.07	2.7	3.05	6.68	7.54	7.03	2.94	7.083333
up	35.68	35.16	34.63	73.59	67.76	83.44	35.15667	74.93
up	8.25	8.53	15.41	44.45	48.77	32.79	10.73	42.00333
down	2.64	2.2	2.58	0.9	0.98	0.96	2.473333	0.946667
up	1.37	1.35	1.05	4.51	5.06	4.31	1.256667	4.626667
up	16.22	16.62	15.27	30.46	31.63	31.55	16.03667	31.21333
down	1.58	1.02	1.37	0.26	0.33	0.23	1.323333	0.273333
up	0.04	0.06	0.02	0.88	1.21	1.3	0.04	1.13
up	0.1	0	0.06	1.64	3.12	1.8	0.053333	2.186667
up	0.28	0.12	0.3	1.32	2.25	1.96	0.233333	1.843333
up	0.51	1.35	1.12	6.08	8.53	6.71	0.993333	7.106667
up	5.24	5.82	5.94	12.31	11.23	11.8	5.666667	11.78
down	51.29	46.69	35.43	18.75	18.75	19.88	44.47	19.12667
up	1.69	2.21	2.06	5.48	6.38	5.59	1.986667	5.816667
up	0.19	0.24	0.33	0.7	0.9	0.74	0.253333	0.78
down	7.79	7.56	7.24	2.94	3.15	3.5	7.53	3.196667
up	1.56	1.31	1.5	4.05	4.75	6.05	1.456667	4.95
up	13.23	14.47	13.41	30.9	27.86	33.02	13.70333	30.59333
up	2.77	3.21	2.71	7.27	7.92	7.44	2.896667	7.543333
down	7.26	6.59	6.6	1.13	1.1	1.3	6.816667	1.176667
up	6.65	7.59	7.7	20.14	19.36	21.76	7.313333	20.42
down	19.66	19.62	19.14	7.62	8.42	8.27	19.47333	8.103333
up	10.65	12.9	11.67	23.85	26.14	22.59	11.74	24.19333
up	14.3	17.03	19.66	81.4	103.52	91.37	16.99667	92.09667
up	42.61	67.15	58.02	280.89	287.16	274.81	55.92667	280.9533
up	3.07	2.64	2.47	6.23	8.41	8.14	2.726667	7.593333
up	82.54	99.75	84.88	504.22	430.04	500.68	89.05667	478.3133
up	3.08	3.3	3.01	8.24	9.21	8.25	3.13	8.566667
up	1.43	2.1	1.27	5.74	5.55	6.49	1.6	5.926667
up	0.17	0.15	0.16	0.83	1.4	0.94	0.16	1.056667
up	13.3	14.46	13.63	28.82	28.84	30.21	13.79667	29.29
up	0	0	0	0.45	0.3	0.17	0	0.306667
up	12.62	16.64	16.91	30.56	35.77	31.35	15.39	32.56
up	0.01	0.01	0.01	0.18	0.26	0.19	0.01	0.21
up	0	0	0	2.31	2.42	2.16	0	2.296667
up	5	5.11	4.76	16.06	10.21	15.97	4.956667	14.08
up	1.54	1.5	1.26	3.3	2.68	3.19	1.433333	3.056667
up	0.5	0.63	0.5	2.1	1.56	2.41	0.543333	2.023333
up	7.13	8.45	6.62	23.73	31.3	22.13	7.4	25.72
up	7.24	7.45	8.17	22.65	20.51	25.11	7.62	22.75667
up	0.88	0.37	0.38	2.8	3.12	3.11	0.543333	3.01
up	2.61	2.62	2.7	4.98	5.43	5.41	2.643333	5.273333
up	3.17	3.68	3.45	6.68	7.83	5.83	3.433333	6.78
up	5.47	5.92	6.35	11.18	11.66	10.55	5.913333	11.13

up	2.5	2.47	2.2	5.21	5.12	6.08	2.39	5.47
up	1.17	1.8	1.77	5.59	5.38	4.86	1.58	5.276667
up	1.37	1.01	1.13	3.68	3.77	4.29	1.17	3.913333
up	0.96	2.18	0.59	12.18	11.25	15.29	1.243333	12.90667
up	0.04	0	0.02	0.99	1.16	1.55	0.02	1.233333
up	0.69	0.72	0.75	5.89	5.83	6.15	0.72	5.956667
up	6.41	6.15	5.77	10.59	13.57	10.24	6.11	11.46667
up	9.91	13.04	9.45	33.8	23.51	37.44	10.8	31.58333
up	2.25	1.8	2.85	5	5.74	6.25	2.3	5.663333
up	7.85	9.94	9.11	19.66	20.22	18.23	8.966667	19.37
up	25.74	26.1	26.43	84.55	86.44	88.03	26.09	86.34
up	2.08	1.81	1.78	4.64	5.58	5.31	1.89	5.176667
up	0	0	0	0.61	0.13	0.19	0	0.31
up	0	0	0	1.51	1.97	1.13	0	1.536667
up	1.02	0.72	0.89	17.28	18.21	18.64	0.876667	18.04333
up	2.51	2.63	2.58	44.45	36.88	45.5	2.573333	42.27667
up	0.25	0.17	0.25	0.98	0.74	0.85	0.223333	0.856667
up	0.11	0.1	0.15	0.4	0.52	0.41	0.12	0.443333
up	0.43	0.48	0.3	1.32	2.17	1.53	0.403333	1.673333
up	2.79	3.06	2.34	9.11	6.61	10.07	2.73	8.596667
up	6.38	6.42	5.39	19.79	19.81	20.16	6.063333	19.92
up	9.06	8.84	9.7	19.74	24.77	19.82	9.2	21.44333
up	0	0	0	0.17	0.52	0.46	0	0.383333
up	6.9	6.59	7.74	15.35	14.95	17.09	7.076667	15.79667
up	1.94	2.46	1.28	5.31	9.48	5.45	1.893333	6.746667
up	10.64	9.34	9.14	19.24	22.42	21.21	9.706667	20.95667
up	3.22	3.99	3.94	8.64	8.09	8.73	3.716667	8.486667
up	0.48	0.55	0.41	1.5	1.47	1.44	0.48	1.47
up	0.86	0.85	0.77	3.23	2.98	3.05	0.826667	3.086667
up	2.09	1.75	2.72	6.51	5.58	8.77	2.186667	6.953333
down	5.23	4.3	4.78	1.53	1.76	1.43	4.77	1.573333
down	0.24	0.29	0.51	0	0	0	0.346667	0
down	0.31	0.97	1.66	0	0	0	0.98	0
up	0.95	1.59	1.59	3.56	4.41	4.46	1.376667	4.143333
up	0.09	0.06	0.1	1.03	1.13	1.05	0.083333	1.07
down	6.86	6.42	6.22	1.9	1.8	2.14	6.5	1.946667
down	237.05	199.09	247.15	88.02	122.43	95.64	227.7633	102.03
up	0	0	0	0.19	0.21	0.16	0	0.186667
up	0	0	0	1.82	1.34	1.47	0	1.543333
up	0	0	0	0.88	1.07	1.17	0	1.04
up	0	0	0	1.92	1.84	1.68	0	1.813333
up	0.04	0.09	0.08	2.4	3.62	2.54	0.07	2.853333
up	0	0	0.68	764.04	829.99	733.86	0.226667	775.9633
up	0	0.05	0	3.44	1.67	3.68	0.016667	2.93
down	24.97	27.25	26.82	11.03	11.41	11.44	26.34667	11.29333
up	0.02	0.02	0.03	0.15	0.19	0.19	0.023333	0.176667
down	12.74	12.87	13.91	5.01	5.94	6.59	13.17333	5.846667
down	12.39	11.31	11.64	3.44	3.85	3.88	11.78	3.723333
up	0.21	0.4	0.58	1.79	2.33	2.82	0.396667	2.313333
up	4.61	4.97	4.91	11.17	10.87	11.07	4.83	11.03667
up	2.72	3.46	3.09	7.05	6.44	5.77	3.09	6.42
up	509.16	543.55	513.28	1050.2	1113.55	995.47	521.9967	1053.073
up	0.11	0.19	0.07	1.29	0.89	0.64	0.123333	0.94
up	0.27	0.08	0.15	1.23	1.3	1.65	0.166667	1.393333
up	2.01	1.96	1.49	13.61	17.56	13.99	1.82	15.05333
up	0	0	0	8	7.24	8.31	0	7.85
down	5.03	4.87	5.37	2.17	2.2	2.44	5.09	2.27
up	0.47	0.24	0.35	1.39	1.3	1.43	0.353333	1.373333

up	24.26	21.22	22.05	52.32	44.67	48.39	22.51	48.46
up	9.92	9.95	9.24	20.68	20.55	19.11	9.703333	20.11333
up	2.96	3.37	2.99	5.94	7.23	4.76	3.106667	5.976667
up	4.86	4.59	4.65	11.02	8.71	11.09	4.7	10.27333
down	7.22	6.73	5.67	3.25	3.08	2.66	6.54	2.996667
down	4.13	3.69	3.7	1.18	1.57	1.51	3.84	1.42
down	2.37	2.19	2.2	0.96	1.07	1.01	2.253333	1.013333
down	114.34	103	127.75	46.97	52.62	58.96	115.03	52.85
up	0	0	0	0.36	0.63	0.27	0	0.42
up	0.43	0.49	0.48	4.13	3.76	4.21	0.466667	4.033333
up	0.89	0.41	0.5	4.52	4.7	4.09	0.6	4.436667
up	2.95	2.24	2.38	14.11	10.9	14.96	2.523333	13.32333
up	3.69	4.77	4.82	32.12	29.73	33.64	4.426667	31.83
up	5.84	4.76	4.65	12.59	17.02	13.55	5.083333	14.38667
down	0.73	1.08	0.94	0.22	0.21	0.27	0.916667	0.233333
up	0.6	0.82	0.38	9.62	4.79	10.55	0.6	8.32
up	0	0	0	2.24	0.4	2.41	0	1.683333
up	0	0	0	1.03	1.02	1.26	0	1.103333
up	2.25	3.1	1.47	8.23	5.86	10.39	2.273333	8.16
up	1.83	2.22	1.45	5.47	5	7.43	1.833333	5.966667
up	3.92	3.9	6.07	17.17	17.85	14.62	4.63	16.54667
down	3.57	2.89	3.26	1.31	1.15	1.48	3.24	1.313333
up	8.72	11.68	11.45	50.91	36.32	50.31	10.61667	45.84667
up	16.01	15.22	16.24	30.19	37.3	30.91	15.82333	32.8
up	11.14	11.94	12.44	22.33	29.64	21.09	11.84	24.35333
up	2.28	1.89	1.81	28.2	37.35	28.87	1.993333	31.47333
up	2	2.3	2.32	32.23	28.1	32.11	2.206667	30.81333
up	0.2	0.2	0.12	1.51	1.87	1.91	0.173333	1.763333
up	1.29	1.88	2.27	7.69	4.97	6.69	1.813333	6.45
up	5.11	4.81	6.48	12.11	16.62	12.01	5.466667	13.58
down	5.14	2.78	3.93	1.26	1.34	1.06	3.95	1.22
up	8.16	6.13	6.72	12.82	15.84	13.51	7.003333	14.05667
up	6.32	6.12	6.83	11.73	14.67	11.18	6.423333	12.52667
up	0	0	0	0.66	0.72	0.75	0	0.71
up	4.1	4.14	4.21	13	13.5	13.4	4.15	13.3
up	0.64	0.13	0.23	3.36	4.36	3.13	0.333333	3.616667
up	0.33	0.02	0.35	4.29	5.52	4.21	0.233333	4.673333
down	1.54	1.54	1.42	0.4	0.37	0.63	1.5	0.466667
up	53.55	54.37	53.03	137.6	137.62	141.18	53.65	138.8
up	0.58	0.78	0.56	3.38	3.59	3.23	0.64	3.4
up	10.88	17.33	10.96	35.48	25.74	34.22	13.05667	31.81333
down	4.26	4.36	3.46	1.47	1.22	1.41	4.026667	1.366667
up	0	0	0	1.16	1.47	1.07	0	1.233333
up	34.82	37.75	37.06	82.7	110.17	82.42	36.54333	91.76333
up	0.22	0.3	0.48	1.78	2.31	1.58	0.333333	1.89
down	5.23	5.63	5.65	1.66	1.82	1.61	5.503333	1.696667
up	1.54	1.8	1.29	9.64	7.09	9.81	1.543333	8.846667
up	0.25	0.23	0.41	1.58	2.16	1.26	0.296667	1.666667
up	5.74	6.94	7.23	11.95	11.63	14.41	6.636667	12.66333
down	3.34	3.39	3.2	0.72	0.62	0.84	3.31	0.726667
up	0.17	0.22	0.21	1.42	1.43	1.45	0.2	1.433333
down	108.44	100.22	102.09	37.86	52.23	39.02	103.5833	43.03667
up	3.18	3.13	3.36	8.26	9.32	7.74	3.223333	8.44
up	9.71	12.77	9	41.15	37.3	40.04	10.49333	39.49667
up	1.11	0.87	1.28	3.4	4.85	3.77	1.086667	4.006667
up	0.2	0.13	0.19	0.59	0.82	0.83	0.173333	0.746667
up	0.57	0.58	0.8	3.07	3.8	3.28	0.65	3.383333
up	2.47	2.32	2.25	5.58	4.77	4.92	2.346667	5.09

down	0.2	0.21	0.29	0	0	0	0.233333	0
up	1.62	1.86	1.48	7.54	5.94	8.16	1.653333	7.213333
up	1.57	1.84	1.6	6.25	6.07	6.89	1.67	6.403333
up	3.41	3.9	3.7	9.99	10.7	9.4	3.67	10.03
up	2.96	3.46	3.03	8.43	7.24	8.95	3.15	8.206667
up	1.28	1.13	1.45	3.37	3.9	3.61	1.286667	3.626667
up	0.03	0.02	0.04	2.75	3.05	2.63	0.03	2.81
up	0.01	0.03	0.12	2.58	3.52	1.95	0.053333	2.683333
up	2.7	2.49	2.8	5.11	5.52	4.9	2.663333	5.176667
down	0.34	0.5	0.42	0.15	0.11	0.13	0.42	0.13
down	15.76	16.41	16.9	4.41	6.55	4.03	16.35667	4.996667
up	3.53	4.87	4.42	14.52	14.43	14.23	4.273333	14.39333
up	2.25	2.08	2.05	5.27	5.8	5.68	2.126667	5.583333
up	2.42	1.46	1.87	8.32	9.62	7.5	1.916667	8.48
up	25.04	27.11	24.22	56.59	63.54	58.27	25.45667	59.46667
up	20.85	24	25.8	53.01	60.96	47.3	23.55	53.75667
up	0.09	0.31	0.22	5.52	6.47	5.34	0.206667	5.776667
up	44.31	44.95	48.64	99.35	88.3	113.57	45.96667	100.4067
up	1.8	2.31	1.37	5.31	9.41	6.99	1.826667	7.236667
up	0.09	0.14	0.11	0.5	0.74	0.66	0.113333	0.633333
up	0.05	0.14	0.15	1.32	1.42	1.37	0.113333	1.37
up	0.16	0.16	0.12	0.42	0.55	0.52	0.146667	0.496667
up	2.69	2.75	2.23	4.7	4.98	5.15	2.556667	4.943333
up	0.02	0.05	0.05	0.35	0.22	0.35	0.04	0.306667
up	0	0	0	0.24	0.19	0.34	0	0.256667
up	0	0	0	0.24	0.32	0.42	0	0.326667
up	0.1	0.13	0.15	0.77	0.9	0.63	0.126667	0.766667
up	3.41	3.96	3.31	9.88	8.95	9.56	3.56	9.463333
up	1.48	1.81	1.69	6.11	6.35	5.97	1.66	6.143333
up	3.93	4.77	4.6	14.37	17.76	14.14	4.433333	15.42333
up	2.95	3.58	2.95	11.49	13.35	10.34	3.16	11.72667
up	1.95	1.7	1.96	11.3	6.09	12.29	1.87	9.893333
up	0.91	1.08	0.46	10.87	11.61	12.68	0.816667	11.72
up	0.09	0.06	0.07	1.8	2.24	1.67	0.073333	1.903333
up	0.09	0.31	0.32	2.87	3.71	5.44	0.24	4.006667
up	1.61	1.54	2.05	50.2	54.83	46.41	1.733333	50.48
up	0.45	0.37	0.49	3.01	2.72	3.06	0.436667	2.93
down	0.2	0.15	0.2	0.03	0.05	0.02	0.183333	0.033333
down	4.89	5.23	5.8	2.22	2.8	2.3	5.306667	2.44
down	11.32	13.84	14.55	4.94	5.69	6.38	13.23667	5.67
up	0.93	1.1	1.02	2.23	2.49	2.46	1.016667	2.393333
down	12.29	13.63	11.17	2.67	2.01	2.58	12.36333	2.42
down	10.4	9.94	11.72	2.01	3.16	2.02	10.68667	2.396667
up	0.84	0.82	0.98	1.87	2.36	1.73	0.88	1.986667
up	1.61	1.14	1.33	2.94	2.87	2.57	1.36	2.793333
up	7.94	7.66	8.7	30.56	37.61	31.74	8.1	33.30333
up	1.4	1.55	0.78	6.64	3.17	7.61	1.243333	5.806667
down	4.42	5.12	5.02	1.33	1.71	0.99	4.853333	1.343333
down	2.49	3.05	1.98	1.05	0.93	1.07	2.506667	1.016667
up	0.04	0.03	0.04	0.42	0.39	0.47	0.036667	0.426667
up	1.93	1.62	2.36	8.71	7.64	7.07	1.97	7.806667
up	3.64	5.47	3.68	8.9	11.33	11.38	4.263333	10.53667
down	0.61	0.62	0.59	0.12	0.1	0.11	0.606667	0.11
down	7.32	7.79	7.97	0.78	0.99	0.78	7.693333	0.85
up	0.58	0.74	0.85	5.78	5.25	5.79	0.723333	5.606667
up	2.1	2.06	1.96	3.86	3.68	5.16	2.04	4.233333
down	0.72	1.6	0.97	0.01	0	0	1.096667	0.003333
up	0	0	0	0.39	0.33	0.29	0	0.336667

down	0.08	0.07	0.05	0	0	0	0.066667	0
down	23.48	23.08	22.38	8.96	8.59	7.01	22.98	8.186667
down	77.56	70.22	72.96	30.4	34.19	32.11	73.58	32.23333
up	0	0.05	0	3.45	3.5	2.61	0.016667	3.186667
up	14.71	13.52	15.8	27.8	33.05	29.82	14.67667	30.22333
up	1.5	1.66	1.42	4.66	4.67	4.35	1.526667	4.56
up	1.53	1.64	1.8	5.86	4.39	5.08	1.656667	5.11
down	6.28	6.31	7.16	2.82	2.24	3.34	6.583333	2.8
up	2.53	2.16	2.52	4.83	5.37	4.75	2.403333	4.983333
up	5.1	3.84	4.76	8.45	12.1	10.69	4.566667	10.41333
up	0	0	0	0.21	0.32	0.35	0	0.293333
down	73.11	61.57	72.94	25.77	36.7	24.61	69.20667	29.02667
up	6.79	8.04	6.49	13.8	11.43	14.73	7.106667	13.32
down	1.7	1.38	1.9	0.21	0.43	0.25	1.66	0.296667
up	0	0	0	0.46	0.2	0.16	0	0.273333
up	0	0	0	0.84	1.26	1.25	0	1.116667
up	0.63	0.92	0.68	1.92	2.01	2.47	0.743333	2.133333
up	25.75	28.11	26.96	66.98	62.93	68.03	26.94	65.98
up	0.94	1.11	0.91	2.56	2.35	2.62	0.986667	2.51
up	0.8	0.43	0.55	2.48	2.42	2.49	0.593333	2.463333
up	0.49	1.1	0.81	5.77	4.14	6.28	0.8	5.396667
down	8.13	7.57	7.9	3.47	4.1	3.42	7.866667	3.663333
up	50.96	49.44	51.06	145.13	140.05	141.8	50.48667	142.3267
down	7.26	6.4	5.45	2.84	2.68	2.77	6.37	2.763333
down	14.35	11.5	14.25	5.04	6.34	5.38	13.36667	5.586667
up	0.9	1.11	0.95	2.59	1.96	2.49	0.986667	2.346667
down	3.93	4.44	4.12	1.64	1.96	1.8	4.163333	1.8
up	45.91	72.58	52.32	258.26	139.63	268.73	56.93667	222.2067
up	1.81	1.72	1.85	5.51	6.72	5.96	1.793333	6.063333
up	2.73	3.32	2.43	7.09	6.15	7.87	2.826667	7.036667
up	3.62	3.36	3.59	6.78	7.69	6.8	3.523333	7.09
down	2.88	1.91	2.43	0.75	0.91	1.05	2.406667	0.903333
up	0	0	0	0.47	0.17	0.15	0	0.263333
up	9.94	11.52	10.73	20.36	21.63	20.9	10.73	20.96333
up	0.03	0.03	0.01	0.73	0.77	1.12	0.023333	0.873333
up	9.06	7.52	8.08	16.54	14.77	15.41	8.22	15.57333
up	0.36	0.72	1.17	4.1	4.56	4.26	0.75	4.306667
up	1.41	0.96	0.75	7.16	6.77	7.47	1.04	7.133333
up	0.78	0.92	0.74	2.43	2.46	2.69	0.813333	2.526667
down	2.37	2.11	2.35	0.73	0.73	0.87	2.276667	0.776667
up	18.42	27.06	21.56	44.64	46.04	45.13	22.34667	45.27
up	7.22	8.29	6.82	18.44	19.27	17.06	7.443333	18.25667
up	1.72	2.57	2.86	5.85	6.96	6.15	2.383333	6.32
up	0.88	0.88	0.82	2.31	2.32	2.48	0.86	2.37
up	7.85	6.88	7.99	23.42	23.22	24.67	7.573333	23.77
up	1.18	1.19	1.05	3.63	6.72	3.38	1.14	4.576667
up	1.22	1.05	1.19	3.25	3.34	4.03	1.153333	3.54
down	5.77	6.01	6.02	0.96	1.42	1.6	5.933333	1.326667
down	2.72	4.01	3.23	0.68	0.49	0.79	3.32	0.653333
up	0.39	0.42	0.38	1.07	1.25	1.2	0.396667	1.173333
up	2	1.73	1.89	3.63	3.54	4.45	1.873333	3.873333
up	0	0	0	3.98	4.3	3.1	0	3.793333
up	1.78	1.92	1.66	6.27	7.05	6.33	1.786667	6.55
up	0.02	0	0	1.62	1.77	1.3	0.006667	1.563333
up	0	0	0	1.05	1.02	0.51	0	0.86
up	0	0.04	0.05	2.77	2.37	3.29	0.03	2.81
up	0	0	0	3	3.39	4.06	0	3.483333
up	0.04	0.04	0	1.74	1.83	1.53	0.026667	1.7

up	0.02	0.03	0.01	0.53	0.62	0.46	0.02	0.536667
up	0.14	0.15	0.11	1.29	1.09	1.16	0.133333	1.18
up	0	0	0	2.03	1.78	2.14	0	1.983333
up	3.73	4.98	4.11	10.45	14.99	11.23	4.273333	12.22333
up	2.84	2.42	2.81	11.01	12.46	11.53	2.69	11.66667
up	0.09	0.1	0.09	0.39	0.36	0.3	0.093333	0.35
up	0.57	0.77	0.89	1.87	2.75	2.3	0.743333	2.306667
up	1.93	1.94	1.92	3.61	4.32	3.68	1.93	3.87
up	9.18	9.6	9.9	30.71	37.38	32.7	9.56	33.59667
up	0.36	0.39	0.5	1.33	1.73	1.33	0.416667	1.463333
down	9.06	8.3	9.7	4.1	3.78	4.7	9.02	4.193333
down	7.82	8.06	7.33	3.66	3.19	3.63	7.736667	3.493333
down	15.4	19.06	15.03	3.5	4.93	3.48	16.49667	3.97
down	2.92	2.92	2.96	1.29	1.43	1.35	2.933333	1.356667
down	86.76	76.43	81.23	31.26	33.47	35.5	81.47333	33.41
up	0.03	0.06	0.05	0.91	1.04	0.96	0.046667	0.97
up	0.06	0.1	0.06	0.91	1.42	1.2	0.073333	1.176667
up	10.27	13.84	7.46	43.36	29.39	42.79	10.52333	38.51333
up	0.91	0.46	0.36	4.69	4.2	4.9	0.576667	4.596667
up	21.76	22.95	21.9	184.07	165.01	201.9	22.20333	183.66
up	19.04	18.07	21.1	119.61	216.45	98.71	19.40333	144.9233
down	18.28	24.72	22.51	8.48	8.13	7.94	21.83667	8.183333
down	1.2	1.23	1.24	0	0	0	1.223333	0
up	1.88	1.84	1.47	3.59	3.14	3.76	1.73	3.496667
down	1.24	1.55	1.59	0.14	0.13	0.17	1.46	0.146667
up	2.54	2.22	2.21	6.67	7.35	7.56	2.323333	7.193333
up	120.16	95.66	94.27	184.43	193.43	213.49	103.3633	197.1167
up	175.69	198.03	176.43	391.21	372.53	405.84	183.3833	389.86
up	0.01	0.01	0.02	0.45	0.43	0.57	0.013333	0.483333
up	1.52	1.73	1.72	4.43	4.57	4.53	1.656667	4.51
down	6.27	4.85	5.99	2.15	2.99	2.63	5.703333	2.59
up	15.38	15.94	16.06	29.81	34.96	29.82	15.79333	31.53
up	1.68	1.48	1.36	3.32	2.74	4.28	1.506667	3.446667
down	0.96	0.69	0.99	0	0	0	0.88	0
up	0.56	1.22	0.41	3.15	3.49	3.49	0.73	3.376667
up	1.08	1.15	1.38	8.96	3.98	8.58	1.203333	7.173333
down	6.96	6.81	7.06	2.92	3.19	3.24	6.943333	3.116667
up	7.47	5.22	5.09	17.98	17.35	17.81	5.926667	17.71333
up	0.22	0.26	0.17	16.25	13.81	16.65	0.216667	15.57
up	7.88	10.6	9.85	24.16	24.41	23.27	9.443333	23.94667
down	0.72	0.36	0.49	0	0	0	0.523333	0
up	4.27	5.64	6.66	10.99	11.54	10.44	5.523333	10.99
up	1.1	1.45	1.26	3.31	2.53	3.01	1.27	2.95
up	2.09	2.16	1.89	4.85	5.48	4.52	2.046667	4.95
up	1.31	0.9	1.27	2.26	2.61	2.83	1.16	2.566667
up	0.46	0.49	0.61	3.56	3.19	3.51	0.52	3.42
up	7.4	8.34	7.65	15.69	16.47	14.93	7.796667	15.69667
up	2.56	3.38	3.31	6.51	7.14	6.12	3.083333	6.59
up	2.05	2.18	2.06	7.14	6.93	7.11	2.096667	7.06
up	14.79	16.88	15.91	125.01	120.77	123.94	15.86	123.24
up	0.02	0.02	0.03	0.21	0.2	0.16	0.023333	0.19
up	1.16	1.21	1.31	2.6	3.25	2.64	1.226667	2.83
down	8.07	8.48	9.18	0.31	0.54	0.47	8.576667	0.44
up	0.77	0.89	0.8	2.37	2.61	2.01	0.82	2.33
up	1.93	2.36	1.96	4.09	4.32	5.22	2.083333	4.543333
up	46.87	43.13	42.43	97.24	87.12	99.03	44.14333	94.46333
down	24.42	33.83	32.24	2.39	2.09	2.04	30.16333	2.173333
up	1.7	1.75	1.65	3.45	3.24	3.32	1.7	3.336667

up	8.96	9.75	9.74	20.57	19.9	22.76	9.483333	21.07667
up	0	0	0	0.95	1.34	0.65	0	0.98
down	0.69	1.48	0.56	0	0	0	0.91	0
down	2.43	2.83	2.47	0.63	0.55	0.62	2.576667	0.6
up	179.19	205.79	196.33	456.25	448.16	442.29	193.77	448.9
up	2.57	2.27	2.65	6.62	7.86	7.24	2.496667	7.24
down	9.15	14.36	11.22	1.22	0.79	0.77	11.57667	0.926667
up	0.53	0.63	0.58	1.27	1.25	1.42	0.58	1.313333
down	11.27	12.01	11.69	4.84	4.47	6.09	11.65667	5.133333
up	0.68	1.1	0.91	4.67	4.08	4.47	0.896667	4.406667
up	1.63	1.67	1.98	5.11	5.21	5.32	1.76	5.213333
up	15.51	17.65	16.74	31.27	33.42	30.67	16.63333	31.78667
up	12.05	14.11	13.37	35.03	34.97	38.56	13.17667	36.18667
up	0.21	0.19	0.2	0.83	0.92	0.97	0.2	0.906667
up	4.24	4.31	4.19	9.23	11.61	8.9	4.246667	9.913333
up	0.85	1.09	1.01	3.31	2.73	3.47	0.983333	3.17
up	0.26	0.28	0.28	1.62	1.16	0.94	0.273333	1.24
up	1.07	0.91	0.97	1.92	3.61	3.22	0.983333	2.916667
up	0.52	0.76	0.63	2.25	1.6	2.19	0.636667	2.013333
up	2.15	2.65	1.93	5.06	5.53	5.62	2.243333	5.403333
up	1.83	2.98	2.69	5.61	5.16	4.36	2.5	5.043333
down	2.83	2.22	1.91	0.74	0.79	0.72	2.32	0.75
down	1.09	1.09	1.14	0.42	0.49	0.51	1.106667	0.473333
up	1.98	1.8	2.42	4.6	4.48	4.72	2.066667	4.6
up	1.2	1.45	1.71	4.13	4.66	3.55	1.453333	4.113333
down	1.52	1.14	1.38	0.59	0.43	0.53	1.346667	0.516667
up	1.07	1.54	1.27	3.26	5.64	2.96	1.293333	3.953333
down	3.79	2	3	0.17	0.29	0.18	2.93	0.213333
up	6.84	6.7	6.75	18.01	15.29	20.31	6.763333	17.87
up	10.16	11.15	10.86	23.09	27.46	26.39	10.72333	25.64667
up	3.43	3.95	3.03	7.99	8.64	8.05	3.47	8.226667
up	1.76	1.69	1.54	4.66	4.98	4.3	1.663333	4.646667
down	5.93	6.6	6.4	2.13	1.92	2.03	6.31	2.026667
up	0.37	0.3	0.32	1.24	1.97	1.24	0.33	1.483333
up	2.22	2.32	2.19	9.64	9.26	8.19	2.243333	9.03
up	4.41	4.23	4.22	9.92	8.27	10.24	4.286667	9.476667
up	7.85	8.25	8.01	29.17	23.12	31.97	8.036667	28.08667
up	1.57	1.81	1.93	4.56	6.16	4.81	1.77	5.176667
up	2.54	2.35	2.24	8.02	8.06	8.52	2.376667	8.2
down	9.34	10.22	9.21	4.44	3.02	4.3	9.59	3.92
down	2.01	1.99	1.9	0.44	0.56	0.29	1.966667	0.43
up	0.03	0	0	1.2	0.83	1.25	0.01	1.093333
up	0.3	0.44	0.23	1.19	1.78	1.33	0.323333	1.433333
up	0.01	0	0	2.44	2.25	2.95	0.003333	2.546667
up	0.66	0.95	1.24	3.69	3.75	3.36	0.95	3.6
up	11.5	11.06	11.39	25.63	30.18	25.51	11.31667	27.10667
up	0.46	0.54	0.65	1.52	2.07	1.75	0.55	1.78
up	0.81	0.98	0.82	10.2	11.94	10.27	0.87	10.80333
up	0.07	0.17	0.08	1.11	1.62	1.28	0.106667	1.336667
up	0	0	0	0.23	0.24	0.2	0	0.223333
down	1.42	1.29	1.51	0.47	0.55	0.5	1.406667	0.506667
up	9.11	9.81	9.54	31.86	30.05	32.57	9.486667	31.49333
up	0.28	0.33	0.46	1.9	1.8	2	0.356667	1.9
up	0	0	0	2.32	2.12	3.36	0	2.6
up	0.5	0.36	0.38	1.94	2.6	1.5	0.413333	2.013333
