# Supplementary material for: Elevated ERβ expression driven by low ASB8-mediated ubiquitination in lung adenocarcinoma promotes lymph node metastasis via tumor-associated neutrophils
Source: Cell Death Dis. 2025 Jul 30;16(1):576. doi: 10.1038/s41419-025-07870-z (PMC12311189; doi:10.1038/s41419-025-07870-z)
Supplement: Supplementary file 3 — Data set table 2 [file 41419_2025_7870_MOESM3_ESM.pdf]

Data set table 2

The protein binding to ERβ was screened by LC-MS/MS analysis

Group_ID	Protein_ID	Protein_Qs	Protein_FD	Protein_M	PeptideSe	PepIsUniq	Unique_Pe	Unique_Sp
625_1	sp|Q53FZ2	2.203098	NA	66110.72	VILILPR	1	1	1
982_1	sp|Q9UNL	4.520146	NA	21067.2	QQSEEDLL	1	1	1
229_1	sp|P62873	2.270111	NA	37353.01	VHAIPLR	1	1	1
838_1	sp|Q53SF7	2.203098	NA	123791.3	SLNDLGLR	1	1	1
347_1	sp|O14983	4.520146	NA	110181.6	VDQSILTG	1	1	1
136_1	sp|P42677	3.478753	NA	9454.836	DLLHPSPE	1	1	1
330_1	sp|Q5JVS0	4.520146	NA	45757.57	VFDAFDQ	1	1	1
535_1	sp|Q9H765	3.235797	NA	31622.14	LVALLVR	1	1	1
179_1	sp|Q96CN	2.364944	NA	87757.31	SVALASGL	1	1	1
566_1	sp|P51570	9.040291	NA	42245.57	EEFGAEPE	1;1	2	2
537_1	sp|P78332	4.520146	NA	128565.2	EEGGLDFL	1	1	1
205_1	sp|O94992	2.109454	NA	40598.48	DFSETYER	1	1	1
20_1	sp|Q9UPT8	2.502609	NA	140169.5	MLADDAE	1	1	1
729_1	sp|Q6P158	2.203098	NA	155506.8	ISAISVAER	1	1	1
118_1	sp|O95714	2.203098	NA	526895.2	LVAALQGI	1	1	1
405_1	sp|Q8NCX	2.109454	NA	128680.6	EMQMLAK	1	1	1
688_1	sp|Q9HCE	2.532408	NA	113599.5	VGSVEEFQ	1	1	1
388_1	sp|Q5JTH9	2.45199	NA	143611.1	VLATQPGI	1	1	1
341_1	sp|P00450	2.45199	NA	122127.6	DIFTGLIGP	1	1	1
811_1	sp|P05114	4.520146	NA	10652.59	EDLPAENC	1	1	1
817_1	sp|Q9NUP	6.951871	NA	21820.46	AIELLEK;TE	1;1	2	2
153_1	sp|Q96FZ7	2.099037	NA	23470.3	IAQQLERE	1	1	1
852_1	sp|Q13111	2.604087	NA	106843.6	EDTGDQQ	1	1	1
1038_1	sp|Q8WZ7	2.203098	NA	41685.97	LLTTLQSA	1	1	1
738_1	sp|Q14966	4.520146	NA	220488	YGYTEDPL	1	1	1
952_1	sp|Q9Y2Q	2.532408	NA	20829.8	LLDLELTSR	1	1	1
874_1	sp|Q9Y3T9	3.478753	NA	84866.15	VLAFLVLSI	1	1	1
293_1	sp|Q9UI08	3.478753	NA	44592.22	EEIIDAIR	1	1	1
782_1	sp|Q9BZV	2.364944	NA	49722.89	FFQEFK	1	1	1
1025_1	sp|Q13523	7.755943	NA	116915.7	AELDNELN	1;1	2	2
749_1	sp|Q9Y2Q	2.474409	NA	131285.5	IWILTGDK	1	1	1
427_1	sp|Q8NH4	2.116912	NA	36459.48	MIADFLVE	1	1	1
425_1	sp|P83881	8.917649	NA	12432.81	DSLYAQGI	1;1;1	3	3
1039_1	sp|Q9NZP	3.478753	NA	35280.65	VLFDILK	1	1	1
112_1	sp|P68431	3.235797	NA	15394.48	EIAQDFK	1	1	1
1000_1	sp|Q14554	2.474409	NA	59556.1	NNVLVLYS	1	1	2
919_1	sp|Q96IZ7	5.716072	NA	38654.4	EEDQATLV	1;1	2	2
593_1	sp|A3QJZ6	3.235797	NA	55452.63	NILETVYPD	1	1	1
806_1	sp|Q99575	3.478753	NA	114635.8	IPILLIQQP	1	1	1
23_1	sp|Q9HBM	6.367911	NA	27604.42	DFLLDIAR;	1;1	2	2
382_1	sp|Q5CZC	2.05055	NA	780119.2	LANSLIR	1	1	1
98_1	sp|Q8IZP2	10.96371	NA	27389.69	AIDLFTDA	1;1;1	3	3
655_1	sp|Q10570	13.6156	NA	160782	ESLAEEHE	1;1;1;1	4	4
936_1	sp|A0PJZ3	2.003925	NA	51022.78	ALYEAIR	1	1	1
693_1	sp|Q99829	3.478753	NA	59021.54	SDPFLEFFF	1	1	2
825_1	sp|B2RPK0	4.520146	NA	24222.81	LGEMWNN	1	1	2
540_1	sp|P19022	3.235797	NA	99747.28	SAAPHPGI	1	1	1
930_1	sp|P61011	3.478753	NA	55668.29	DVQELLTQ	1	1	1
577_1	sp|Q9Y3B9	4.520146	NA	31464.51	GVVQLFNA	1	1	1
428_1	sp|Q5TCQ	2.203098	NA	162848.2	NWEMAYT	1	1	1
95_1	sp|O43159	5.481583	NA	50683.1	LFQEDPEA	1;1	2	2
255_1	sp|Q9NQS	8.917232	NA	38482.86	EGDNILPD	1;1;1	3	3
977_1	sp|Q9BY44	3.478753	NA	64949.18	TITYQAVP	1	1	1
294_1	sp|A0MZ6	2.889158	NA	71595.69	LTQQLEEE	1	1	1
358_1	sp|P49590	2.889158	NA	56851.66	GLAPEVAD	1	1	1

296_1	sp|Q92576	3.235797	NA	229338.8	TDNVEVTD	1	1	1
2_6	sp|P04259	16.52525	NA	60030.29	AEAESWY(	0;1;0;0;0;0;	4	4
251_1	sp|Q8WVK	12.51904	NA	18848.87	QITEEDLE(	1;1;1	3	3
185_1	sp|Q12797	3.478753	NA	85809.45	LGIYDADG	1	1	1
863_1	sp|Q8TER5	2.45199	NA	164555.3	VLDIFEQR	1	1	1
766_1	sp|O00422	2.203098	NA	17549.97	ELTSLVK	1	1	1
16_1	sp|Q2M1P	2.964811	NA	150494.9	QLTLQQK	1	1	1
690_1	sp|Q8TDI7	3.235797	NA	102544.3	LANEETIK	1	1	1
1031_1	sp|Q9ULJ8	3.235797	NA	123266.8	LELFPVELE	1	1	1
395_1	sp|O60293	3.478753	NA	226214.4	TLNFEDQT	1	1	1
278_1	sp|Q9Y6B6	4.520146	NA	22395.51	LVFLGLDN	1	1	1
123_1	sp|Q9P202	4.520146	NA	96498.97	VGDQILEV	1	1	1
574_1	sp|O15090	2.45199	NA	141327.6	GPQSLDKI	1	1	1
700_1	sp|Q9BTC(	2.270111	NA	243723.2	EKPLEEPD/	1	1	1
82_1	sp|Q99501	2.964811	NA	72671.85	LGLLAPR	1	1	1
637_1	sp|Q8N7H	6.951871	NA	59939.11	LLEEEIQAF	1;1	2	2
353_1	sp|P0DOX	4.520146	NA	23364.42	VDNALQS	1	1	1
252_1	sp|P22557	2.431726	NA	64591.75	VGNAALN	1	1	1
801_1	sp|Q00577	3.478753	NA	34889.4	FFFDVGSN	1	1	1
1037_1	sp|Q8N5N	3.235797	NA	18313.37	DVLDFYN\	1	1	1
256_1	sp|P63241	9.950348	NA	16821.4	IVEMSTSK;	1;1;1	3	3
600_1	sp|A1A4V9	2.210366	NA	37897.37	ELEQLQGL	1	1	1
464_1	sp|Q9C0J8	4.520146	NA	145799.3	GLLPTPDE	1	1	1
547_1	sp|O94967	2.003925	NA	101884.2	LILDFLNSK	1	1	1
100_1	sp|Q9UPN	4.38058	NA	140433.1	VFDYFEGA	1;1	2	2
1055_1	sp|Q92731	12.78093	NA	59177.65	LANLLMLL	1;1;1;1	4	4
8_1	sp|O60814	9.040291	NA	13881.56	AMGIMNS	1;1	2	15
102_1	sp|P10412	23.92428	NA	21852	ALAAAGYI	1;1;1;1;1;1	6	7
761_1	sp|P52594	4.520146	NA	58224.16	SISMTTFT(	1	1	1
396_1	sp|Q9ULW	3.235797	NA	85599.99	STEEQELEI	1	1	1
840_1	sp|Q9BVC(	4.520146	NA	26193.58	EAPVDVLT	1	1	1
398_1	sp|O95218	7.165999	NA	37382.02	LDEDEDED	1;1	2	2
889_1	sp|Q8WXE	3.235797	NA	101101.5	LGLNDILV	1	1	1
862_1	sp|Q08AD.	2.177482	NA	167983.8	SDANNFLI	1	1	1
908_1	sp|Q13895	3.478753	NA	49570	FYNLVLLPI	1	1	1
991_1	sp|Q9P2N!	3.478753	NA	118644.7	LSQLQVE/	1	1	1
460_1	sp|P06312	4.520146	NA	13371.59	LLIYWASTI	1	1	1
232_1	sp|Q9Y676	3.478753	NA	29377.01	YLESEEYQI	1	1	1
834_1	sp|P09001	3.235797	NA	38608.07	ATSILEFYR	1	1	1
590_1	sp|O94925	3.235797	NA	73413.9	DGPGETD/	1	1	1
900_1	sp|Q14331	2.128758	NA	29154.12	YLGINSDG	1	1	1
13_1	sp|O60684	4.423905	NA	59991.45	FVEFLK;IEF	1;1	2	2
732_1	sp|Q9BRK5	2.431726	NA	41780.46	VDEETQE\	1	1	1
162_1	sp|O75683	4.520146	NA	41425.59	VEVSEDEP	1	1	1
17_3	sp|P68371	4.520146	NA	49799	ALTVPELT(	0;0;0;0;0;1;	1	1
17_2	sp|Q13509	6.471595	NA	50400.25	ALTVPELT(	0;1;0;0;0;0;	2	2
482_1	sp|O75934	13.56044	NA	26115	EAAAALVE	1;1;1	3	3
865_1	sp|P02652	3.235797	NA	11167.9	KAGTELVN	1	1	1
392_1	sp|Q7Z5H4	2.326087	NA	40751.62	VIVFLNK	1	1	1
923_1	sp|Q6UWF	2.364944	NA	28290.26	ALVQQGL	1	1	1
677_1	sp|Q9H5H	4.520146	NA	60190.75	AFGDSSYL	1	1	1
122_1	sp|Q8WXV	2.431726	NA	89749.6	QNSLILK	1	1	1
734_1	sp|P50579	4.520146	NA	52858.14	ESGASVDE	1	1	1
505_1	sp|Q9H8G	4.520146	NA	38343.82	STDSSSVS	1	1	1
88_1	sp|Q9NXE8	2.128758	NA	49616.99	LSAEELER	1	1	1
390_1	sp|Q9NWH	6.71455	NA	117078.5	FNDFDHR;	1;1	2	2
636_1	sp|Q9Y3E1	4.520146	NA	22606.01	AGDLVFAI	1	1	1
81_1	sp|Q5SNV9	2.177482	NA	162321.4	LGLPGAGI	1	1	1

1004_1	sp|Q8WVC	11.24339	NA	75359.29	APLQGDH	1;1;1		3	3
443_1	sp|Q8NEJ9	3.235797	NA	35872.01	TAVTGSLS		1	1	1
469_1	sp|O60673	2.431726	NA	352554.2	LQEILK		1	1	1
87_1	sp|P12235	2.964811	NA	33043.22	EQGFLSFW	1;0;0;0		1	1
103_1	sp|Q8NDH	2.45199	NA	801410.9	ISIVTNWN		1	1	1
886_1	sp|Q9H9J2	2.344623	NA	37511.71	LIAEGPGE		1	1	1
105_1	sp|Q5T75C	2.532408	NA	26219.27	TFGVSPLR		1	1	1
888_1	sp|O15226	11.2347	NA	77624.22	EGLGLDVE	1;1;1		3	3
948_1	sp|Q9BV38	2.889158	NA	47375.01	DLFDFSTR		1	1	1
630_1	sp|P54198	3.235797	NA	111763.9	QQQQQLI		1	1	1
374_1	sp|Q8IXI1|I	3.235797	NA	68074.69	VPIILVGNK		1	1	1
587_1	sp|Q8N9T8	13.56044	NA	82547.52	AQEEADYI	1;1;1		3	3
31_1	sp|P78362	4.520146	NA	77478.15	AADLLVNI	1;0		1	1
465_1	sp|P61619	2.326087	NA	52230.51	AFSPTTVN		1	1	1
928_1	sp|Q9UJU6	4.520146	NA	48177.57	VAGTGEG		1	1	1
101_1	sp|Q9C0C2	2.889158	NA	181685	VSAPGVLT		1	1	1
356_1	sp|Q9P0N9	2.099037	NA	33949.51	VLLGILPPH		1	1	1
228_1	sp|O95447	2.109454	NA	76457.68	TLQVEVK		1	1	1
144_1	sp|P27708	14.74313	NA	242829.5	ELSDLESAI	1;1;1;1		4	4
925_1	sp|Q8TAD8	7.998898	NA	45750.33	DRDTQNL	1;1		2	2
328_1	sp|Q9UQ8	7.022754	NA	91305.52	DSLEEGELI	1;1		2	2
342_1	sp|O14646	3.478753	NA	196566.7	SNLEGSLK		1	1	1
286_1	sp|Q9BV86	2.364944	NA	25370.84	LLLPLFR		1	1	1
569_1	sp|Q7RTV0	2.203098	NA	12396.96	TDLFYER		1	1	1
36_1	sp|O14880	2.128758	NA	16505.57	IASGLGLA		1	1	1
816_1	sp|P55196	3.478753	NA	206675.9	VSSTATTQ		1	1	1
430_1	sp|P20339	2.253669	NA	23643.82	LVLLGESA		1	1	1
598_1	sp|Q96BK5	3.478753	NA	37011.98	GLGAQEQ		1	1	1
32_1	sp|Q5TYW	2.058961	NA	122195.6	LQLDIK		1	1	1
223_1	sp|O43414	2.326087	NA	37213.68	FSSYLLSR		1	1	1
34_1	sp|Q9H6R4	2.532408	NA	127512.9	ETSSTGEE		1	1	1
810_1	sp|Q8TBF8	2.502609	NA	42365.1	DSNHQLQ		1	1	1
200_1	sp|Q86W2	2.431726	NA	118806.6	VMEELLK		1	1	1
580_1	sp|O60783	2.502609	NA	15129.02	ILQDVADE		1	1	1
434_1	sp|Q96P70	2.749922	NA	115888.7	ELGENLDQ		1	1	1
935_1	sp|Q9P035	7.363556	NA	43131.56	FSFTLPYP	1;1;1		3	3
436_1	sp|P82914	3.478753	NA	29823.44	IVANPEDT		1	1	1
858_1	sp|Q96EL2	2.964811	NA	19002.87	TVEDVFLR		1	1	1
338_1	sp|O14893	2.364944	NA	31565.07	TPQEYLR		1	1	1
243_1	sp|O15123	2.05055	NA	56882.93	LQVLENIM		1	1	1
514_1	sp|Q71SY5	2.128758	NA	78121.42	LTRSLPCQ		1	1	1
496_1	sp|P02656	4.520146	NA	10845.5	DALSSVQE		1	1	1
325_1	sp|Q9UDW	2.109454	NA	7303.826	LYSLLFR		1	1	1
942_1	sp|Q14558	3.235797	NA	39368.63	GQDIFIIQT		1	1	1
818_1	sp|Q96RP9	4.520146	NA	83418.44	GIVDLIEER		1	1	1
510_1	sp|O60287	2.224085	NA	254227.2	ILLLLR		1	1	1
757_1	sp|Q9NYT6	2.109454	NA	91861.76	QGNLGEK		1	1	1
562_1	sp|Q9UPT5	2.203098	NA	83330.1	VTDYIAEK		1	1	1

Coverage	Peptide_Q	Sameset	Descriptioi	Abundanc	iBAQ	GeneName
0.0119	4903	-	Acyl-coen	78797.32	2188.815	ACSM3
0.0757	45871	-	Translocor	0	0	SSR3
0.0206	4240	sp|P16520	Guanine n	145974.2	11228.79	GNB1
0.0071	7645	-	Cordon-bl	286743.7	4216.82	COBLL1
0.015	41605	sp|P16615	Sarcoplasr	0	0	ATP2A1
0.131	28707	-	40S riboso	702836.5	140567.3	RPS27
0.0194	12420	-	Intracellula	140160.3	5606.413	HABP4
0.0243	3368	-	Ankyrin re	164993.9	7499.721	ASB8
0.0142	13847	-	GRIP and (	514629.4	9530.174	GCC1
0.0689	44764;283	-	Galactokin	1153967	60735.1	GALK1
0.0089	17432	-	RNA-bind	24060.94	300.7618	RBM6
0.0223	14709	sp|Q96MH	Protein HE	0	0	HEXIM1
0.0107	37695	-	Zinc finger	0	0	ZC3H4
0.0065	10140	sp|Q9H2U	Putative A	245676.1	3109.825	DHX57
0.0017	3971	-	E3 ubiquiti	529097.6	2186.354	HERC2
0.0136	47998	-	Coiled-coi	28310.01	353.8752	CCDC150
0.012	32319	-	Helicase M	0	0	MOV10
0.0077	12366	-	RRP12-like	511175.6	7002.405	RRP12
0.0103	23202	-	Ceruloplas	0	0	CP
0.11	23829	-	Non-histo	521543.9	86923.98	HMGN1
0.1015	4603;3250	-	Protein lin	1412722	94181.47	LIN7C
0.0448	20454	-	Charged m	1587876	144352.4	CHMP6
0.0115	23845	-	Chromatin	741380.3	16475.12	CHAF1A
0.0332	28419	-	Deoxyribo	1135426	56771.29	DNASE2B
0.0056	31220	-	Zinc finger	154501.8	1443.942	ZNF638
0.0481	15483	-	28S riboso	168183.4	11212.22	MRPS28
0.012	13406	-	Nucleolar	0	0	NOC2L
0.0192	10692	-	Ena/VASP-	279589.5	12156.06	EVL
0.0136	5873	-	UBX doma	37799.18	1718.144	UBXN6
0.0199	26279;119	-	Serine/thre	289191.6	5258.029	PRPF4B
0.0069	10149	-	Phospholip	7782584	117917.9	ATP8A1
0.0278	18453	-	Olfactory r	22556.16	2255.616	OR4K15
0.217	7375;8293	-	60S riboso	5944865	990810.9	RPL36A
0.0227	5986	-	Olfactory r	34390.22	4298.778	OR5AC2
0.0515	6094	sp|Q16695	Histone H3	60198571	10033095	H3C1
0.0173	14873;149	-	Protein dis	1335524	44517.48	PDIA5
0.0838	33518;518	-	Serine/Arg	240139.1	13341.06	RSRC1
0.0748	64270	-	PRAME far	0	0	PRAMEF22
0.0107	24777	-	Ribonuclea	0	0	POP1
0.1195	10869;587	-	Transcripti	79824.34	4989.021	TAF9B
0.001	3541	-	Fibrous sh	967892.9	2425.797	FSIP2
0.1292	18296;368	sp|P50502	Putative pı	2705864	180391	ST13P4
0.0347	47468;152	-	Cleavage a	161980	1999.753	CPSF1
0.0158	5420	-	Glucoside	981806.8	37761.8	GXYLT2
0.0168	21243;212	-	Copine-1	55211.7	2760.585	CPNE1
0.0616	37697;370	sp|P09429	Putative hi	0	0	HMGB1P1
0.0199	50332	-	Cadherin-	24497.05	699.9157	CDH2
0.0218	30994	-	Signal recc	281630.7	7823.075	SRP54
0.039	23816	-	RRP15-like	439067.5	27441.72	RRP15
0.0135	59296	-	Membrane	0	0	MAGI3
0.0461	48647;456	-	Ribosomal	172327.5	7180.314	RRP8
0.1215	45239;415	-	Cell death	587922	27996.29	AVEN
0.0291	51898	-	Eukaryotic	0	0	EIF2A
0.0143	20565	-	Shootin-1	239700.2	5447.732	SHTN1
0.0178	9320	sp|P12081	Histidine-	471248.9	14280.27	HARS2

0.0059	30085	-	PHD finge	133474.5	1160.648	PHF3
0.3121	24335;356	-	Keratin, ty|	2760596	76683.22	KRT6B
0.2194	21477;211	-	U4/U6.U5	14867178	2477863	SNRNP27
0.0237	51877	-	Aspartyl/as	0	0	ASPH
0.0053	13479	-	Rho guani	356615.2	4885.139	ARHGEF40
0.0458	3627	-	Histone de	290455.2	20746.8	SAP18
0.0052	6491	-	Kinesin-lik	1276644	14844.7	KIF7
0.0088	8953	-	Transmem	2502485	61036.22	TMC2
0.0091	24602	sp|Q96SB3	Neurabin-	137658.2	2549.226	PPP1R9A
0.0085	52133	-	Zinc finger	316218.3	2901.086	ZFC3H1
0.0556	20644	sp|Q9NR3	GTP-bindi	94464.57	8587.688	SAR1B
0.0121	23745	-	Whirlin OS	793620.7	16196.34	WHRN
0.0308	63679	-	Zinc finger	0	0	ZNF536
0.0054	32514	-	Death-ind	874328.4	6777.739	DIDO1
0.0103	1641	-	GAS2-like	129274.6	2938.059	GAS2L1
0.0508	36122;427	-	RNA polyr	415265.6	11535.16	PAF1
0.0935	56333	sp|P01834	Immunogl	342778.9	34277.89	NA
0.0153	7090	-	5-aminole	1554672	40912.42	ALAS2
0.028	15513	-	Transcripti	77153.17	5143.545	PURA
0.0823	42345	-	39S riboso	400077.9	36370.72	MRPL50
0.1494	7954;5091	-	Eukaryotic	4159898	519987.3	EIF5A
0.0363	36066	-	Cilia- and	0	0	CFAP119
0.0082	25908	-	pre-mRNA	711249.2	11854.15	WDR33
0.0098	15646	-	WD repeat	98653.44	2242.124	WDR47
0.0189	34904;306	-	SR-related	320527.3	6284.85	SCAF8
0.083	33830;275	-	Estrogen r	1785519	68673.79	ESR2
0.2063	48081;485	sp|P58876	Histone H2	490946.4	44631.49	H2BC12
0.242	18336;417	-	Histone H1	1.58E+08	13172015	H1-4
0.0302	54875	-	Arf-GAP d	0	0	AGFG1
0.012	17420	-	Targeting	348036.5	8286.583	TPX2
0.0494	28955	-	Transmem	750105.7	75010.57	TMEM109
0.0818	42932;390	-	Zinc finger	72780.06	3830.529	ZRANB2
0.0099	11846	-	Stonin-2 C	0	0	STON2
0.0074	29568	-	Calmoduli	167229.6	1944.53	CAMSAP2
0.0206	19945	-	Bystin OS=	0	0	BYSL
0.0094	18744	-	RNA-bind	82378.27	1790.832	RBM27
0.0744	19236	-	Immunogl	412541.2	82508.25	IGKV4-1
0.0388	31408	-	28S riboso	118863.9	8490.278	MRPS18B
0.0259	17861	-	39S riboso	41850.47	1992.88	MRPL3
0.0224	37696	-	Glutamina	0	0	GLS
0.0504	32247	-	Protein FR	191210.6	14708.51	FRG1
0.0392	3317;5199	sp|P52294	Importin s	265506.6	11062.77	KPNA6
0.0359	40412	-	45 kDa cal	1213875	63888.17	SDF4
0.0305	23074	-	Surfeit locu	590729.5	32818.31	SURF6
0.2517	46451;369	-	Tubulin be	2334248	111154.7	TUBB4B
0.2222	46451;343	-	Tubulin be	254437.1	12116.05	TUBB3
0.1511	28448;153	-	Pre-mRNA	985161.5	70368.68	BCAS2
0.23	59654	-	Apolipopr	14487.49	2897.497	APOA2
0.0196	5299	-	Vomerona	1956303	217367	VN1R5
0.0308	6407	-	Dehydrog	0	0	DHRS11
0.0185	19565	-	Zinc finger	40324.73	1089.858	ZNF768
0.0092	4606	-	Progester	1022445	16491.05	PIBF1
0.023	19028	-	Methionin	240534.8	8017.826	METAP2
0.0443	44121	-	Caspase a	99951.06	9086.46	CAAP1
0.0188	10175	-	Pre-mRNA	2687346	111972.8	CWC25
0.0164	10325;130	-	SAFB-like	1775721	34818.06	SLTM
0.0394	4781	-	Hepatoma	900380.3	81852.76	HDGFL3
0.0054	526	-	Uncharact	2355811	29084.09	C1orf167

0.0511	39287;913	-	RNA polyr	2287535	63542.65	LEO1
0.0444	36797	-	Neuroguic	199296.5	12456.03	NGDN
0.0019	1814	sp|Q8WXH	DNA polyr	439730.6	2601.956	REV3L
0.1309	21920;200	-	ADP/ATP t	69022.13	3286.768	SLC25A4
0.003	59165	-	Leucine-ri	0	0	CCDC168
0.0572	53074	-	39S riboso	0	0	MRPL44
0.032	7208	-	Skin-speci	1237144	103095.4	XP32
0.0449	11966;279	-	NF-kappa	1288087	28001.89	NKRF
0.0185	12569	-	WD repeat	53445.41	2812.916	WDR18
0.0088	20598	-	Protein HII	0	0	HIRA
0.0146	10454	-	Mitochonc	0	0	RHOT2
0.0555	38292;445	-	Protein KR	0	0	KRI1
0.0291	28736;786	-	SRSF prote	182887.6	6096.252	SRPK2
0.0231	20850	-	Protein tra	444605.2	26153.25	SEC61A1
0.0465	54382	-	Drebrin-lik	0	0	DBNL
0.0069	24429	-	182 kDa ta	107625.3	1251.457	TNKS1BP1
0.0512	44402	-	TBC1 dom	7590.88	316.2867	TBC1D7
0.0104	4649	-	Lebercilin-	510290.8	11339.8	LCA5L
0.0211	13460;232	-	CAD prote	1077371	9975.655	CAD
0.0354	47227;368	-	Smad nucl	0	0	SNIP1
0.0255	14752;282	sp|P21127	Cyclin-dep	2202744	53725.46	CDK11A
0.0047	5975	-	Chromodc	378049.1	3600.468	CHD1
0.0314	7024	-	N-termina	0	0	NTMT1
0.0636	9984	-	PHD finge	460708.1	57588.52	PHF5A
0.0855	29715	-	Microsoma	0	0	MGST3
0.0093	49114	-	Afadin OS	325483.5	3191.014	AFDN
0.0512	17047	sp|P51148	Ras-relate	326739.4	23338.53	RAB5A
0.0427	35172	-	PIN2/TERF	0	0	PINX1
0.0057	1254	-	Zinc finger	367371.6	5174.248	ZNF658
0.0237	11279	-	ERI1 exorit	382276.2	23892.26	ERI3
0.0131	39353	-	Nucleolar	159139.3	3247.74	NOL6
0.0326	34626	-	Protein FA	17742.84	611.8222	FAM81A
0.0067	6594	-	NACHT, LF	1984033	35429.16	NLRP13
0.1172	44573	-	28S riboso	0	0	MRPS14
0.0106	29036	-	Importin-9	348459.4	8499.011	IPO9
0.0635	23592;354	-	Very-long	479927.1	28231.01	HACD3
0.035	13223	-	28S riboso	998636.7	52559.83	MRPS15
0.0479	11578	-	28S riboso	163172.9	13597.74	MRPS24
0.025	8471	-	Gem-asso	570002.7	40714.48	GEMIN2
0.0343	55896	-	Angiopoie	0	0	ANGPT2
0.0241	56230	-	Mediator c	0	0	MED25
0.1616	46757	-	Apolipopr	0	0	APOC3
0.1111	8676	-	Cytochron	0	0	UQCR10
0.0337	34059	-	Phosphori	33630.72	1401.28	PRPSAP1
0.012	14601	-	Elongatior	82011.07	1822.468	GFM1
0.0026	1681	-	Nucleolar	36349.36	318.854	URB1
0.0137	23849	-	Zinc finger	0	0	ZNF226
0.0109	9796	-	Exocyst co	2395297	54438.57	EXOC7
